# A Systematic Review and Individual Patient Data Network Analysis of the Residual Symptom Structure Following Cognitive-Behavioral Therapy and Escitalopram, Mirtazapine and Venlafaxine for Depression

**DOI:** 10.3389/fpsyt.2022.746678

**Published:** 2022-02-01

**Authors:** Aoife Whiston, Amy Lennon, Catherine Brown, Chloe Looney, Eve Larkin, Laurie O'Sullivan, Nurcan Sik, Maria Semkovska

**Affiliations:** ^1^Department of Psychology, University of Limerick, Limerick, Ireland; ^2^Department of Psychology, University of Southern Denmark, Odense, Denmark

**Keywords:** depression, residual symptomology, cognitive-behavioral therapy (CBT), antidepressants, network psychometrics

## Abstract

**Objective:**

Consistent evidence suggests residual depressive symptomology are the strongest predictors of depression relapse following cognitive-behavioral therapy (CBT) and antidepressant medications (ADM's). Psychometric network models help detecting and understanding central symptoms that remain post-treatment, along with their complex co-occurrences. However, individual psychometric network studies show inconsistent findings. This systematic review and IPD network analysis aimed to estimate and compare the symptom network structures of residual depressive symptoms following CBT, ADM's, and their combination.

**Methods:**

PsycINFO, PsycArticles, and PubMed were systematically searched through October 2020 for studies that have assessed individuals with major depression at post-treatment receiving either CBT and/or ADM's (venlafaxine, escitalopram, mirtazapine). IPD was requested from eligible samples to estimate and compare residual symptom psychometric network models post-CBT and post-ADM's.

**Results:**

In total, 25 from 663 eligible samples, including 1,389 patients qualified for the IPD. Depressed mood and anhedonia were consistently central residual symptoms post-CBT and post-ADM's. For CBT, fatigue-related and anxiety symptoms were also central post-treatment. A significant difference in network structure across treatments (CBT vs. ADM) was observed for samples measuring depression severity using the MADRS. Specifically, stronger symptom occurrences were present amongst *lassitude-suicide* post-CBT (vs. ADM's) and amongst *lassitude-inability to feel* post-ADM's (vs. CBT). No significant difference in global strength was observed across treatments.

**Conclusions:**

Core major depression symptoms remain central across treatments, strategies to target these symptoms should be considered. Anxiety and fatigue related complaints also remain central post-CBT. Efforts must be made amongst researchers, institutions, and journals to permit sharing of IPD.

**Systematic Review Registration:** A protocol was prospectively registered on PROSPERO (CRD42020141663; https://www.crd.york.ac.uk/PROSPERO/display_record.php?RecordID=141663).

## Introduction

Depression is the leading cause of disability worldwide, reaching this status two decades earlier than originally projected (1). Much of depression's disease burden is attributed to its high relapse and recurrence rates (2). Current relapse and recurrence estimates demonstrate that, following a first depressive episode, 50–60% of individuals experience another, for subsequent episodes' recurrence/relapse risks reach 70–90% (3). Moreover, despite equal effectiveness of depression treatments, even after effective cognitive behavioral therapy (CBT), antidepressant treatments (ADM's), or combined (CBT + ADM's) treatment, 30–54% of patients will experience a depression relapse or recurrence (4–6).

Substantial research has focused on determining clinical and demographic predictors of depression relapse and recurrence. For example, Burcusa and Iacono (7) identified; severity of first episode, stressful life events and specific symptom developments as predictors of depression relapse or recurrence. Buckman et al. (8) also showed childhood maltreatment, residual symptoms, comorbidity, and rumination as predictors of depressive relapse or recurrence. Although specific predictors identified by individual studies vary, residual depressive symptomology has been consistently shown as the most robust predictor of depression relapse and recurrence across ADM, CBT, and combined treatment studies (9–11).

Residual symptomology refers to the persistence following treatment of some depressive symptoms, which number and/or severity does not reach the threshold to fulfill the syndromal definition of major depressive disorder [MDD (12)]. Residual symptoms predict not only higher depression relapse and recurrence rates, but also post-treatment functional impairment, e.g., social relationships (6). Following successful ADM treatment, 70–90% of patients who reach remission, experience at least one residual symptom (13). Furthermore, at 1-year post-treatment, 60% of patients still show residual symptomology (14). While literature on specific symptoms is still scarce, fatigue, cognitive (e.g., indecisiveness, difficulty concentrating), weight and sleep problems have been consistently identified as residual symptoms post-ADM treatment (14–17). Following CBT, depressed mood, anxiety, sleep problems, and feelings of guilt remain in large percentages (18, 19).

Currently, prevailing methodologies for quantifying and studying residual depressive symptomology are either sum individual symptom severity scores, or list residual symptoms (17). This tendency to count, sum or list symptoms reflects traditional models of disease that persist in modern psychiatry research (20). Core to these models are two assumptions. First, depressive symptoms have a *common cause*, rendering all residual symptoms equipotent, interchangeable indicators of potential relapse or recurrence (20). Second, depressive symptoms are *locally independent*—correlations amongst residual symptoms are spurious, with covariance a consequence of the underlying MDD (21). Thus, the number of symptoms is prioritized over their type. Recent evidence questions the validity of these models for depression (22). For example, in a study of 3,703 MDD patients, over 1,000 unique depression symptom profiles were shown, with symptoms differing in precipitants, biology and impairment levels (23). Residual symptoms are thus unlikely a consequence of a common cause. Furthermore, symptoms associated with depression do co-vary independently from depression as a latent variable (e.g., sleep problems leading to fatigue), violating assumptions of local-independence (21). A continued focus on sum-scores slows down progress in depression relapse/recurrence research as they obfuscate complex relationships amongst symptoms that can remain post-treatment (1).

In the last decade, an alternative methodology—psychometric network modeling has begun to transform understandings of depressive symptomology (24). In network models, psychological constructs, such as residual symptoms, are represented as systems of autonomous, interacting components. Networks comprise of nodes- objects of study (e.g., residual symptoms), and edges- connections or relationships between nodes/residual symptoms (25). As such, networks enable visualization of partial correlations amongst specific residual symptoms and in some cases, the direction of these associations (21). Upon estimating networks, secondary features can also be derived to provide further insight into residual symptom co-occurrences. These include network density and node centrality metrics—strength and expected influence. Network density measures the proportion of connections in a network that are actual connections, with greater density associated with greater symptom severity (26). Centrality metrics of strength and expected influence both denote the sum of the correlation coefficients of the edges connected to a node/residual symptom (27). Highly central residual symptoms are associated with a greater influence on spreading/maintaining symptom activation throughout the network. Therefore, identifying central residual symptoms may yield important information for relapse/recurrence prevention. Importantly, unlike traditional disease models, network models do not presume equipotency of residual symptoms and can accommodate their unique roles by estimating differences in risk factors, interactions and consequences (26).

To date, psychometric network models predominantly focus on estimating symptom networks of individuals fulfilling the syndromal definition of MDD. In general, “loss of energy,” “low mood,” and “loss of interest” demonstrate to be highly central symptoms (28–31). Variability of symptom centrality across populations is also observed, with “self-blame” and “self-hatred” central for depressed adolescents; and “slow-thinking,” “loss of energy,” and “hopelessness” for geriatric depression (32, 33). Moreover, no difference is shown when comparing the centrality of DSM criteria and non-DSM depression symptoms, further highlighting the importance of assessing a large number of disaggregated depressive symptoms (23). Regarding network density for current MDD, studies show greater density is associated with greater depression severity, impairments, and poorer treatment outcomes (26). Whilst these studies advance our understanding of symptom occurrences in current MDD, only a handful of studies have focused on residual depressive symptom networks post-treatment.

Of studies estimating psychometric network models to analyse residual symptomology, four looked at residual symptoms following ADM treatment (29, 30, 34, 35). Estimating cross-sectional networks; “anhedonia” and “depressed mood” were identified as central residual symptoms post-ADM's. Strong symptom interactions remained amongst “depressed mood–suicidality” and “depressed mood-fatigue.” Moreover, network density was shown to increase from baseline- to post-ADM treatment (29, 30, 34, 35). Estimating longitudinal networks, Groen et al. (35) showed; “feeling everything is an effort” was a central symptom associated with depression persistence (vs. remission) post-ADM's. They also observed network density was not associated with depression persistence. Interestingly, these findings regarding network density are at odds with research emphasizing greater density implies greater severity, leading researchers to question if these are potential unique effects of the ADM's (35). Although many ADM's have been investigated using network models, the current review solely focuses on three new-generation ADM's, that is—escitalopram, mirtazapine and venlafaxine, due to their superior efficacy over other ADM's for acute-phase MDD treatment (36). Moreover, pharmacovigilance programs show these ADM's are the most widely prescribed (37).

Among psychotherapies, effects of CBT and Interpersonal Psychotherapy (IPT) on residual symptoms networks have been investigated. Following CBT, “trouble concentrating” was more central in individuals who relapse [vs. remitters (38)]. Conversely, “trouble relaxing” was more central for remitters (vs. relapse). Following IPT, “feeling disliked” and “concentration difficulties” were the most central residual symptoms (39). Strong symptom interactions also remained amongst “loneliness-sleep problems” and “inability to get going-crying,” and network density remained the same throughout treatment. Although, CBT and IPT demonstrate equivalent overall treatment effects, CBT was chosen for this review as the therapy remains more prescribed than IPT and other psychotherapies (40). CBT also shows effects lasting beyond the end of active treatment, potentially reducing risk of relapse and residual symptoms (5).

Current findings from both traditional disease and network models on residual symptomology are highly heterogeneous. This can be attributed to the variety of treatments investigated to-date and the lack of content overlap amongst depression severity measures used. For example, research shows that, across seven commonly used clinician-rated [e.g., Hamilton Rating Scale for Depression, HRSD (41)] or self-reported measures [e.g., Beck Depression Inventory, BDI (42)], 52 disparate residual symptoms could be identified (23). Moreover, few studies solely examine residual symptoms, and only one individual patient data (IPD) network analysis sought to investigate specific residual symptoms following CBT and ADM treatment (43). Here, “depressed mood,” “feelings of guilt,” “suicidal thoughts,” “anxiety” and “general somatic symptoms” showed larger improvements following ADM's, compared to CBT. However, this IPD solely included randomized controlled trials (RCT's) of direct comparisons using only the HDRS. Restraining reviews to direct comparisons of one symptom severity measure limits the generalization of conclusions for either treatment.

To date, no systematic review combined with an IPD network analysis has investigated specific residual symptoms post-treatment. The benefits of conducting this type of study are 4-fold. Firstly, in comparison to traditional meta-analytic techniques, performed with aggregated data, IPD enables item-level symptom data to be obtained (44). By securing IPD samples, psychometric network models can be estimated to further disentangle residual symptom co-occurrences post-treatment (1). Secondly, identifying exactly which residual symptoms are central post-CBT and ADM's, may enable these treatments to be altered/extended to specifically target these residual symptoms (45). Thirdly, by investigating both CBT and AMD's, differentiations may be identified between actual residual symptoms and potential ADM's side effects (16, 45). Finally, novel relapse preventions strategies will be best informed for targeting those residual symptoms of high centrality (46).

Consequently, the current research aims to: (a) systematically review the literature on residual depressive symptomology following treatment with CBT alone, three new-generation ADM's (Escitalopram, Venlafaxine and Mirtazapine) alone and combined treatment (CBT + new-generation ADM's); and (b) estimate and compare the symptom network structure of residual depressive symptoms following CBT, ADM, and combined treatment using an individual patient data network analysis.

## Materials and Methods

PRISMA (47) guidelines for conducting and reporting systematic reviews were followed. A protocol was prospectively registered on PROSPERO (CRD42020141663).

### Search Strategy

Electronic databases, PsycINFO, PsycArticles, and PubMed were searched from the year 1980 to 13th May 2019. The search was updated on the 15th of October 2020. The year 1980 was chosen to align with current conceptualisations of an MDD diagnosis (48). For each database the following search string was used: (Depression OR MDD OR Major Depress^*^ Disorder OR Major Depression) AND (CBT OR Cognitive Behavio^*^ Therapy OR Cognitive Therapy) OR (Mirtazapine OR Escitalopram OR Venlafaxine OR new^*^ generation Antidepressants OR new^*^ generation ADM). All studies, using original data and published in English were included. Case reports, pilot studies and case series, were excluded.

### Selection Criteria

The inclusion criteria for the systematic review were:

1. A sample clinically diagnosed with MDD according to standardized measures e.g., DSM-III, DSM-III-R, DSM-IV, DSM-IV-TR, DSM-5 (48), or ICD-10 (49). Patients may have some comorbidities provided MDD was the primary diagnosis; substance use disorders, neurological disorders (e.g., dementia, Parkinson's) and physical illness's (e.g., chronic pain) were excluded.
2. Received either: individual face-to-face cognitive behavioral therapy (CBT) alone, escitalopram, mirtazapine or venlafaxine alone; or combined treatment (CBT + new-generation ADM's) as the only acute phase treatment for depression.
  a. CBT, for the purpose of this review, only included individual face-to-face formats, as the most effective method of delivery for depression (50). Online CBT was excluded, given its high heterogeneity in terms of format, content, and therapist support levels (51).
  b. New-generation ADM's were limited to mirtazapine, escitalopram, and venlafaxine due to their superior efficacy and higher prescription rate over other ADM's for the treatment of depression (36, 37).
3. Depression severity must be measured quantitatively post-treatment using standardized, validated, clinician-rated or self-reported measures of depressive symptoms severity.
4. For articles meeting the above criteria, corresponding authors were contacted for IPD. Then, the following inclusion criterion was applied for the IPD network analysis:
5. Provided post-treatment item-level symptom reports from the aforementioned depression severity measures.

### Data Extraction and Recorded Variables

Titles and abstracts returned from each database were directly imported to Endnote X9 reference managing software. First level initially required all duplicate articles to be deleted. Two authors independently classified titles and abstracts of remaining articles as “included” or “excluded” against the selection criteria. For this, author AW initially screened the database, authors AL, CB, and LC conducted independent screening. Discrepancies were resolved through consensus discussion with a third author (MS). Full texts of remaining potentially eligible articles were located for second level screening. Where a full text was not accessible, the corresponding author was contacted for access. All full texts were then independently screened against selection criteria by AW and then independently by CL, EL and NS and sorted into either “yes” for systematic review or “no” alongside a reason for exclusion. All authors of articles meeting the systematic review criteria were contacted to provide IPD. Authors who did not respond initially were contacted once more. All data-sets not obtained following two attempts of contact were then excluded from the analysis under “data not available.”

From each of the studies meeting the systematic review inclusion criteria, both those “providing IPD,” and “data not available,” the following variables were independently coded: (1) participants characteristics, including: number of participants at each assessment time point, mean (*M*) age and percentage of males; (2) intervention characteristics, including type of intervention administered; and (3) symptom characteristics, including: symptom measures used.

From the studies “providing IPD,” all original data-sets obtained were checked to see if the data received matched the data reported in the publication. The study sample size, percentage of males and mean age were calculated from the dataset received and checked against the published article. Any discrepancies were resolved with the corresponding author. Item-level post-treatment scores were also checked for invalid or out-of-range item scores (e.g., BD-II item scores >3; MADRS item score >6). In addition, to assess risk of bias within individual studies that provided the IPD, the “Evidence Project” tool was applied (52).

### Statistical Analyses

Prior to estimating residual depressive symptom networks, Mann-Whitney U tests assessed if mean age and gender distribution were comparable across samples “providing IPD” or “data not available.” IPD samples were then firstly pooled by symptom severity measure (e.g., BDI, HRSD) and secondly by treatment type (e.g., CBT, ADM). Within each symptom severity measure, the IPD demographics across treatment type were then compared by Chi-Square Test of Association for gender and Mann-Whitney U Test for age.

#### Network Estimation

At the registration level, it was planned to fit an Ising model to the data [CRD42020141663]. However, since, it was evaluated that fitting a Gaussian Graphical Model (GGM) is better as (a) we refrained from combining symptoms from different scales due to lack of content overlap, thus dichotomization became unnecessary (23); (b) we expected to obtain more original data, thus using full response scales and the GGM would attain greater power (53); and (c) using binary data with different sample sizes for the Network Comparison Test (outlined below) further results in low power (54). Therefore, residual symptom network estimations were conducted by fitting the GGM to the data for each symptom severity measure, within each treatment type. The GGM is an undirected network of partial correlation coefficients, in which edges represent conditional independence among nodes [residual symptoms (55)]. Due to the GGM estimating a large number of parameters, it is likely some false positive edges (relationships between residual symptoms) were obtained. To control for these Type-I errors and estimate a sparser, interpretable model, network regularization techniques were also conducted. Specifically, network regularization was conduct in “bootnet” which automatically combines a LASSO regularization algorithm with the extended Bayesian Information Criterion (EBIC) model selection (56). First, the graphical LASSO algorithm was used to shrink edges of the network and set small edges to zero (57). Second, the EBIC was used to estimate 100 different network models with different degrees of sparsity (58). The model with the lowest EBIC was selected given a certain value on the hyperparameter (γ), to control for a trade-off between including false positive edges and removing true edges. The starting value of γ was set at 0.0 for this study to err on the side of discovery, as opposed to erring on the side of caution (59). Given the number of edges identified with γ, non-parametric bootstrapped confidence intervals (*CI*'s) were calculated to estimate edge weights variability. Wide bootstrapped *CI*'s indicated that edge strength should be interpreted with caution; narrow bootstrapped *CI'*s indicated that edge strength could be interpreted with confidence (59). Residual depressive symptom networks were plotted using an averaged layout to aid interpretation when comparing across treatments.

#### Centrality

All constructed networks were then further analyzed by investigating the centrality (importance) of each residual symptom (node) in the network. The centrality metrics of strength and expected influence are reported in the main text (27). Both “strength” and “expected influence” measure the number and strength of connections among residual symptoms post-treatment. However, “expected influence,” unlike “strength,” accounts for both positive and negative edges and thus outperforms “strength” when negative edges are present (27). The correlational stability coefficient (CS-coefficient) was also calculated to gain insight into the stability of centrality measures. CS-coefficient's represents the maximum proportion of cases that can be dropped, such that with 95% probability, the correlation between original centrality indices and those of random subsets of the data is 0.7 or greater (59). The value of 0.7 was chosen as this value is interpreted as indicating a large effect in psychological sciences (60). Information on the centrality metrics “betweenness,” and “closeness” can be found in the Supplementary Materials. These metrics were not included in the main text due to recent evidence expressing concern on the accuracy of these measures when applied to psychological networks (61).

#### Network Comparison Test

The residual symptom networks post-CBT and ADM's for each symptom severity measure were directly tested for differences with respect to invariance of the following indices: structure-does the structure of residual symptoms differ as a whole across treatment type; global strength- does the level of connectivity of residual symptom networks differ across treatment type; centrality- do central residual symptoms in the networks differ across treatment type; edges- do co-activations/associations between specific edges in the residual symptom networks differ across treatment type. These tests were carried out using the Network Comparison Test (NCT), implemented using the R-package “NCT.” The NCT is a two-tailed permutation test in which the difference between two groups is calculated repeatedly for randomly re-grouped individuals (54). Number of permutations was set at 5,000 and multiple testing of edges/nodes within NCT's was controlled for using the Holm (62) sequentially rejective multiple hypothesis correction.

## Results

**Figure 1:**
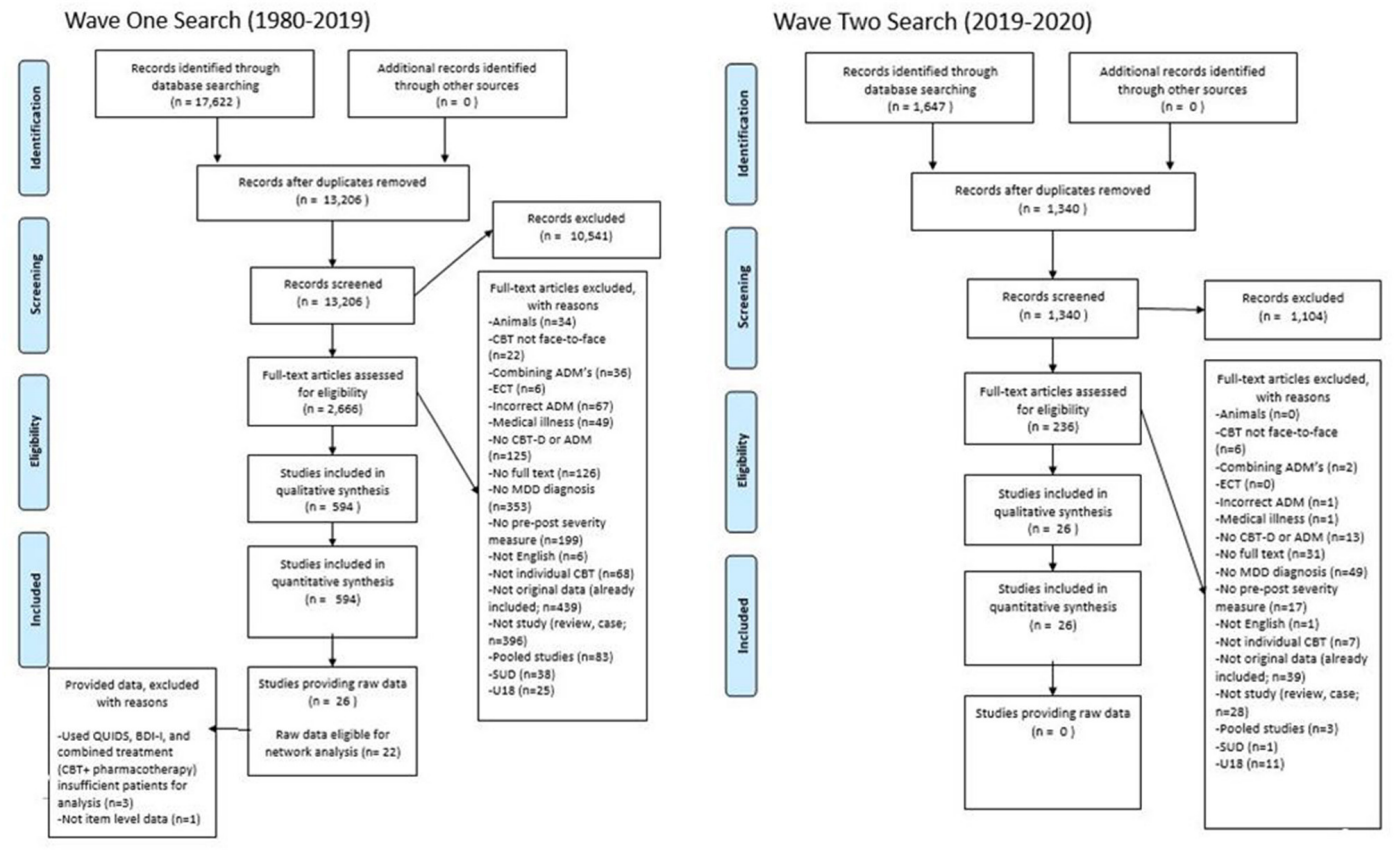
PRISMA Flowchart of the review process and criteria for study selection.

After deleting duplications, the search strategy identified 14,546 citations from which 2,902 were assessed for eligibility, 620 met the inclusion criteria, 25 provided item-level symptom data and 22 were included in the network analysis. See Figure 1 for a flow chart of the review process.

From the 620 eligible studies (*n*), 663 samples (*k*) were extracted. A total of 88,305 patients (*N*), were treated with either CBT *N* = 11,059, ADM's *N* = 77,022 (Mirtazapine, *N* = 14,280; Escitalopram, *N* = 41,980; Venlafaxine, *N* = 20,759) or combined treatment (CBT + ADM's) *N* = 227. The samples had a mean age of 43.33 (range 24.01–74.7; *M*_*CBT*_= 38.70, $M_{ADM^{\prime }s}$ = 44.78, *M*_*combined*_ = 43.54). On average, 34.91% of the participants were male (*M*_*CBT*_= 34.77%, $M_{ADM^{\prime }s}$= 34.92%, *M*_*combined*_ = 43.38%). Regarding post-treatment symptom severity measures, the most commonly administered included two clinician-rated measures- Hamilton Rating Scale for Depression-17 (HRSD-17, *k* = 461) and Montgomery-Asberg Depression Rating Scale (MADRS, *k* = 180), and one self-reported measure- the Beck Depression Inventory version I and II (BDI, *k* = 102; BDI-II, *k* = 59). During the data request process, 335 studies did not respond to two emails, 138 responded they could not provide the data (Supplementary Materials 1), 121 had either incorrect or no contact information available and 25 provided item-level data. Following inter-rater reliability checks 22 studies were brought forward for network analysis. Of those whom provided item level-data but were not included in the final analysis due to not obtaining enough IPD to estimate residual symptom networks, two studies (63, 64) used different symptom severity measures, and one (65) study used a combined treatment approach (CBT + pharmacotherapy). See Supplementary Materials 20 for full details on extracted data from eligible samples.

From the 22 studies included in the network analysis, 25 samples were extracted (*k*_*CBT*_ = 11, *k*_*ADM*_ = 14). A total of 1,389 patients were included in the network analyses (*N*_*CBT*_ = 467, $N_{ADM^{\prime }s}$ = 922). The samples had a mean age of 44.54 (range 24.01–74.7; *M*_*CBT*_ = 38.91, $M_{ADM^{\prime }s}$ = 48.96). On average, 35.48 % of the participants were male (*M*_*CBT*_ = 30.71, $M_{ADM^{\prime }s}$ = 39.22). No significant differences were observed when comparing samples “included” vs. “excluded” for network analysis across the distribution of gender (*U* = 7170, *p* = 0.960) and age (*U* = 7052, *p* = 0.784). Using this item-level data, residual symptom networks were estimated within each symptom severity measure. Sufficient data was provided to estimate networks for one self-reported severity measure- BDI-II (*k* = 9), and two clinician-rated measures- HRSD (*k* = 14) and MADRS (*k* = 6). See Table 1 for details on the network-analyzed studies and Supplementary Materials 21 for a risk of bias assessment of network-analyzed studies.

**Table 1:** Study characteristics of the network analyzed samples.

**Study name**	**Year**	***N* (Post-treatment)**	**Mean age**	**Gender (*N* Male)**	**Intervention**	**Symptom measure(s)**
Altenstein-Yamanaka	2017	63	39.65	29	CBT	BDI-II
Azvedo da Silva	2017	30	24.07	8	CBT	HRSD-17
Basu	2017	87	35.12	73	Escitalopram	MADRS
Bernecker	2016	32	45.58	10	CBT	HRSD-17
Carter	2013	37	39.11	13	CBT	BDI-II (*N =* 35), HRSD-17 (*N =* 36), MADRS (*N =* 37)
Ciusani	2004	10	38.6	3	Venlafaxine	MADRS
Forman	2007	74	29.96	10	CBT	BDI-II
Groves	2015	19	34.95	9	CBT	MADRS
Halaris	2015	19	38.74	5	Escitalopram	HRSD-17
Heller	2013	12	29.67	7	Venlafaxine	HRSD-17
Huang	2016	48	N/A	N/A	Escitalopram (*N =* 30), Mirtazapine (*N =* 8), Venlafaxine (*N =* 10)	BDI-II
Lenze (IRL-GREY)	2015	392	69.01	141	Venlafaxine	MADRS
Lopes	2014	16	35.56	5	CBT	BDI-II
Luty	2007	71	36	22	CBT	BDI-II, HRSD-17, MADRS
Myung	2012	36	66	7	Mirtazapine (*N =* 29), Venlafaxine (*N =* 7)	HRSD-17
Nakagawa	2017	39	39.53	25	CBT	BDI-II, HRSD-17
Saghafi	2007	171	73.02	53	Escitalopram	HRSD-17
Sefarty	2009	64	74.22	11	CBT	BDI-II
Sirot	2012	31	49	13	Mirtazapine	HRSD-17
Soczysnka	2014	17	42	9	Escitalopram	HRSD-17
Eddington	2015	22	N/A	N/A	CBT	BDI-II
Osvath	2007	99	42.26	42	Mirtazapine	HRSD-17

*N/A, not available*.

### Beck Depression Inventory-II (BDI-II)

#### Sample Characteristics

Eight out of 48 eligible samples provided item-level BDI-II data post-CBT. A total of 376 patients (*N*) were included for network analysis. Studies providing data *Ns* ranged from 16 to 74, with a mean age of 42.31 [standard deviation (*SD*) = 18.19]. On average, 34.33% of the patients were male (*SD* = 10.87). Comparing samples whom “provided IPD” vs. “data not available” post-CBT, no significant differences were observed in the distributions of gender (*U* = 116, *p* = 0.234) or age (*U* = 134.5, *p* = 0.694). Regarding ADM's, only one out of eight eligible studies provided item-level BDI-II data post-ADM's. Therefore, an insufficient number of patients (*N* = 48) were obtained to construct, estimate and compare this residual symptom network.

#### Network Estimation

**Figure 2:**
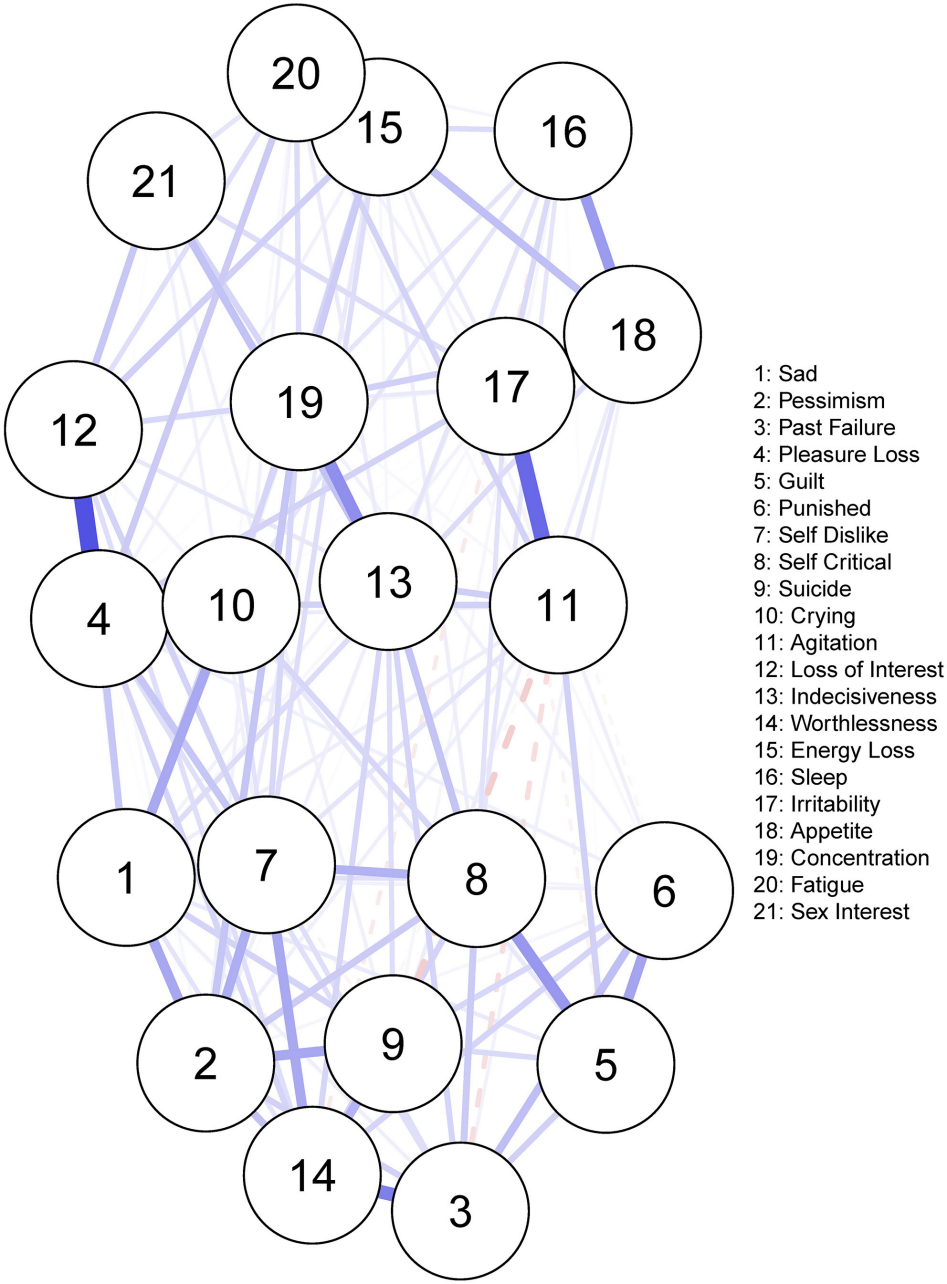
Residual symptom network post-CBT, measured using the BDI-II. Blue edges indicate symptom activation, and red edges indicate symptom inhibition.

Figure 2 displays the residual symptom network of the 8 CBT studies. *Energy loss* had the highest strength centrality included (Supplementary Materials 2). The centrality stability (CS) coefficient was relatively strong, suggesting 67.3% of the sample could be dropped before bootstrapped correlations with original centrality values dropped below 0.7 (Supplementary Materials 3). Strongest edges were between; *loss of interest—loss of pleasure* (*r* = 0.35), *irritability—agitation (r* = *0.30)*, and *fatigue—energy loss (r* = *0.51)*. Bootstrapped confidence intervals for all edge weights were moderate in size, indicating that interpreting the order of the edges in this residual symptom network should be done with some care (Supplementary Materials 4 and 5).

### Hamilton Rating Scale for Depression-17 (HRSD-17)

#### Sample Characteristics

Five out of 74 eligible samples provided item-level HRSD data post-CBT (*N* = 208) and eight out of 387 eligible samples provided item-level HRSD data post-ADM (*N* = 385). Comparing the demographics of CBT and ADM residual symptom networks, a significant difference was observed in age, with the ADM network (*M* = 57.99, *SD* = 18.19) having a significantly older sample than the CBT network (*M* = 36.89, *SD* = 11.51; *U* = 14380, *p* < 0.001). No significant difference was observed in the distribution of gender across the residual symptom networks, χ2 (1) = 0.521, *p* = 0.470, with 35.58% of the CBT and 32.64% of the ADM network comprising of males. Furthermore, no significant differences were observed in the distributions of gender (CBT, *U* = 121.50, *p* = 0.561; ADM, *U* = 1,514.00, *p* = 0.672) or age (CBT, *U* = 122.00, *p* = 0.479; ADM, *U* = 1673.50, *p* = 0.379) when comparing samples whom “provided IPD” vs. “data not available” post-treatment. These characteristics are important to consider when drawing inferences from the residual symptom network structures.

#### Network Estimation

**Figure 3:**
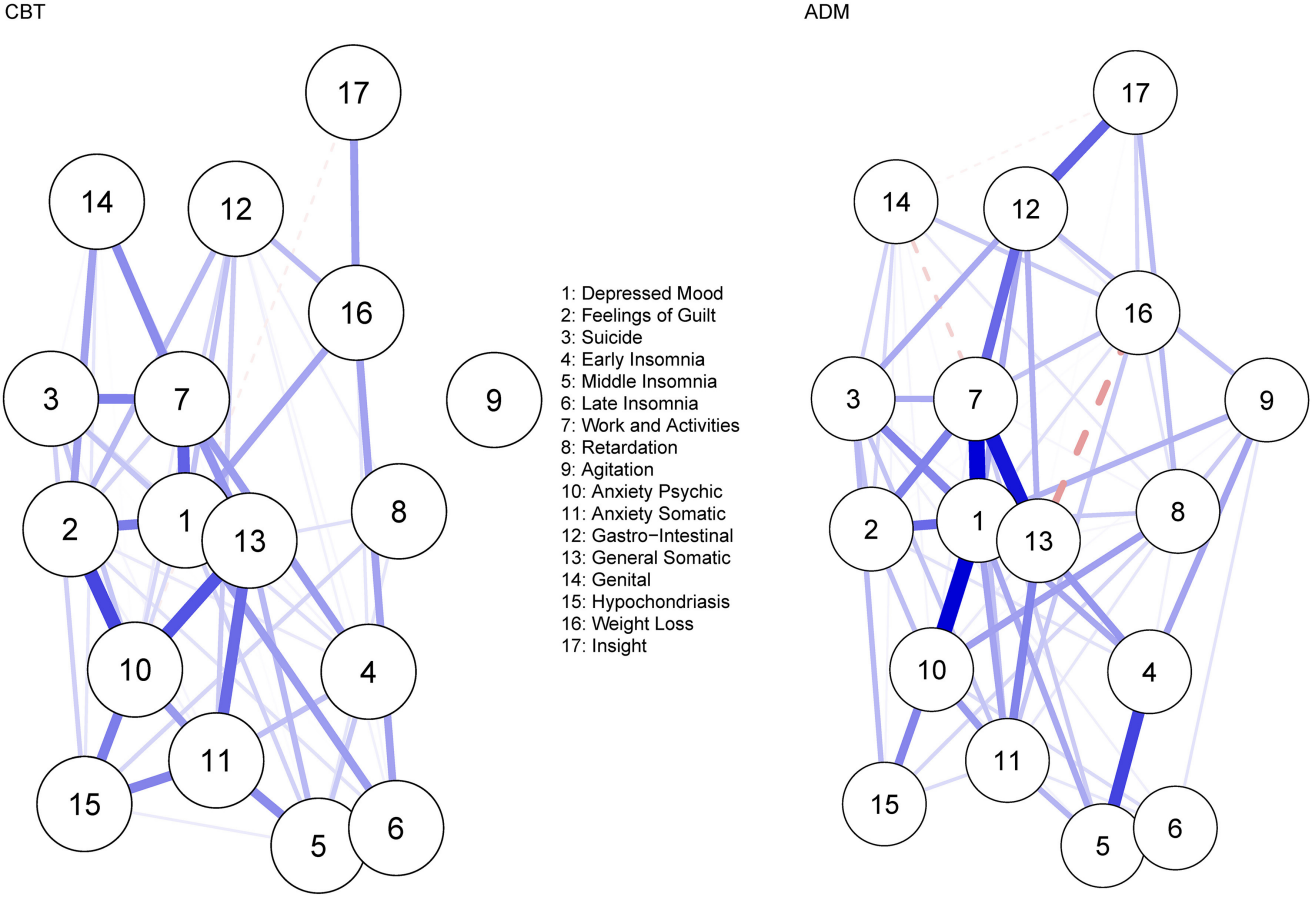
Residual symptom network post-CBT and ADM's, measured using the HDRS-17. Blue edges indicate symptom activation and red edges indicate symptom inhibition.

Figure 3 displays the residual symptom networks of the 5 CBT and 9 ADM samples. Across both networks, residual symptoms with the highest strength centrality included; *depressed mood* followed by *impairment in work and activities*. Post-CBT, *anxiety psychic* and *general somatic* were also central (Supplementary Materials 6). Regarding the CBT network, the CS coefficient was moderate, suggesting 51.4% of the sample could be dropped before bootstrapped correlations with original centrality values dropped below 0.7 (Supplementary Materials 7). For the ADM, network CS coefficient was strong, suggesting 75.1% of the sample could be dropped before bootstrapped correlations with original centrality values dropped below 0.7 (Supplementary Materials 8). Strongest edges amongst residual symptoms were between *depressed mood—impairment in work and activities* for both treatments (CBT, *r* = 0.19; ADM, *r* = 0.28); *late insomnia—middle insomnia* (*r* = 0.31), *feelings of guilt—anxiety psychic* (*r* = 0.23) and *general somatic—anxiety psychic* (*r* = 0.21) post-CBT; and *general somatic—impairment in work and activities* (*r* = 0.26) and *depressed mood—anxiety psychic* (*r* = 0.21) post-ADM's. Bootstrapped confidence intervals for all edge weights were again relatively large, indicating that interpreting the order of the edges in this residual symptom network should be done with care (Supplementary Materials 9–12).

#### Network Comparison

No significant difference was observed when comparing the overall structure (*p* = 0.087) of the residual symptom networks post-CBT and ADM treatment. However, looking at specific network structures, *depressed mood* was a significantly *(p* < 0.001) more central residual symptom post-ADM treatment (vs. CBT), even after multiple testing corrections (*p* = 0.007). No significant difference was observed when comparing the global strength of the residual symptom networks (*p* = 0.416).

### Montgomery-Asberg Depression Rating Scale (MADRS)

#### Sample Characteristics

Three out of 7 eligible samples provided item-level MADRS data post-CBT (*N* = 127) and three out of 169 eligible samples provided item-level MADRS data post-ADM's (*N* = 489). Comparing demographics of the residual symptom networks, a significant difference was observed in age, with the ADM network (*M* = 62.36, *SD* = 15.50) having a significantly older sample than the CBT network (*M* = 36.91, *SD* = 11.09; *U* = 6786.5, *p* < 0.001). No significant difference was observed in the distribution of gender across the residual symptom networks, χ*2* (1) = 1.16, *p* = 0.282, with 37.6% of the CBT and 39.06% of the ADM network comprising of males. Furthermore, no significant differences were observed in the distributions of gender (CBT, *U* = 6, *p* = 0.700; ADM, *U* = 342.00, *p* = 0.143) or age (CBT, *U* = 10.00, *p* = 0.229; ADM, *U* = 187.0, *p* = 0.617) when comparing samples whom “provided IPD” vs. “data not available.” Again, these characteristics are important to consider when drawing inferences from the residual symptom network structures.

#### Network Estimation

**Figure 4:**
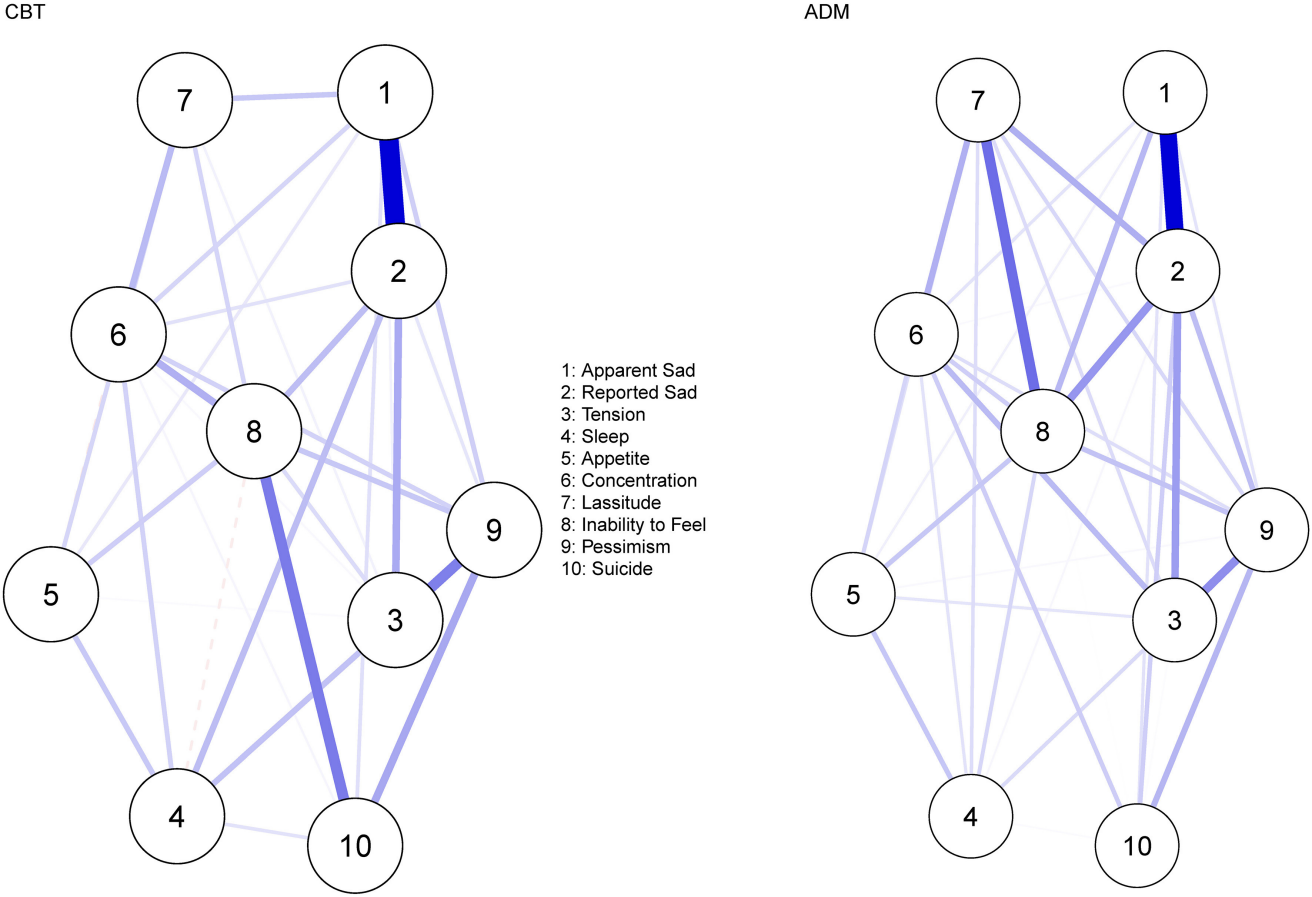
Residual symptom network post-CBT and ADM's, measured using the MADRS. Blue edges indicate symptom activation and red edges indicate symptom inhibition.

Figure 4 displays the residual symptom networks of the 3 CBT and ADM samples. Across both networks, residual symptoms with the highest strength centrality included; *reported sadness, apparent sadness* and *inability to feel* (Supplementary Materials 13). In the CBT network, the centrality stability (CS) coefficient was average, suggesting 36.2% of the sample could be dropped before bootstrapped correlations with original centrality values dropped below 0.7 (Supplementary Materials 14). For the ADM network, the CS coefficient was strong, suggesting 75.1% of the sample could be dropped before bootstrapped correlations with original centrality values dropped below 0.7 (Supplementary Materials 15). Across both networks, strongest edges amongst residual symptoms were between; *apparent sadness—reported sadness* (CBT, *r* = 0.60; ADM, *r* = 0.53) and *tension—pessimism* (CBT, *r* = 0.30; ADM, *r* = 0.24). Furthermore, for the CBT network a strong edge was also demonstrated between *suicide—inability to feel* (*r* = 0.31) and for the ADM network between *lassitude—inability to feel* (*r* = 0.30). Bootstrapped confidence intervals for all edge weights were relatively large, indicating that interpreting the order of the edges in the residual symptom networks should be done with care (Supplementary Materials 16–19).

#### Network Comparison

A significant difference was observed in the overall structure of the residual symptom networks post-CBT and ADM treatment (*p* = 0.018). Specifically, *lassitude*, although not highly central overall, was significantly more central post-ADM's (vs. CBT; *p* = 0.011). Concerning specific edge-invariance, co-occurrences amongst *inability to feel—suicide* were significantly stronger post-CBT (*r* = 0.31) in comparison to ADM treatment (*r* = 0.00; *p* < 0.001), even with multiple testing corrections (*p* = 0.009). Co-occurrences amongst *inability to feel—lassitude* were significantly stronger post-ADM's (*r* = 0.30) in comparison to CBT (*r* = 0.09, *p* = 0.042). Although, co-occurrences amongst *reported sad—sleep* (*p* = 0.024), and *apparent sad—lassitude* (*r* = 0.049) were also significantly more central post-CBT (vs. ADM's), overall edge strength remained low, thus these findings should be interpreted with caution. No significant difference was observed in the global strength of the networks (*p* = 0.733).

### Moderated Network Analyses and Topological Overlap (*post-hoc*)

Descriptive covariates (age and gender) were independently entered as moderators into all aforementioned network models (66). Descriptive covariates did not significantly moderate any of the previously reported residual symptom co-occurrences for CBT and ADM networks across BDI-II, HDRS, and MADRS measures.

The goldbricker algorithm (67) was also applied to all networks to identify potential problematic topological overlap amongst network items. Topological overlap was not identified amongst any of the previously reported central residual-symptoms or their co-occurrences. Full results of these analyses can be found in the Supplementary Materials 22.

## Discussion

The current systematic review and IPD network analysis evaluated residual depressive symptomology following CBT and ADM's across three validated measures. For HRSD networks, central residual symptoms included *impairment in work and activities* and *depressed mood* following both treatments; and *anxiety* and *general somatic* symptoms post-CBT. No significant differences were observed in the overall structure or global strength of residual HRSD symptom networks across treatment type. However, *depressed mood* was significantly more central post-ADM's than following CBT. Regarding the MADRS networks, *reported sad, apparent sad* and *inability to feel* were central post-CBT and ADM's. A significant difference was observed in the overall structure of residual MADRS symptom networks. Specifically, *lassitude* was significantly more central post-ADM's (vs. CBT) and significantly stronger symptom co-occurrences were observed amongst “*inability to feel-suicide*” post-CBT (vs. ADM's), and “*inability to feel-lassitude*” post-ADM's (vs. CBT). No significant difference was observed in the global strength of MADRS networks. For the BDI-II, *energy loss* was the most central self-reported residual symptom post-CBT.

*Depressed mood* and anhedonia- indirectly captured by *impairment in work and activities* (HRSD) and *inability to feel* (MADRS), were the most central and strongly connected residual symptoms following CBT and ADM's across both clinician-rated measures. The same centrality was not observed across the self-reported BDI-II samples. This centrality difference across measures may relate to the lower tendency for patients to self-report mood symptoms, with cross-measure consensus favoring physical symptoms [e.g., fatigue (68)]. Nevertheless, the higher centrality and co-occurrence of these symptoms was not surprising given both, by DSM definition, are core MDD criteria (48). This result is also consistent with previous psychometric network models and prevalence statistics reporting up to 96% of patients experience these symptoms post-treatment (19, 29, 30, 38, 69, 70).

Regarding *depressed mood*, explanations for post-treatment centrality often relate to its pre-treatment influence. Research shows that, although the strength of *depressed mood* symptom's connections change throughout treatment, the overall centrality of the symptom remains for the most part unchanged (34, 70). In relation to residual anhedonia, explanations possibly lay with the treatments' focus. Indeed, CBT primarily focuses on repairing negative cognitions and reducing depressed mood, both negative valanced. Resultantly, the positive features of well-being and feeling pleasure in some instances are potentially left to prevail post-treatment (69). With ADM's, anhedonia has also shown to be resistant to certain first-line pharmacotherapies, including escitalopram and venlafaxine- making up over half of the current samples (71). Identifying these symptoms post-treatment is important as depressed mood shows strong co-occurrences with *anxiety* (HRSD) post-ADM's. Anhedonia also shows strong co-occurrences with *general somatic* symptoms (HRSD) and *lassitude* (MADRS) post-ADM's, and *suicide* related symptoms (MADRS) post-CBT. Therefore, if left to prevail or persist, *depressed mood* and anhedonia are likely to not only sustain network activation, but increase spread of network connectivity and thus increase risk of future relapse/recurrence (46). Strategies developed to specifically target these symptoms and their co-occurrences, such as augmented depression therapy (ADepT), could be considered, perhaps as part of a suite of stepped-care residual symptom interventions (69).

Furthermore, although *depressed mood* was highly central across both treatments, the NCT also showed this symptom was significantly more central post-ADM's (vs. CBT) within the HRSD samples. The delayed antidepressant effect of ADM's might explain this prominence. Specifically, whilst both CBT and ADM's target mood systems similarly, ADM's do not directly enhance mood but instead change the relative balance of negative to positive emotional processing (17). Thus, ADM's may be slower than CBT to target *depressed mood* in the acute phase of treatment. However, as this result was only observed with one clinician-rated scale, further research needs to evaluate this hypothesis.

Fatigue–indirectly captured by *general somatic* (HRSD) and *energy loss* (BDI-II) was also identified as a central symptom, but only within CBT networks. Importantly, an ADM network could not be estimated for BDI-II samples, making cross-treatment comparisons of this measure not possible. Nevertheless, the HRSD-related result expands on previous psychometric network models, observing fatigue-related symptoms as consistently central post-treatment (1, 26, 28). The centrality of *general somatic* symptoms post-CBT and not ADM's may result from the superior ADM's effect on inflammatory cytokines. Depression is associated with a greater release of pro-inflammatory cytokines, which often lead to fatigue (72). The clinical efficacy of SSRI's has been also linked to their anti-inflammatory effects, reducing fatigue-related symptoms by almost 40% (73), and escitalopram, an SSRI, makes up for over 70% of our HRSD samples. Identifying fatigue-related symptoms as central post-CBT is important as these symptoms also show strong co-occurrences with *anxiety* (HRSD), *impairment in work and activities* (HRSD), and *fatigue* (BDI-II). Thus, whilst pharmacological agents targeting inflammatory markers, may prove optimal by directly targeting fatigue, CBT interventions focused on reducing the co-occurrences amongst fatigue-related symptoms could also be an option to reduce the spread of network connectivity (45).

*Anxiety* was also identified as a central residual symptoms post-CBT, within HRSD samples. It is important to note, neither the MADRS or BDI-II measure anxiety complaints directly, making cross measure comparisons of these symptoms not possible (23). However, this result does align with another IPD showing, CBT may be less effective at treating anxiety symptoms (43). This may be attributed to disorder comorbidities, disorder specific interventions, and/or designs of included studies. Around 45–67% of individuals with MDD display comorbid anxiety symptoms and/or disorders (74). Unlike ADM's which are also considered first-line, effective treatments for anxiety, CBT's efficacy is often disorder specific- treating a single disorder or diagnosis (75). Resultantly, CBT here focused primarily on depression, potentially leaving anxiety symptoms for the most part unchanged. Alternatively, it is also important to note, upon closer inspection of included samples, over half of ADM samples excluded patients with comorbidities. This reflects strict inclusion criteria adopted in RCT's of ADM's (76). Whilst this is not a true representation of MDD in the real-world, potentially excluding 50% of patients, it may also explain why anxiety symptoms were not observed as central-post-ADM treatment, with individuals displaying these symptoms screened out pre-treatment. Even so, identifying residual anxiety post-CBT is important as it supports the shift toward transdiagnostic CBT protocols designed to focus on treating specific symptoms and their co-occurrences over syndromes (74, 75). Indeed, for those presenting with severe anxiety symptoms, ADM's, particularly SSRI's, may be an optimal acute treatment choice in line with their efficacy and tolerability (77).

In sum, major differences in residual symptom networks across treatment type were not observed, with core MDD symptoms- depressed mood and anhedonia central and common to all. Furthermore, NCT's showed no significant difference in overall network structure within-HRSD samples, nor were differences observed in global strength within-HRSD and MADRS samples. This equivalence in global strength may indicate residual symptom severity was equal across treatment type, supporting longstanding literature on overall equal effectiveness of depression treatments (78). However, within-MADRS residual symptom networks, significant structural differences were observed across treatment type. Specifically, *lassitude*, although not highly central in general, was significantly more central post-ADM's (vs. CBT) and significantly stronger symptom co-occurrences were observed for “*inability to feel-suicide*' post-CBT (vs. ADM's), and “*inability to feel–lassitude*” post-ADM's (vs. CBT). Regarding co-occurrences amongst “*inability to feel-suicide*” post-CBT, this result is consistent with previous research showing not only is anhedonia a significant risk factor for suicide, but CBT (vs. ADM's) is less effective in treating suicide ideation (79). However, research does suggest this may be due to the therapeutic process and alliance associated with CBT (vs. ADM's), resulting in patients feeling more comfortable disclosing suicidal ideation than they would throughout ADM treatment (79). Nevertheless, for CBT, close attention should always be accorded to suicidal thoughts. Identification of these thoughts may warrant a switch or combination of CBT+AMD's, even before completion of acute-phase treatment (80).

### Limitations and Future Recommendations

Only inter-relations of residual symptoms could be observed as networks are based on cross-sectional data, with findings being thus exploratory in nature. Assumptions cannot be made on the directionality of residual symptom activation or their interactions over time (55). Temporal dynamics of these residual symptoms following CBT and ADM's across should be examined by future research. Inherent to our method, as IPD's are not originally collected for network analyses, individual items and measures cannot provide a complete picture of residual symptoms across CBT and ADM's (25).

Additionally, included symptom severity measures lack content overlap. Although many depressive symptoms can be indirectly captured across scales (e.g., *impairment in work and activities*- HRSD and *inability to feel*- MADRS), the heterogeneity of scales' items makes cross-measure comparisons difficult (23), highlighting the need for future replication. The estimated network models should also not be interpreted as a theory on the residual symptoms' relationships (81). Our analyses/explanations are purely data-driven and do not uncover theoretical processes [e.g., symptom feedback loops (82)]. Nevertheless, the exploratory work conducted here provides a basis for hypothesis-generating work on residual symptoms following different treatments. For example, examining whether ADM's are superior to CBT in specifically treating anxiety and somatic complaints in MDD.

Finally, we expected to obtain more raw data from eligible samples. Moreover, we also missed retrieving some IPD due to not offering authorship in exchange for data. Whilst only 5 authors requested authorship in exchange for IPD, in order to be fair to the registration process and to the many other researchers who did not ask for authorship, we did not follow on those offers. This lack of IPD limits the generalizability of the estimated networks and the scope of potential analyses. Although cases exist where data sharing may not be possible (e.g., confidential personal information), for the most part, authors ignore data requests. Lack of raw data is a non-negligible part of the causes of the reproducibility crisis. Importantly, this problem was not unique to the current study, previous research shows 97% of authors did not present raw data upon request for journal submissions (83). In highlighting this issue, we do not intend to “call-out” or “blame” authors who did not provide data. We are aware data sharing can sometimes be constrained by institutions, funding, and pharmaceutical agencies. However, if progress is to be made in depression research, evidence must be provided upon which claims are made (84). Therefore, while data-sharing policies should be implemented and encouraged from the top-down (e.g., institutions), authors should also make greater efforts in sharing data or at least in convincing why such a request is not possible. “No raw data, no science” (83).

## Conclusion

Our IPD network analysis has important clinical and research implications. First, clearly, residual depressive symptoms not only persist following treatment, but are also dynamic in their centrality and symptom co-occurrences. To move toward better understandings of residual symptomology, future research must not rely on unspecific summed-scores and acknowledge the true complexity of depression related symptoms. Second, residual depressed mood and residual anhedonia were central across both treatment types and both clinician-rated scales. These core depression symptoms are known to be associated with significant risks for future relapse and increased functional impairments. Thus, first-line treatments and/or relapse prevention strategies should aim to directly target these central symptom as this may have positive impacts on reducing the overall network connectivity. Furthermore, a distinguishing feature between treatments was the centrality of anxiety and fatigue-related symptoms post-CBT. This suggests for patients presenting with these primary complaints, CBT may not be a superior first-line treatment option. Finally, joint efforts from researchers and institutions making data openly available are needed to ensure essential progress in depression prevention research.

## Author Contributions

AW and MS contributed to conception and design of the study and wrote the first draft of the manuscript. AW initially screened the databases with AL, CB, CL, EL, LO'S, and NS conducting second screening/reliability checks. Screening discrepancies were discussed for consensus with MS. AW performed statistical analysis. All authors contributed to manuscript revisions, read, and approved the submitted version.

## Funding

AW has received funding from the Irish Research Council and Analog Devices International (EPSPG/2020/487) to conduct research on identifying indicators of depression relapse versus depression recovery while facing stress through vital signs monitoring.

## Conflict of Interest

The authors declare that the research was conducted in the absence of any commercial or financial relationships that could be construed as a potential conflict of interest.

## Publisher's Note

All claims expressed in this article are solely those of the authors and do not necessarily represent those of their affiliated organizations, or those of the publisher, the editors and the reviewers. Any product that may be evaluated in this article, or claim that may be made by its manufacturer, is not guaranteed or endorsed by the publisher.
