# Supplementary material for: A Systematic Review and Individual Patient Data Network Analysis of the Residual Symptom Structure Following Cognitive-Behavioral Therapy and Escitalopram, Mirtazapine and Venlafaxine for Depression
Source: Front Psychiatry. 2022 Feb 1;13:746678. doi: 10.3389/fpsyt.2022.746678 (PMC8843824; doi:10.3389/fpsyt.2022.746678)
Supplement: Supplementary file 1 [file Data_Sheet_1.docx]

**Supplementary Materials For:**

**A Systematic Review and Individual Patient Data Network Analysis of the Residual Symptom Structure following Cognitive-Behavioural Therapy and Escitalopram, Mirtazapine and Venlafaxine for Depression**

Aoife Whiston^1^, Amy Lennon^1^, Catherine Brown ^1^, Chloe Looney^1^, Eve Larkin^1^, Laurie O’Sullivan^1^, Nurcan Sik^1^, Maria Semkovska^2^

**Supplementary Materials 1.** Reasons provided by corresponding authors for not providing IPD

| **Reason** | **Responses (Number)** |
| --- | --- |
| Data belong to pharmaceutical company | 25 |
| Data sharing agreements/legal aspects | 9 |
| Ethics (e.g. not included in consent forms for data sharing) | 5 |
| Moved institution/retired | 15 |
| No access/no resources to provide | 29 |
| No item level stored | 17 |
| No reason given ('cannot provide') | 16 |
| Not original dataset | 1 |
| Study too old +5 years | 2 |
| Study too old 10+ years | 11 |
| Technical issues (e.g. computer crash) | 2 |
| Using data for similar project | 2 |
| Will provide in exchange for authorship | 5 |

*Note:* This table does not account for authors who simply ignored email responses.

**Supplementary Materials 2.** Residual symptom network centrality plot post-CBT, measured using the BDI-II
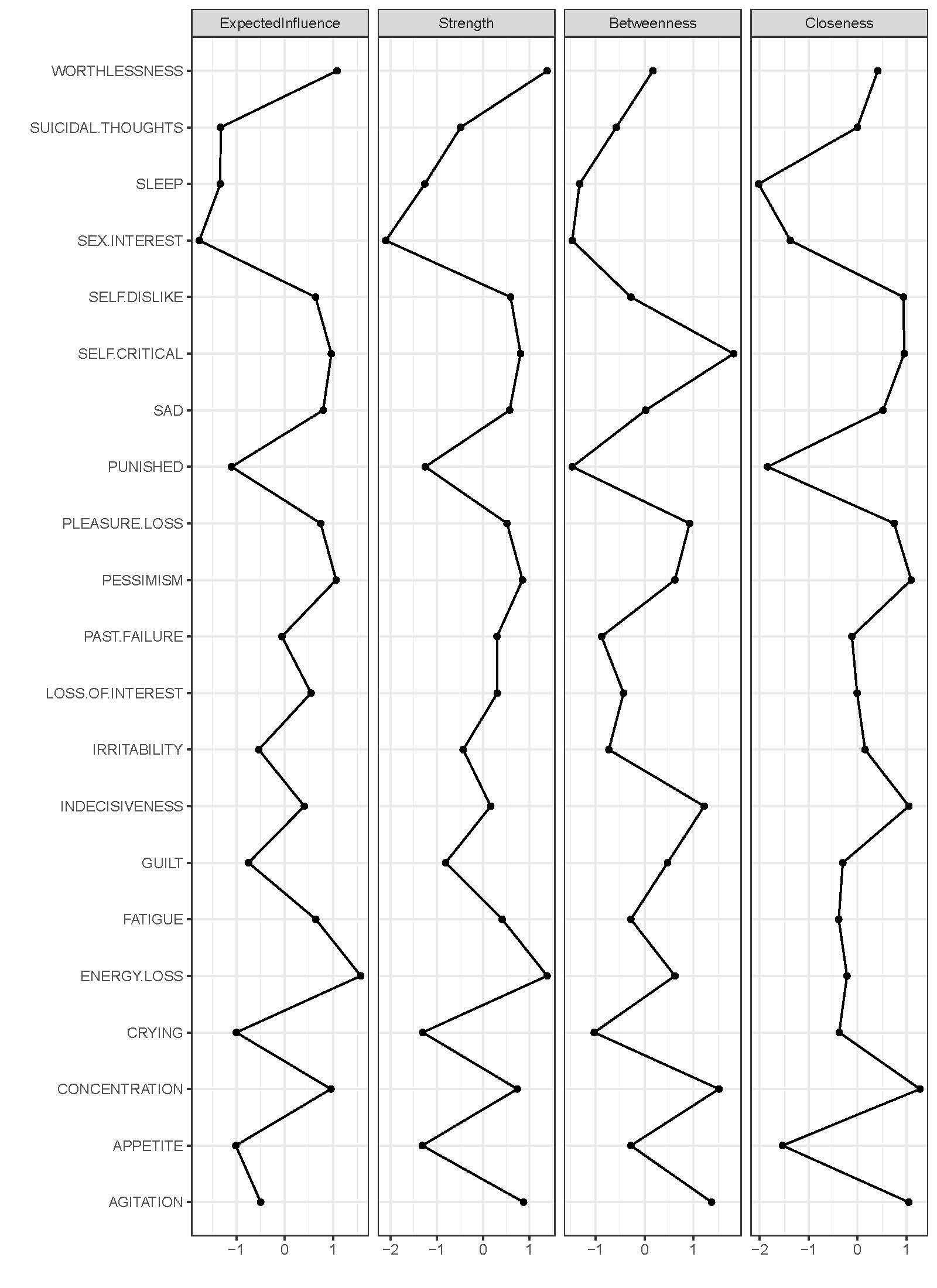


**Supplementary Materials 3.** Case drop bootstrap for the residual symptom network post-CBT measured using the BDI-II.


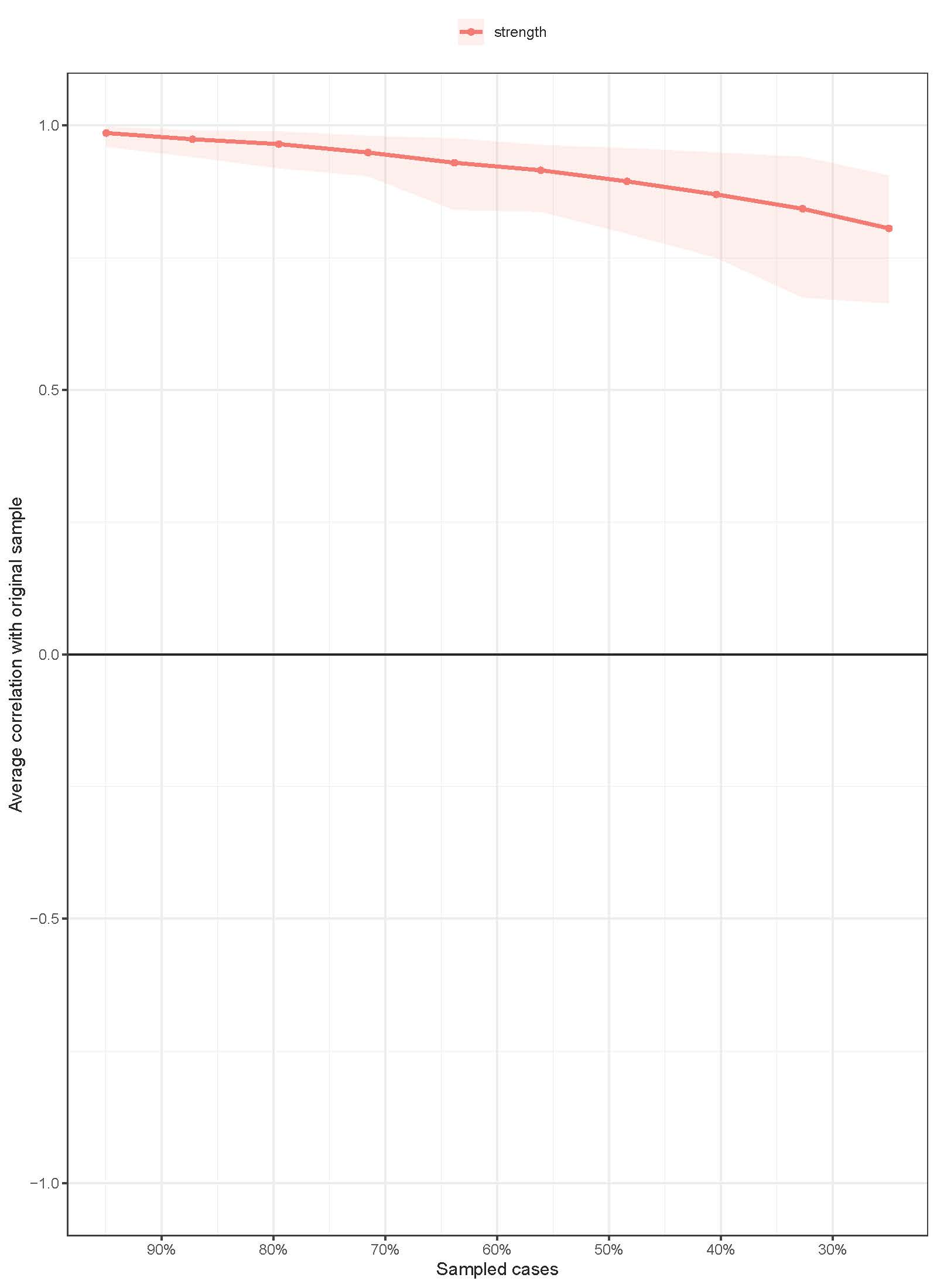


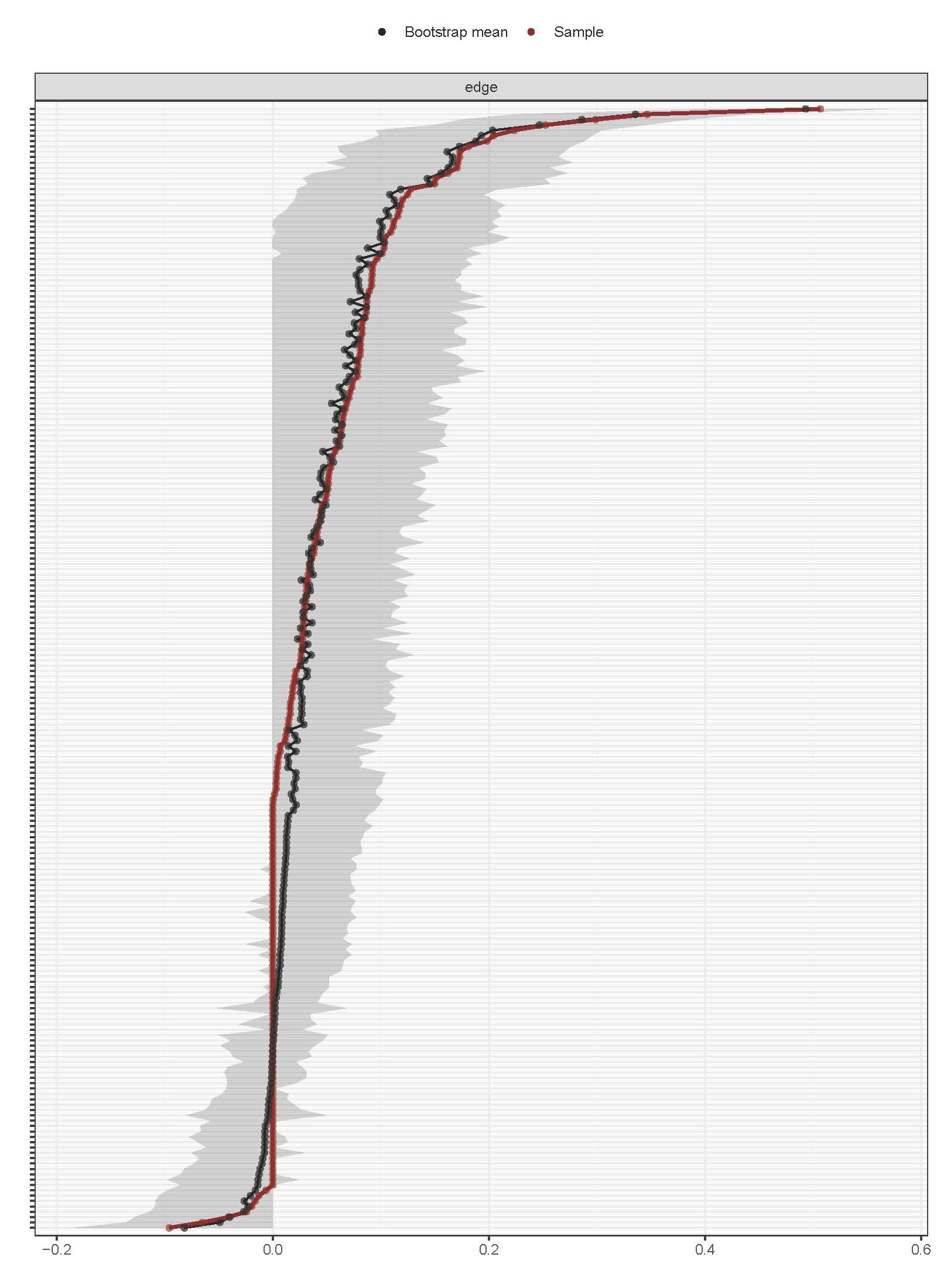
**Supplementary Materials 4.** Accuracy of edge weights for the residual symptom network post-CBT measured using the BDI-II. Bootstrapped confidence intervals (CIs) of the edge weights, derived from non-parametric bootstrap (nBoot=1,000) analyses using R package bootnet (Epskamp et al., 2018). The red line indicates the edge weight values and the grey area is the 95% C

**Supplementary Materials 5.** Strength of residual symptom co-occurrences/edges post-CBT measured using the BDI-II.


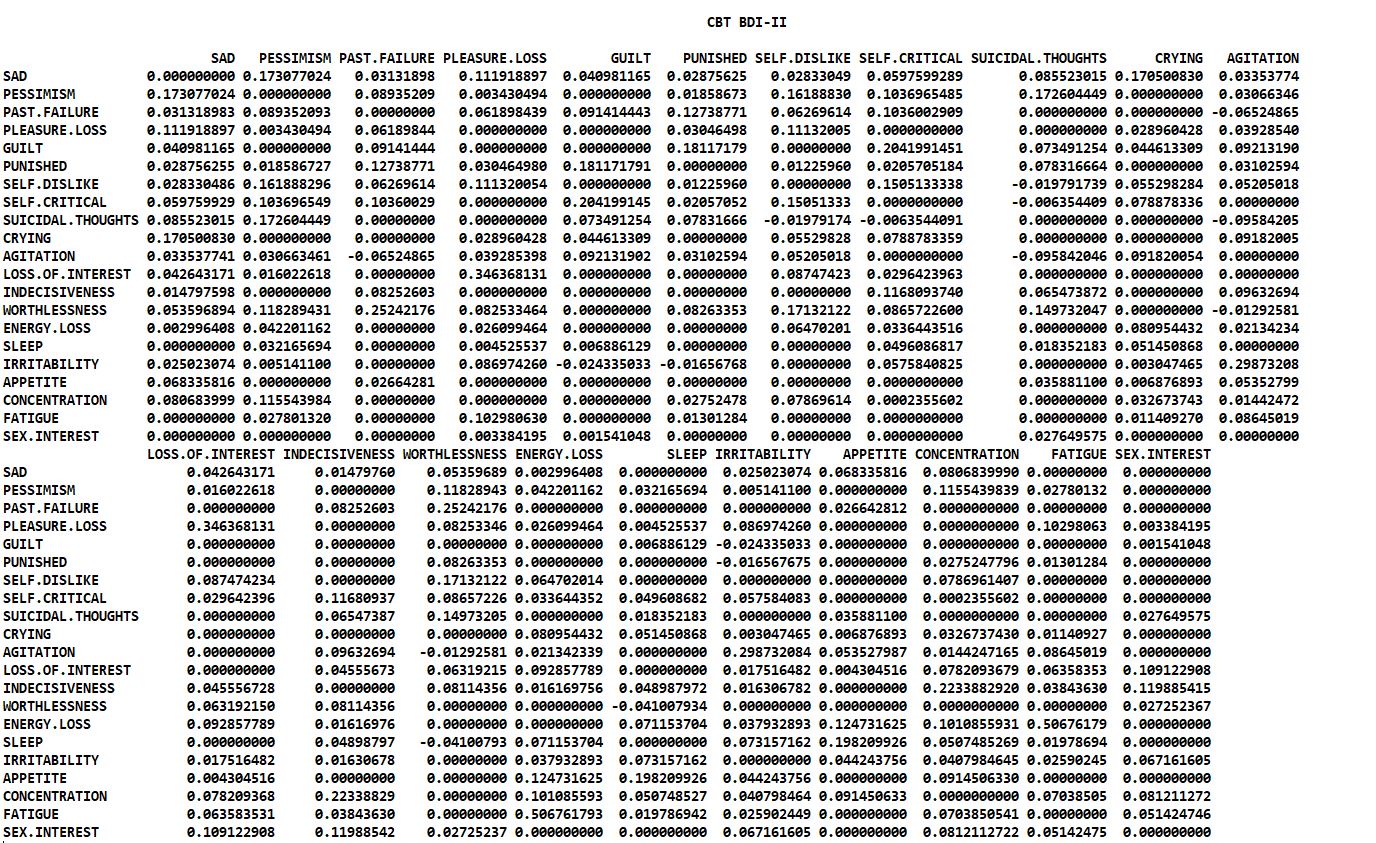


**Supplementary Materials 6.** Residual symptom network centrality plot post-CBT and ADM’s, measured using the HDRS-17.


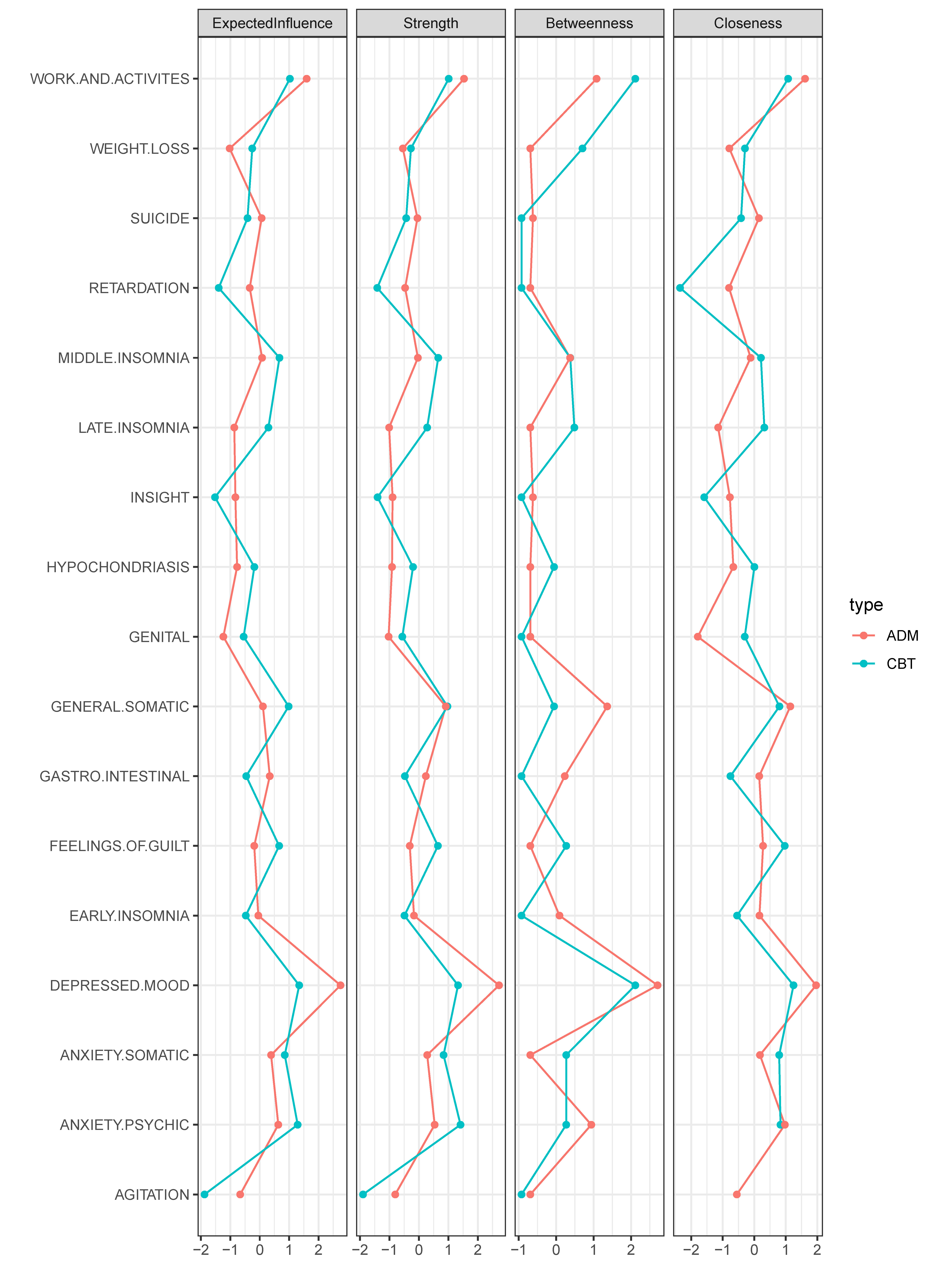


**Supplementary Figure 7.** Case drop bootstrap for the residual symptom network post-CBT measured using the HDRS-17.

**
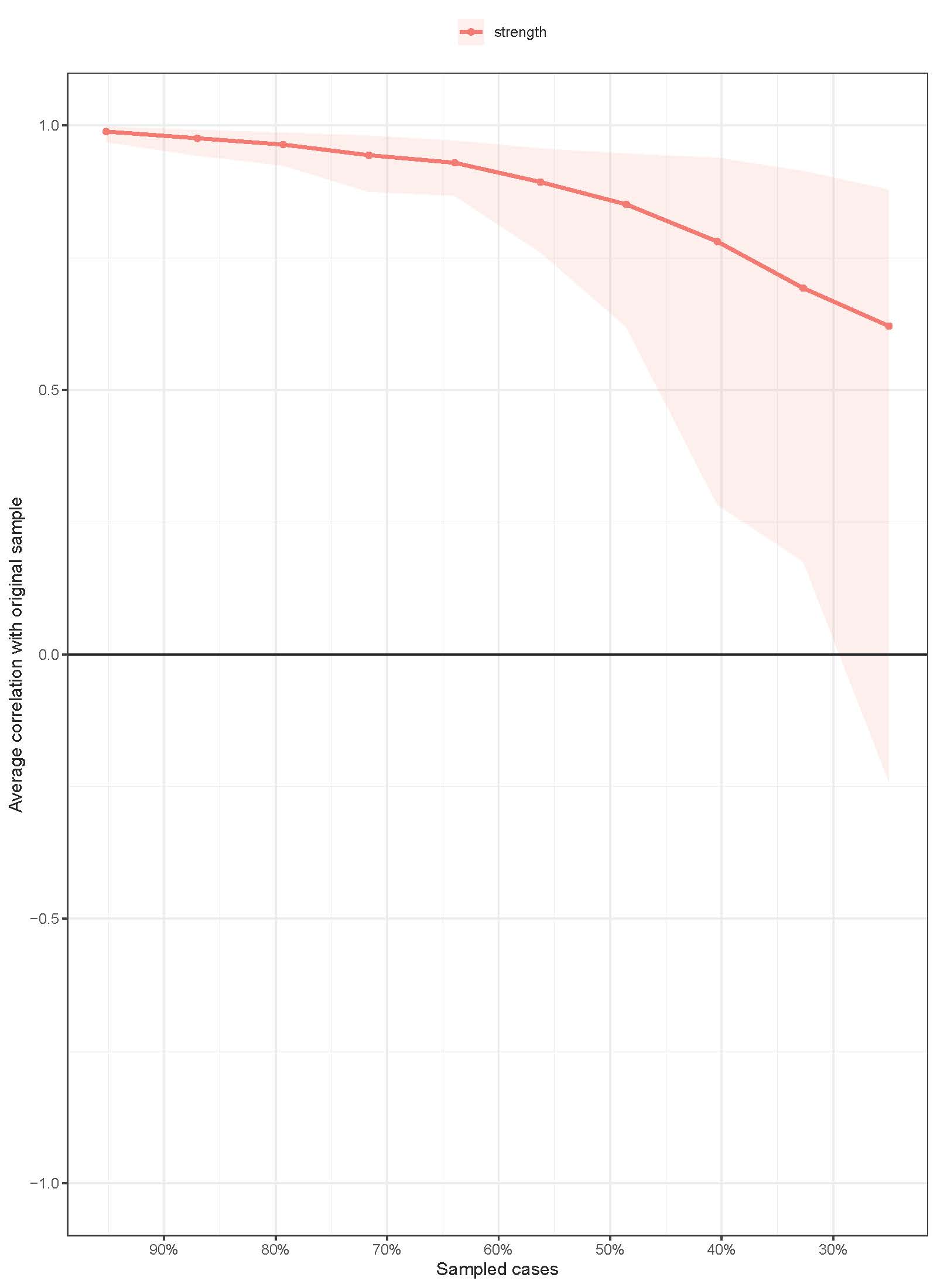
**

**Supplementary Figure 8.** Case drop bootstrap for the residual symptom network post-ADM measured using the HDRS-17.


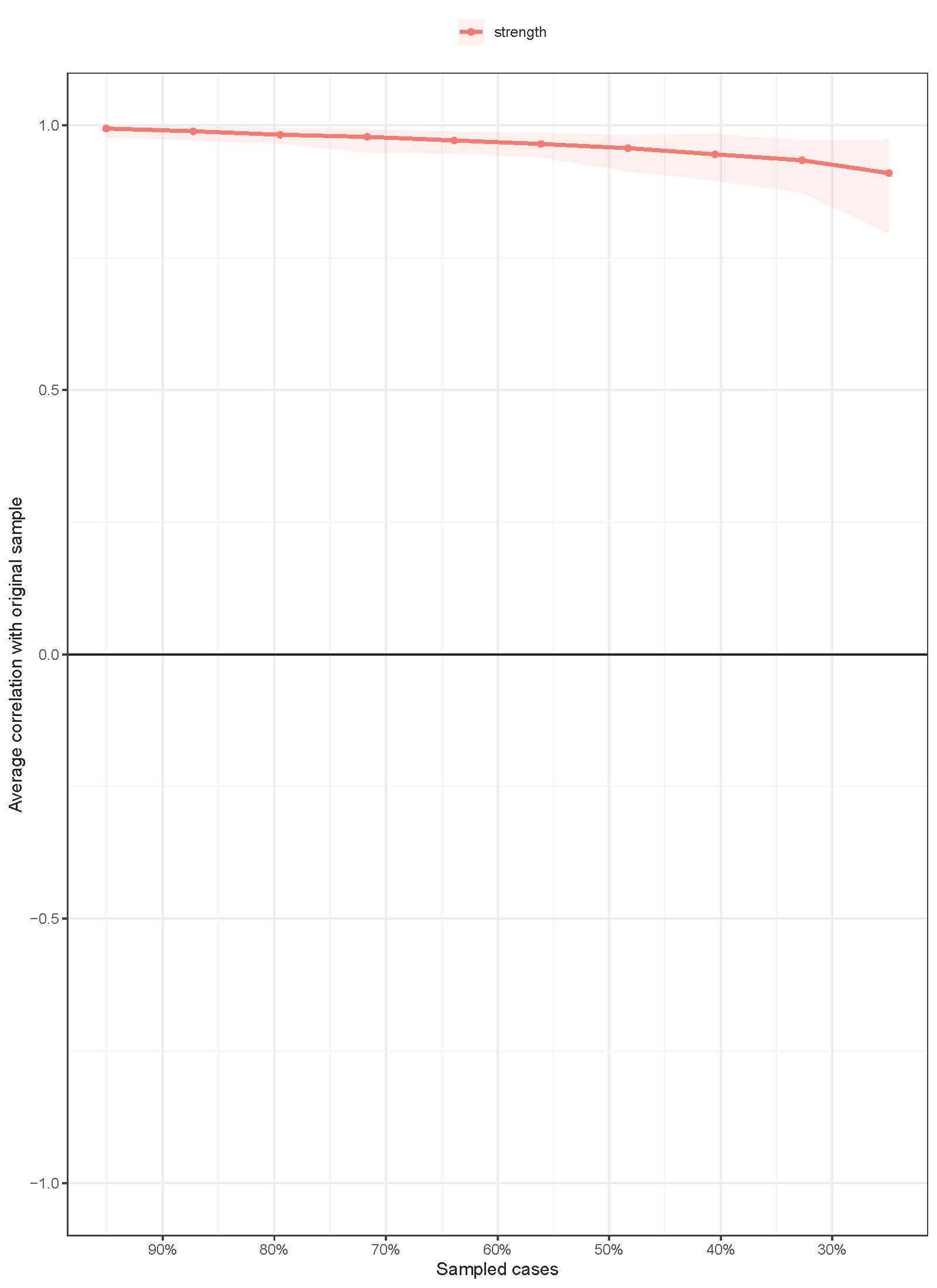


**Supplementary Figure 9.** Accuracy of edge weights for the residual symptom network post-CBT measured using the HDRS-17.


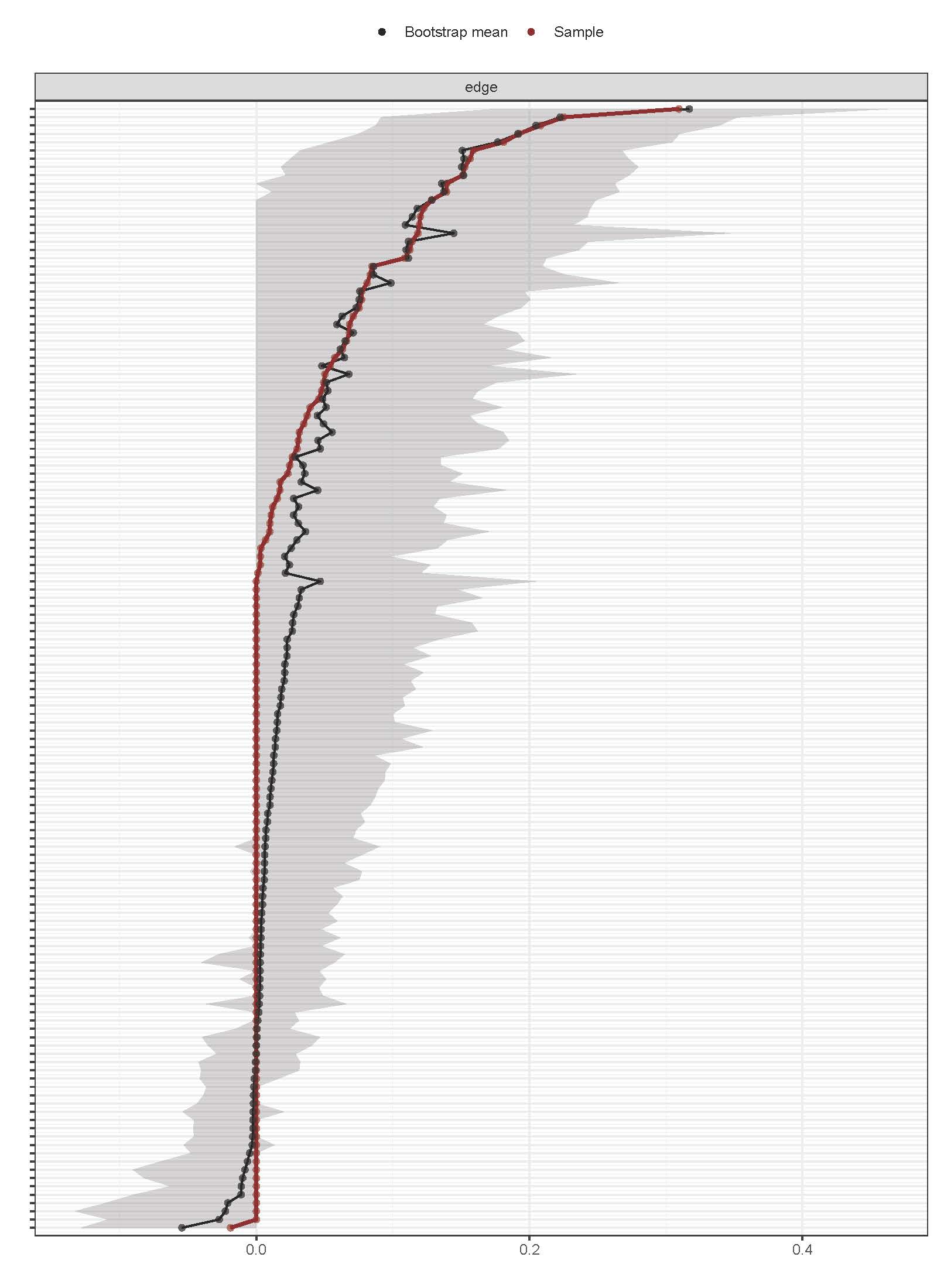


**Supplementary Figure 10.** Accuracy of edge weights for the residual symptom network post-ADM measured using the HDRS-17.


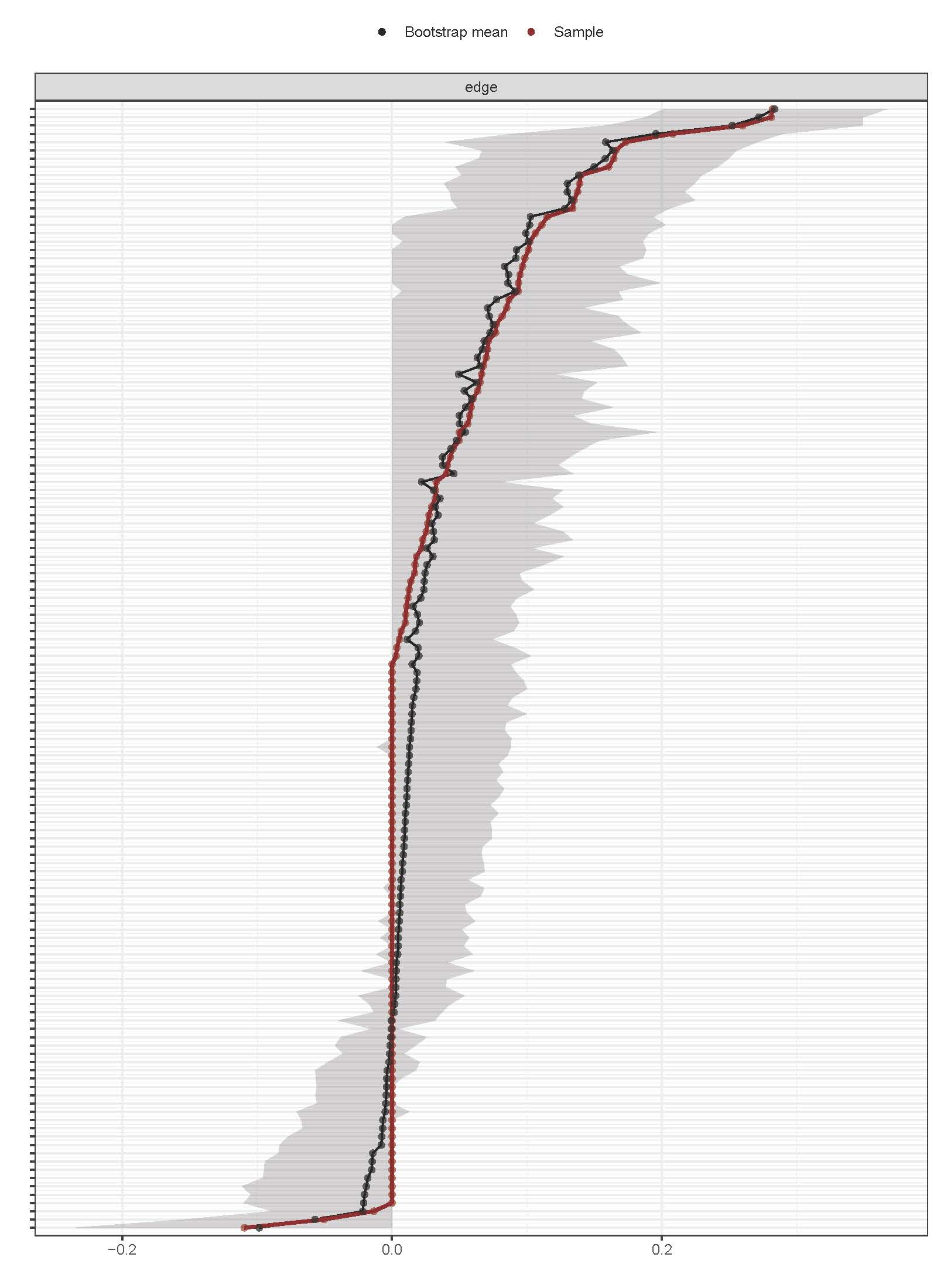


**Supplementary Figure 11.** Strength of residual symptom co-occurrences/edges post-CBT measured using the HDRS-17..


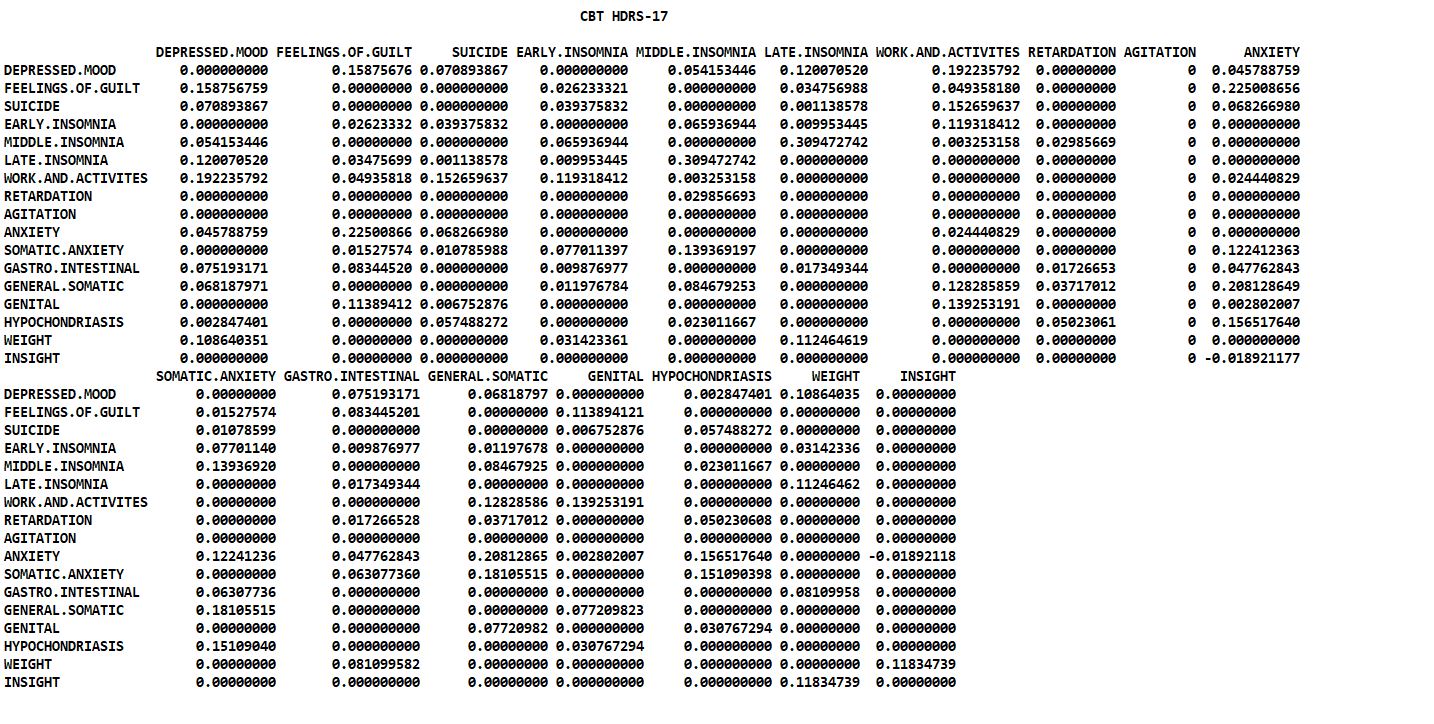


**Supplementary Figure 12.** Strength of residual symptom co-occurrences/edges post-ADM’s measured using the HDRS-17.


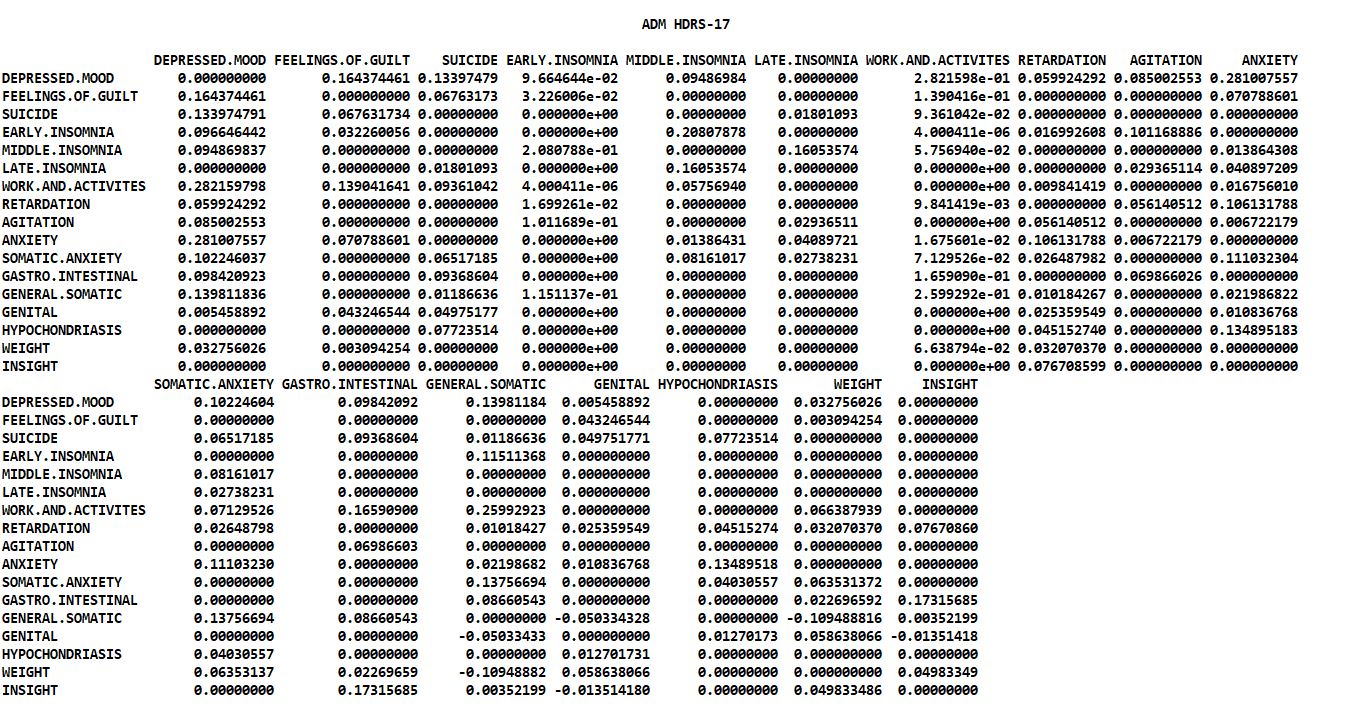


**Supplementary Materials 13.** Residual symptom network centrality plot post-CBT, and ADM’S measured using the MADRS.


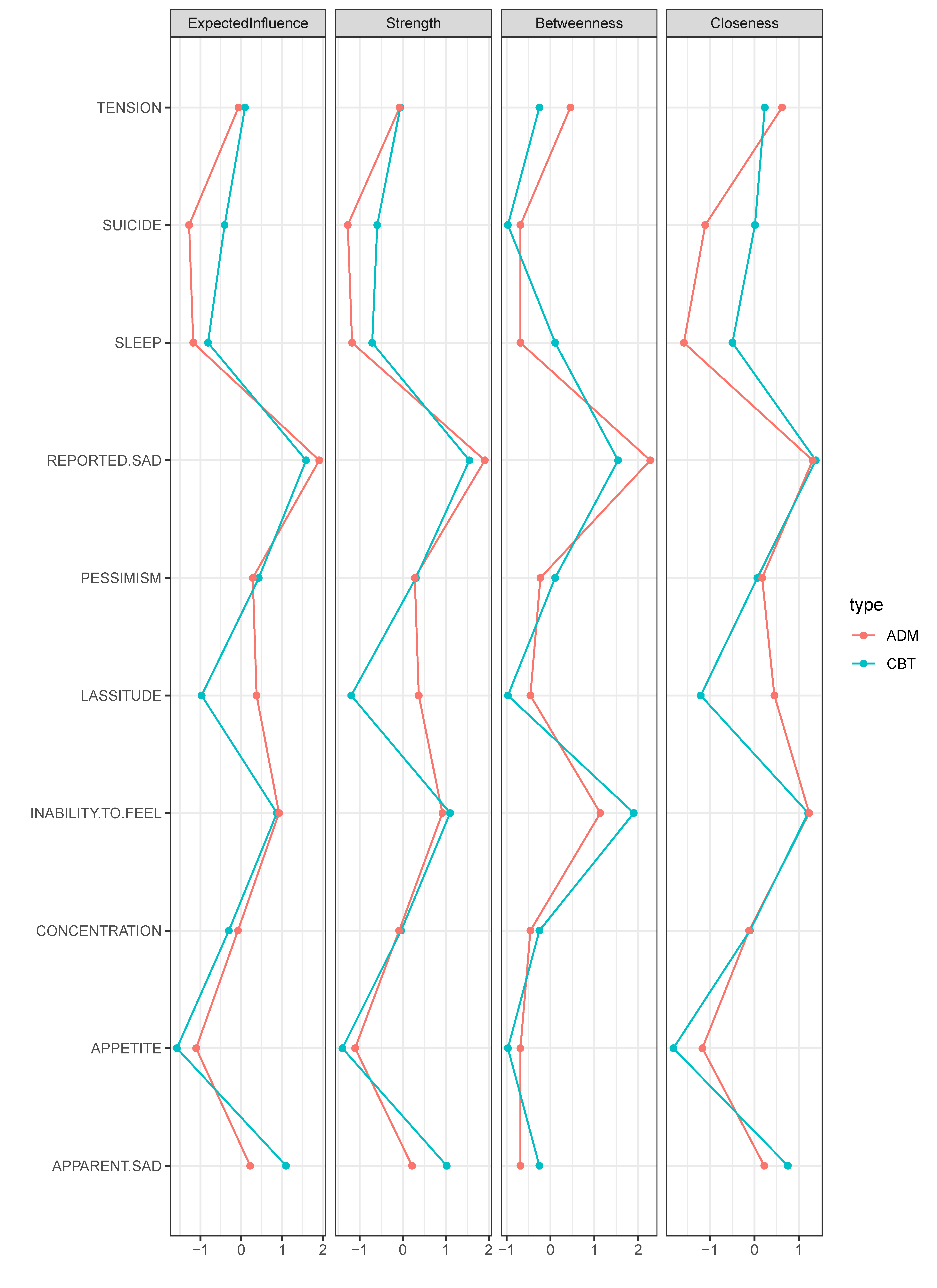


**Supplementary Materials 14.** Case drop bootstrap for the residual symptom network post-CBT measured using the MADRS.


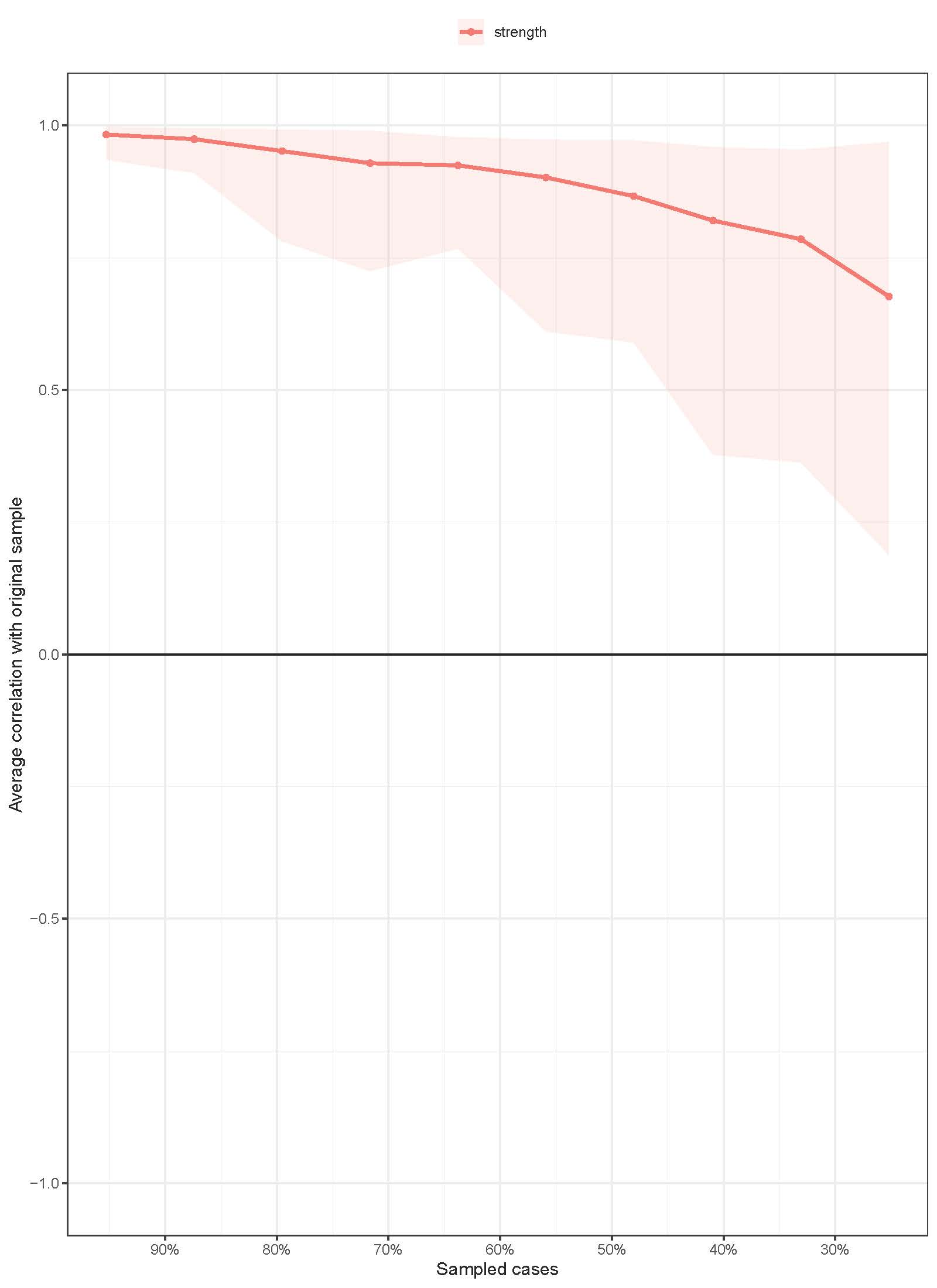


**Supplementary Materials 15.** Case drop bootstrap for the residual symptom network post-ADM measured using the MADRS.


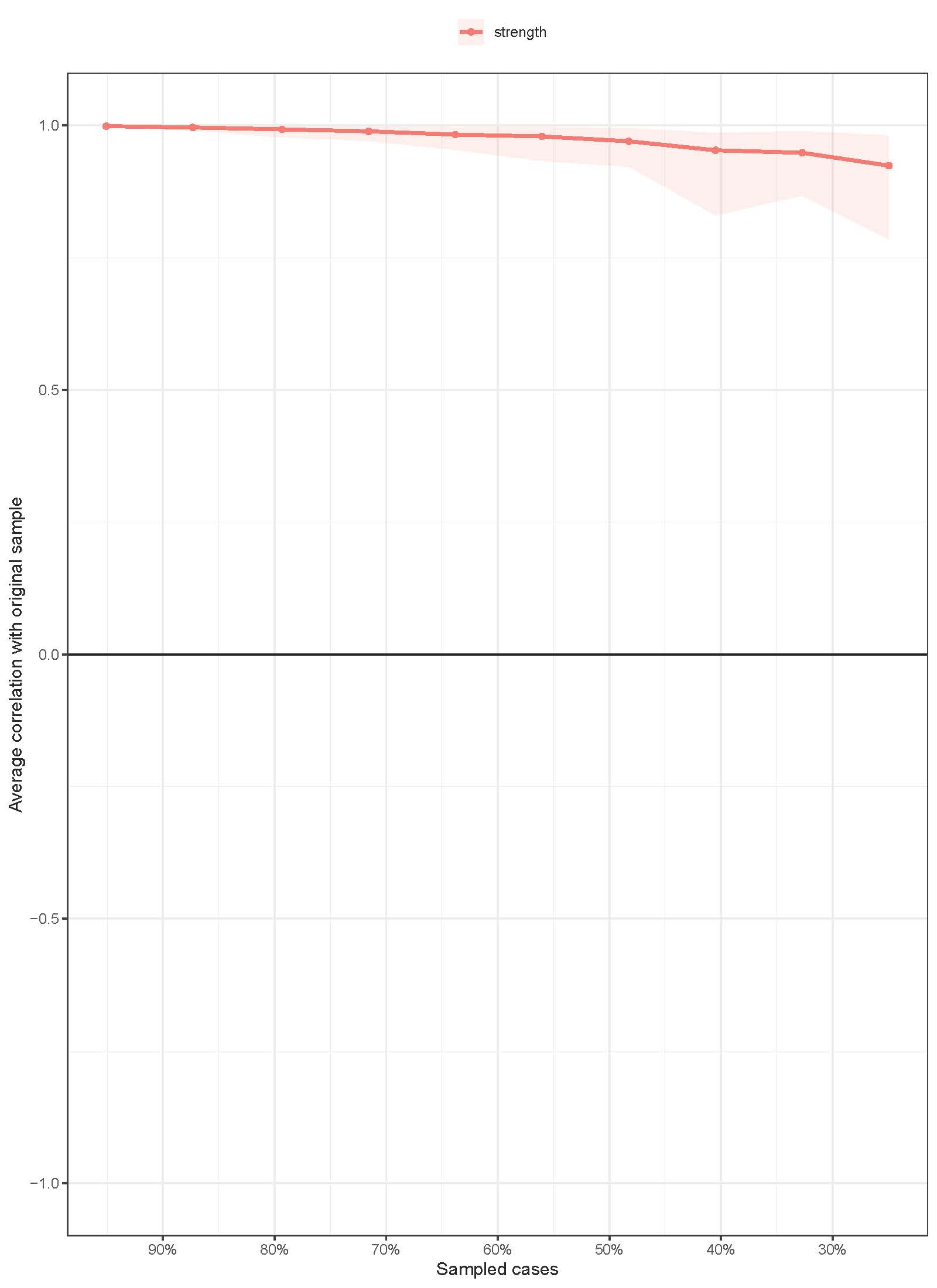


**Supplementary Materials 16.** Accuracy of edge weights for the residual symptom network post-CBT measured using the MADRS.


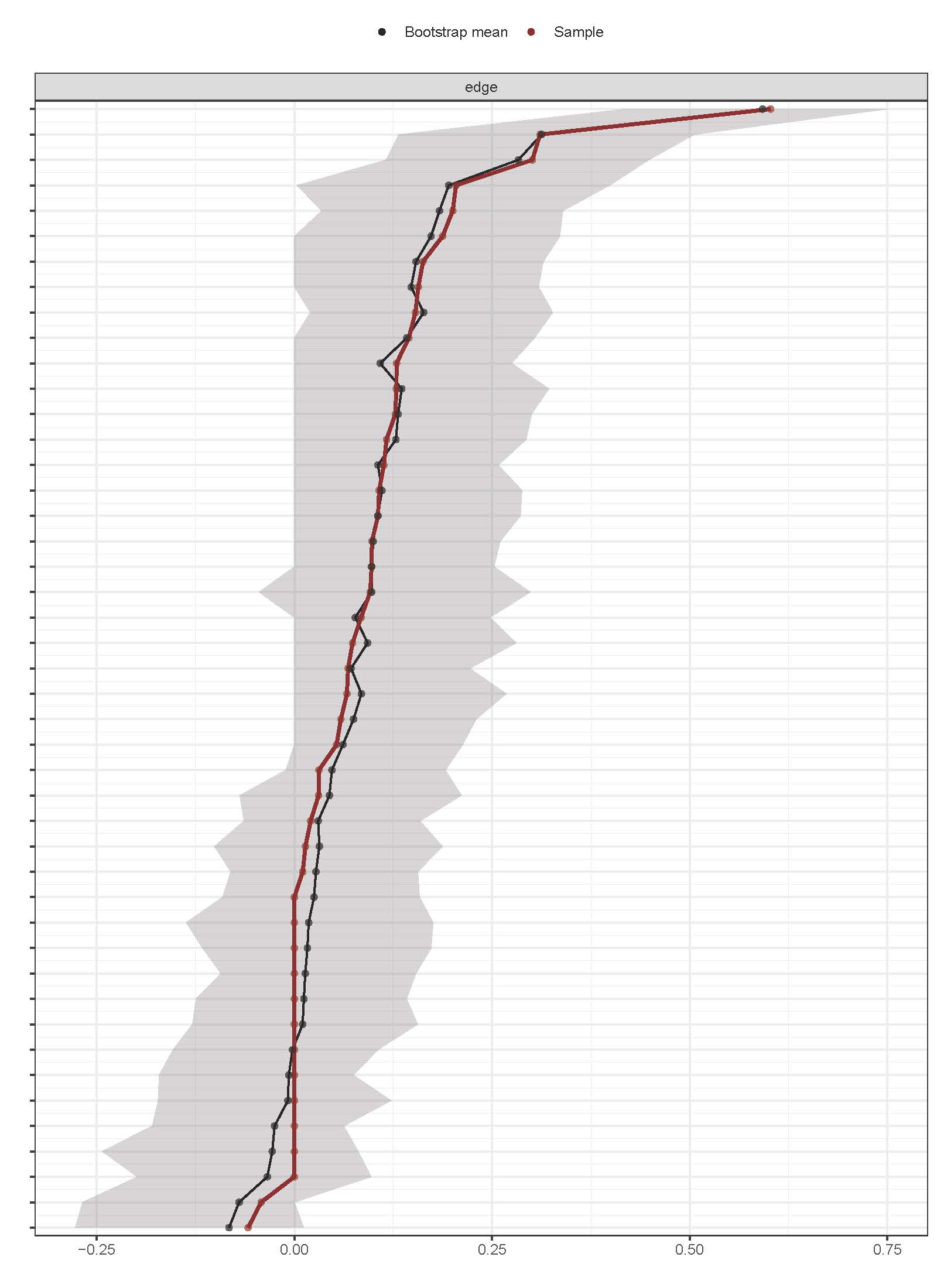


**Supplementary Materials 17.** Accuracy of edge weights for the residual symptom network post-ADM measured using the MADRS


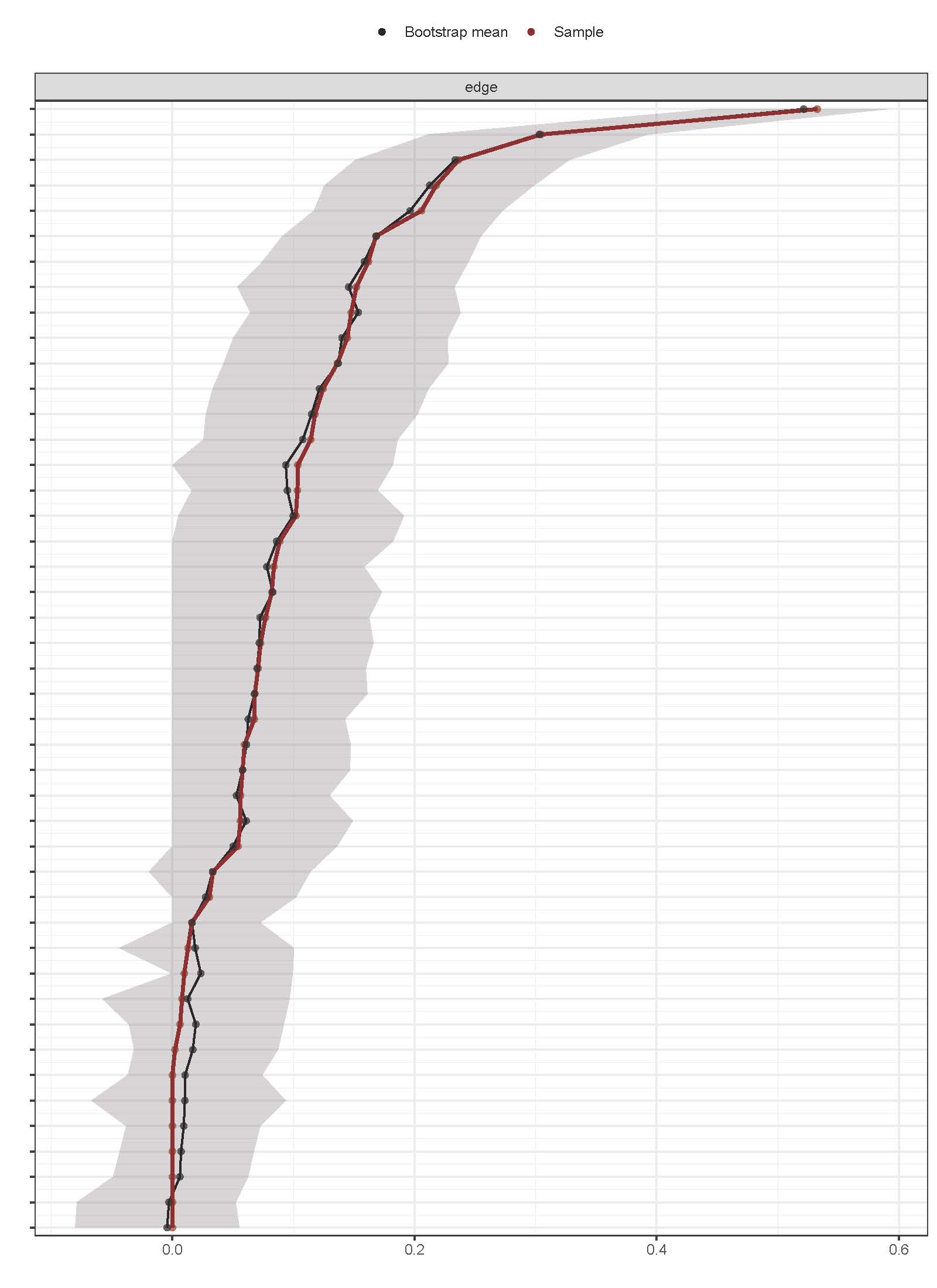


**Supplementary Figure 18.** Strength of residual symptom co-occurrences/edges post-CBT measured using the MADRS..**Supplementary Figure 19.** Strength of residual symptom co-occurrences/edges post-ADM’s measured using the MADRS.


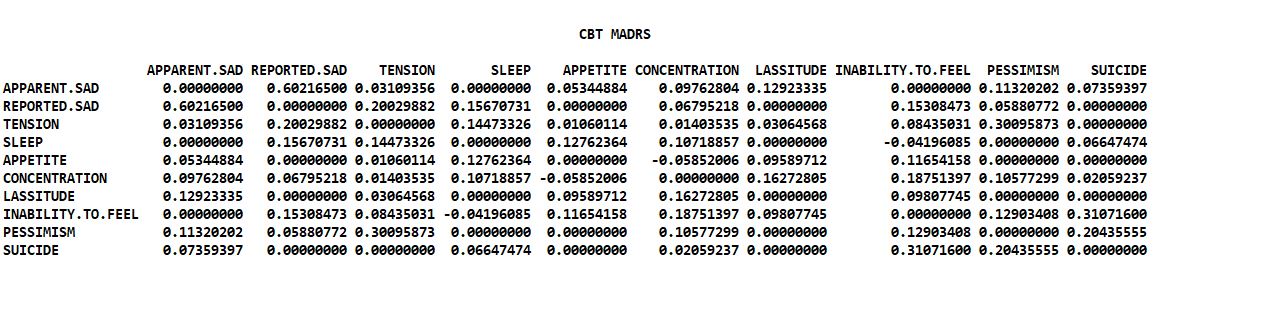

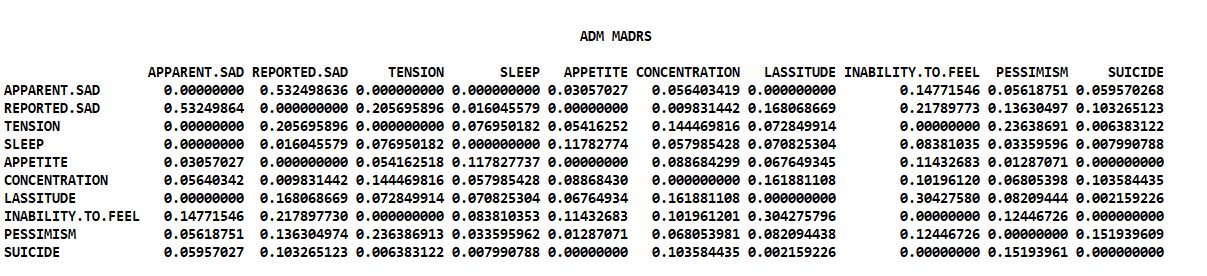


**Supplementary Materials 20.** Extracted data for all samples meeting systematic review criteria and eligible for IPD.

| Wave | | Name | Year | Design | Diagnosis Tool | Treatment | N Pre | N Post | Symptom Scales | | Gender % (Males) | Age Mean |
| --- | --- | --- | --- | --- | --- | --- | --- | --- | --- | --- | --- | --- |
| 1 | Abdallah | | 2014 | NRCT | DSM-IV-TR | CBT | 42 | 30 | HDRS | BDI | 40 | 42.6 |
| 1 | Akkaya | | 2006 | NRCT | DSM-IV-TR | VENLAFAXINE | 43 |  | HDRS |  | 18.6 | 42 |
| 1 | Alexopolous | | 2012 | NRCT | DSM-IV | ESCITALOPRAM | 32 | 32 | HDRS | MADRS | N/A | 69 |
| 1 | Alexopolous | | 2009 | NRCT | DSM-IV | ESCITALOPRAM | 27 | 27 | HDRS |  | N/A | 70.15 |
| 1 | Alexopolous | | 2008 | NRCT | DSM-IV | ESCITALOPRAM | 48 | 48 | HDRS |  | N/A | 70.25 |
| 1 | Alexopolous | | 2010 | NRCT | DSM-IV | ESCITALOPRAM | 32 | 32 | HDRS |  | N/A | 70.45 |
| 1 | Alexopolous | | 2007 | NRCT | DSM-IV | ESCITALOPRAM | 12 | 12 | HDRS |  | N/A | 71.2 |
| 1 | Alexopolous | | 2007 | NRCT | DSM-IV | ESCITALOPRAM | 40 | 40 | HDRS |  | 41.46 | 71.5 |
| 1 | Alexopolous | | 2015 | NRCT | DSM-IV | ESCITALOPRAM | 53 | 53 | HDRS |  | N/A | 72.18 |
| 1 | Alladin | | 2007 | NRCT | DSM-IV-TR | CBT | 42 | 42 | BDI-II |  | 42.86 | 36.5 |
| 1 | Allard | | 2004 | RCT | DSM-IV | VENLAFAXINE | 73 | 73 | MADRS |  | 20.55 | 73.6 |
| 1 | Allen | | 2018 | NRCT | DSM-IV-TR | ESCITALOPRAM | 210 | 210 | MADRS |  | 27 | 35.22 |
| 1 | Altenstien | | 2017 | NRCT | DSM-IV | CBT | 144 | 72 | BDI-II |  | 46.03 | 39.65 |
| 1 | Alvarez | | 2012 | RCT | DSM-IV-TR | VENLAFAXINE | 113 | 113 | HDRS | MADRS | 45.1 | 45 |
| 1 | Amini | | 2005 | RCT | DSM-IV | MIRTAZAPINE | 16 | 16 | HDRS |  | 31.3 | 35 |
| 1 | Amore | | 1997 | NRCT | DSM-IV | VENLAFAXINE | 28 | 28 | HDRS |  | 35.71 | 72.2 |
| 1 | Amsterdam | | 2013 | NRCT | DSM-IV-TR | CBT | 20 | 20 | HDRS |  | 75 | 41 |
| 1 | Amsterdam | | 2000 | NRCT | DSM-IV | VENLAFAXINE | 32 | 32 | HDRS | MADRS | 0 | 37 |
| 1 | Amsterdam | | 1998 | RCT | DSM-IV | VENLAFAXINE | 48 | 48 | HDRS | MADRS | 35.42 | 43 |
| 1 | Araya | | 2006 | NRCT | DSM-IV | VENLAFAXINE | 18 | 18 | HDRS |  | 42.85 | 43.25 |
| 1 | Arias | | 1998 | NRCT | DSM-IV | VENLAFAXINE | 34 | 34 | HDRS |  | 19.6 | 53.2 |
| 1 | Asano | | 2013 | NRCT | DSM-IV-TR | CBT | 22 | 20 | BDI |  | 30 | 37.1 |
| 1 | Ataoglu | | 2009 | NRCT | DSM-IV | ESCITALOPRAM | 15 | 15 | MADRS |  | 26.66 | 37.1 |
| 1 | Aydemir | | 2005 | NRCT | DSM-IV | VENLAFAXINE | 10 | 10 | HDRS |  | 20 | 31.8 |
| 1 | Aydin | | 2011 | NRCT | DSM-IV-TR | ESCITALOPRAM | 50 | 47 | HDRS |  | 0 | 30 |
| 1 | Azvedo | | 2017 | RCT | DSM-IV | CBT | 60 | 60 | HDRS |  | 26.67 | 24.01 |
| 1 | Bagby | | 1997 | NRCT | DSM-IV | VENLAFAXINE | 25 | 25 | HDRS |  | 24 | 39.2 |
| 1 | Bagby A | | 2008 | RCT | DSM-IV | CBT | 56 | 36 | HDRS |  | N/A | 41.9 |
| 1 | Bagby B | | 2008 | RCT | DSM-IV | CBT | 90 | 69 | HDRS |  | N/A | 41.9 |
| 1 | Baldomero | | 2005 | NRCT | DSM-IV | VENLAFAXINE | 1830 | 1632 | HDRS |  | 27.2 | 46.6 |
| 1 | Balestri | | 2016 | NRCT | DSM-IV-TR | VENLAFAXINE | 355 | 355 | MADRS |  | 34.65 | 46.71 |
| 1 | Balldwin | | 2006 | RCT | DSM-IV | ESCITALOPRAM | 165 | 130 | MADRS |  | 45 | 44.9 |
| 1 | Ballus | | 2000 | RCT | ICD-10 | VENLAFAXINE | 41 | 41 | MADRS | HDRS | 12.19 | 44 |
| 1 | Banach | | 2017 | NRCT | DSM-IV | VENLAFAXINE | 30 | 30 | HDRS |  | 0 | 37.7 |
| 1 | Bandu | | 2018 | NRCT | DSM-IV | ESCITALOPRAM | 7 | 7 | HDRS |  | 57.14 | N/A |
| 1 | Bares | | 2015 | RCT | DSM-IV | VENLAFAXINE | 25 | 25 | MADRS |  | 24 | 44.4 |
| 1 | Bares | | 2008 | NRCT | DSM-IV | VENLAFAXINE | 26 | 25 | MADRS |  | 32 | 44.5 |
| 1 | Bares | | 2009 | RCT | DSM-IV | VENLAFAXINE | 31 | 31 | MADRS |  | 20 | 44.7 |
| 1 | Basoglu | | 2009 | NRCT | DSM-IV | ESCITALOPRAM | 15 | 15 | MADRS |  | 45.7 | N/A |
| 1 | Basterzi | | 2010 | RCT | DSM-IV | VENLAFAXINE | 36 | 12 | HDRS |  | 28.99 | 31 |
| 1 | Basterzi | | 2009 | RCT | DSM-IV | VENLAFAXINE | 21 | 21 | HDRS |  | 33.33 | 31 |
| 1 | Basto | | 2018 | RCT | DSM-IV | CBT | 10 | 10 | QQ-10 |  | 18 | 34.55 |
| 1 | Basu | | 2017 | NRCT | DSM-IV-TR | ESCITALOPRAM | 80 | 80 | MADRS |  | 61.86 | 35.13 |
| 1 | Behnke | | 2003 | RCT | DSM-IV | MIRTAZAPINE | 176 | 176 | HDRS | MADRS | 44.3 | 42 |
| 1 | Benkert | | 2000 | NRCT | DSM-IV | MIRTAZAPINE | 135 | 135 | HDRS | MADRS | 37 | 47.2 |
| 1 | Benkert | | 2006 | RCT | DSM-IV | MIRTAZAPINE | 127 | 127 | HDRS | MADRS | N/A | N/A |
| 1 | Benkert | | 1996 | RCT | DSM-III-R | VENLAFAXINE | 85 | 85 | HDRS | MADRS | 30.59 | 48.9 |
| 1 | Benkert B | | 2006 | RCT | DSM-IV | VENLAFAXINE | 115 | 115 | HDRS | MADRS | N/A | N/A |
| 1 | Berking | | 2013 | RCT | DSM-IV | CBT | 237 | 229 | BDI |  | 20 | 45.67 |
| 1 | Bernecker | | 2016 | NRCT | DSM-IV | CBT | 36 | 36 | HDRS | BDI-II | 22.2 | 42.89 |
| 1 | Bertschy | | 2005 | NRCT | ICD-10 | VENLAFAXINE | 27 | 27 | MADRS |  | 37.04 | 42 |
| 1 | Bertschy | | 2003 | NRCT | ICD-10 | VENLAFAXINE | 43 | 43 | MADRS |  | N/A | N/A |
| 1 | Blier | | 2009 | RCT | DSM-IV | MIRTAZAPINE | 21 | 21 | MADRS |  | 71.43 | 46 |
| 1 | Bobes | | 2002 | NRCT | DSM-IV | VENLAFAXINE | 12 | 12 | HDRS |  | 41.7 | 40.8 |
| 1 | Bobo | | 2016 | NRCT | DSM-IV | ESCITALOPRAM | N/A | N/A | HDRS |  | 38.2 | 39.1 |
| 1 | Bodenmann | | 2008 | RCT | DSM-IV | CBT | 20 | 19 | BDI | HDRS | 35 | 44.35 |
| 1 | Borkowska | | 2007 | NRCT | DSM-IV | MIRTAZAPINE | 60 | 60 | HDRS |  | 32.39 | 44 |
| 1 | Bose | | 2012 | RCT | DSM-IV | ESCITALOPRAM | 229 | 182 | MADRS |  | 40 | 41.6 |
| 1 | Bose | | 2008 | RCT | DSM-IV | ESCITALOPRAM | 130 | 130 | MADRS |  | 41 | 68.1 |
| 1 | Boulenger | | 2006 | RCT | DSM-IV-TR | ESCITALOPRAM | 232 | 229 | HDRS | MADRS | 33 | 43.8 |
| 1 | Boyarsky | | 1999 | NRCT | DSM-IV | MIRTAZAPINE | 18 | 18 | HDRS |  | 28 | N/A |
| 1 | Bruder | | 1997 | NRCT | DSM-III-R | CBT | 31 | 31 | HDRS |  | 25.81 | 38.35 |
| 1 | Bruijn | | 1196 | RCT | DSM-III-R | MIRTAZAPINE | 54 | 54 | HDRS | MADRS | 22.22 | 45 |
| 1 | Brunelin | | 2014 | RCT | DSM-IV | VENLAFAXINE | 55 | 51 | HDRS | MADRS + BDI | 29.09 | 56.2 |
| 1 | Brunnauer | | 2008 | NRCT | DSM-IV-TR | MIRTAZAPINE | 20 | 20 | HDRS | BDI | 55 | 49.2 |
| 1 | Brunnauer | | 2015 | NRCT | ICD-10 | VENLAFAXINE | 20 | 20 | HDRS | BDI | 45 | 50.8 |
| 1 | Brunoni | | 2017 | RCT | DSM-5 | ESCITALOPRAM | 91 | 90 | HDRS | MADRS | 33 | 41.8 |
| 1 | Bukh | | 2009 | NRCT | ICD-10 | VENLAFAXINE | 48 | 25 | HDRS | BDI | 40 | 43.3 |
| 1 | Bulmash | | 2009 | NRCT | DSM-IV-TR | CBT | 37 | 37 | HDRS |  | 32.5 | 42.93 |
| 1 | Burke | | 2002 | RCT | DSM-IV | ESCITALOPRAM | 243 | 243 | HDRS | MADRS | 31 | 40.15 |
| 1 | Burkhouse | | 2016 | NRCT | DSM-IV | CBT | 52 | 52 | BDI-II |  | 23.1 | 23.94 |
| 1 | Burns | | 1992 | NRCT | DSM-III | CBT | 185 | 185 | BDI |  | 41.1 | 36.1 |
| 1 | Burns | | 2000 | NRCT | DSM-III | CBT | 399 | 399 | BDI |  | N/A | N/A |
| 1 | Campayo | | 2008 | NRCT | DSM-IV | MIRTAZAPINE | 496 | 496 | HDRS |  | 34 | 40.09 |
| 1 | Carney | | 2011 | NRCT | DSM-IV | CBT | 24 | 24 | BDI-II |  | 33.3 | 40 |
| 1 | Carney | | 2007 | NRCT | DSM-IV | CBT | 54 | 54 | HDRS |  | 41 | 36.7 |
| 1 | Carter | | 2010 | NRCT | DSM-III-R | CBT | N/A | N/A | BDI-II | HDRS | 27.68 | 35.2 |
| 1 | Carter | | 2013 | RCT | DSM-IV | CBT | 50 | 38 | BDI-II | MADRS, HDRS | 34 | 38.2 |
| 1 | Carter | | 2005 | NRCT | DSM-IV | CBT | 49 | 4 | EPDS |  | 0 | N/A |
| 1 | Cattaneo | | 2010 | NRCT | DSM-IV | ESCITALOPRAM | 21 | 21 | MADRS |  | 19.05 | 43.57 |
| 1 | Cervera | | 2003 | NRCT | DSM-IV | VENLAFAXINE | 833 | 833 | HDRS |  | 32 | 46 |
| 1 | Chang | | 2014 | NRCT | DSM-IV | MIRTAZAPINE | 283 | 283 | HDRS |  | N/A | N/A |
| 1 | Chang | | 2015 | NRCT | DSM-IV | VENLAFAXINE | 54 | 54 | HDRS | BDI | 29.6 | 37.5 |
| 1 | Chang | | 2013 | RCT | DSM-IV | VENLAFAXINE | 22 | 22 | HDRS |  | 28 | 38.6 |
| 1 | Chang B | | 2015 | NRCT | DSM-IV | MIRTAZAPINE | 270 | 270 | HDRS | BDI | 23.7 | 50.8 |
| 1 | Charlier | | 2000 | NRCT | DSM-IV | VENLAFAXINE | 9 | 9 | CGI |  | N/A | 47.6 |
| 1 | Charlier | | 2002 | NRCT | DSM-IV | VENLAFAXINE | 89 | 89 | MADRS |  | N/A | N/A |
| 1 | Chaudhry | | 1998 | RCT | DSM-III-R | CBT | 59 | 59 | HDRS |  | N/A | 40 |
| 1 | Chen | | 2011 | RCT | DSM-IV | ESCITALOPRAM | 51 | 51 | GDS |  | 38.2 | 68.9 |
| 1 | Chen | | 2018 | RCT | DSM-IV-TR | VENLAFAXINE | 41 | 41 | HDRS |  | N/A | N/A |
| 1 | Cheng | | 2017 | RCT | DSM-IV | ESCITALOPRAM | 48 | 48 | HDRS |  | 45.83 | 29.41 |
| 1 | Chi | | 2010 | NRCT | DSM-IV | VENLAFAXINE | 117 | 117 | HDRS |  | 21 | 36.2 |
| 1 | Chiesa | | 2014 | NRCT | DSM-IV | VENLAFAXINE | 184 | 184 | HDRS |  | 30 | 43.62 |
| 1 | Chokka | | 2008 | NRCT | DSM-IV | ESCITALOPRAM | 618 | 459 | MADRS |  | 35 | 43.4 |
| 1 | Chuang | | 2014 | NRCT | DSM-IV-TR | VENLAFAXINE | 82 | 82 | HDRS |  | 32.9 | 39.2 |
| 1 | Cimen | | 2015 | NRCT | DSM-IV | ESCITALOPRAM | 18 | 18 | MADRS |  | 38.88 | 42.17 |
| 1 | Ciusani | | 2004 | NRCT | ICD-10 | VENLAFAXINE | 10 | 10 | MADRS |  | 30 | 39 |
| 1 | Clerc | | 1994 | RCT | DSM-III-R | VENLAFAXINE | 33 | 33 | MADRS | HDRS | 33.33 | 49 |
| 1 | Colonna | | 2005 | RCT | DSM-IV | ESCITALOPRAM | 175 | 144 | MADRS |  | N/A | 46 |
| 1 | Corruble | | 2013 | RCT | DSM-IV-TR | ESCITALOPRAM | 160 | 115 | HDRS |  | 31.3 | 42.8 |
| 1 | Corya | | 2006 | RCT | DSM-IV | VENLAFAXINE | 59 | 59 | MADRS |  | 27.5 | 45.7 |
| 1 | Costa e Silva | | 1998 | RCT | DSM-III-R | VENLAFAXINE | 196 | 196 | HDRS | MADRS | 19.39 | 40.5 |
| 1 | Craigie | | 2009 | NRCT | DSM-IV-TR | CBT | 116 | 77 | BDI-II |  | 48.28 | 34 |
| 1 | Crane | | 2017 | NRCT | DSM-IV | ESCITALOPRAM | 22 | 22 | HDRS |  | 32.65 | 36.94 |
| 1 | Cuijpers | | 2005 | RCT | DSM-IV | CBT | 199 | 199 | SCL-90 |  | 39 | 36 |
| 1 | Cunningham | | 1997 | RCT | DSM-III-R | VENLAFAXINE | 179 | 179 | HDRS | MADRS | 35.19 | 40.25 |
| 1 | Da Silva | | 2018 | NRCT | DSM-IV | CBT | 42 | 42 | BDI-II |  | 16.6 | N/A |
| 1 | Daly | | 2011 | NRCT | DSM-IV-TR | ESCITALOPRAM | N/A | N/A | HDRS |  | 31 | N/A |
| 1 | Daniel | | 2013 | NRCT | DSM-IV/ICD-10 | ESCITALOPRAM | 10 | 10 | HDRS |  | 50 | 42.6 |
| 1 | Danileviciute | | 2009 | NRCT | ICD-10 | MIRTAZAPINE | 779 | 687 | HDRS |  | 23.6 | 51.3 |
| 1 | Dannehl | | 2019 | RCT | DSM-IV | CBT | 34 | 34 | BDI-II |  | 44.1 | 37.2 |
| 1 | Davidson | | 2003 | NRCT | DSM-IV | VENLAFAXINE | 5 | 5 | HDRS |  | 80 | 27.8 |
| 1 | Davies | | 2003 | NRCT | ICD-10 | VENLAFAXINE | 7 | 7 | BDI | HDRS | 28.57 | 43 |
| 1 | De Grande da Silva | | 2016 | NRCT | DSM | CBT | 46 | 46 | BDI |  | 23.9 | 33.85 |
| 1 | Debonnel | | 2007 | NRCT | DSM-IV | VENLAFAXINE | 44 | 44 | HDRS | MADRS | 43.5 | 41.5 |
| 1 | Delini-Stula | | 2006 | NRCT | DSM-IV/ICD-10 | MIRTAZAPINE | 1121 | 914 | CGI |  | 41 | 48.6 |
| 1 | Demirci | | 2016 | NRCT | DSM-IV | ESCITALOPRAM | 41 | 41 | HDRS |  | 27 | 30.24 |
| 1 | Dermody | | 2016 | NRCT | DSM-IV | CBT | 125 | 125 | HDRS |  | 35 | 41.17 |
| 1 | Deuschle | | 2015 | NRCT | DSM-IV | VENLAFAXINE | 30 | 30 | HDRS |  | 36.05 | 49.85 |
| 1 | Deuschle | | 2013 | NRCT | DSM-IV | VENLAFAXINE | 27 | 27 | HDRS |  | 29.63 | 54.1 |
| 1 | Deuschle B | | 2015 | NRCT | DSM-IV | MIRTAZAPINE | 27 | 27 | HDRS |  | 36.05 | 49.85 |
| 1 | Deuschle B | | 2013 | NRCT | DSM-IV | MIRTAZAPINE | 29 | 29 | HDRS |  | 24.14 | 50.5 |
| 1 | Diaz | | 1998 | RCT | DSM-III-R | VENLAFAXINE | 70 | 70 | HDRS | MADRS | 35.71 | 37 |
| 1 | Dierick | | 1996 | RCT | DSM-III-R | VENLAFAXINE | 153 | 115 | HDRS |  | 35 | 43.7 |
| 1 | Dierick | | 1996 | NRCT | DSM-III-R | VENLAFAXINE | 116 | 85 | MADRS |  | 22 | 73.1 |
| 1 | Dijmidjian | | 2006 | RCT | DSM-IV | CBT | 45 | 39 | BDI-II | HDRS | 34 | 39.9 |
| 1 | Ditcher | | 2005 | NRCT | DSM-IV | VENLAFAXINE | 10 | 10 | BDI-II | HDRS | 40 | 42.6 |
| 1 | Doerig | | 2016 | NRCT | DSM-IV | CBT | 23 | 21 | BDI-II |  | 52.38 | 38.3 |
| 1 | Dolberg | | 2014 | NRCT | DSM-IV-TR | ESCITALOPRAM | 405 | 405 | MADRS |  | N/A | 73 |
| 1 | Domschke | | 2014 | NRCT | DSM-IV | ESCITALOPRAM | 94 | 94 | HDRS | BDI | 35.11 | 47.4 |
| 1 | Dorz | | 2001 | NRCT | DSM-IV | VENLAFAXINE | 61 | 61 | HDRS | MADRS | 37.7 | 51.3 |
| 1 | Dozios | | 2009 | RCT | DSM-IV | CBT | 25 | 1 | BDI-II | HDRS | 26 | 46.5 |
| 1 | Driessen | | 2013 | RCT | DSM-IV | CBT | 164 | 164 | HDRS |  | 31.1 | 38.27 |
| 1 | Droppa | | 2017 | NRCT | DSM-IV | VENLAFAXINE | 26 | 26 | MADRS |  | 19.23 | 66 |
| 1 | Dube | | 2010 | NRCT | DSM-IV-TR | ESCITALOPRAM | 62 | 62 | CGI |  | 64.5 | 34.3 |
| 1 | Dunlop | | 2012 | RCT | DSM-IV | CBT | 41 | 41 | HDRS | BDI | N/A | N/A |
| 1 | Dunlop B | | 2012 | RCT | DSM-IV | ESCITALOPRAM | 39 | 39 | HDRS | BDI | N/A | N/A |
| 1 | Easden | | 2018 | NRCT | DSM-IV-TR | CBT | 28 | 28 | BDI-II |  | 36 | 44.75 |
| 1 | Eddington | | 2015 | RCT | DSM-IV-TR | CBT | 27 | 27 | BDI-II |  | 14.8 | 37.89 |
| 1 | Egami | | 2013 | NRCT | DSM-IV | MIRTAZAPINE | 21 | 21 | HDRS | BDI | 47.62 | 52.1 |
| 1 | Eidelman | | 2019 | NRCT | DSM-IV | CBT | 34 | 34 | DASS |  | 37.84 | 36.81 |
| 1 | Ekeblad | | 2016 | RCT | DSM-IV | CBT | 48 | 28 | BDI-II | MADRS | 31.2 | 32 |
| 1 | Eker | | 2008 | NRCT | DSM-IV | VENLAFAXINE | 30 | 26 | HDRS |  | 30.77 | 44.1 |
| 1 | El-Hage | | 2015 | NRCT | DSM-IV | ESCITALOPRAM | 187 | 153 | MADRS |  | 41.17 | 45 |
| 1 | El-Haggar | | 2018 | NRCT | DSM-IV | ESCITALOPRAM | 40 | 40 | HDRS |  | 48.7 | 33.09 |
| 1 | Eller | | 2010 | NRCT | DSM-IV | ESCITALOPRAM | 129 | 129 | MADRS |  | 30.23 | 31.7 |
| 1 | Eller | | 2008 | NRCT | DSM-IV | ESCITALOPRAM | 100 | 100 | MADRS |  | 35 | 32.1 |
| 1 | Entsuah | | 1998 | RCT | DSM-III-R | VENLAFAXINE | 158 | 158 | HDRS |  | 53.5 | 43.7 |
| 1 | Estupina Puig | | 2012 | NRCT | DSM | CBT | 69 | 44 | BDI-II |  | 26.1 | 35.41 |
| 1 | Fava | | 1997 | RCT | DSM-IV | VENLAFAXINE | 20 | 20 | HDRS |  | 55 | 36.5 |
| 1 | Fava A | | 2011 | RCT | DSM-IV | ESCITALOPRAM | 190 | 95 | HDRS |  | 36.3 | 43 |
| 1 | Fava B | | 2011 | NRCT | DSM-IV | MIRTAZAPINE | 103 | 103 | HDRS |  | 38.83 | 44 |
| 1 | Fiedorowicz | | 2010 | NRCT | DSM-IV-TR | ESCITALOPRAM | 23 | 23 | MADRS |  | 26 | 31 |
| 1 | Florio | | 2017 | NRCT | DSM-IV | ESCITALOPRAM | 70 | 70 | HDRS |  | 40 | 46.2 |
| 1 | Forand | | 2011 | NRCT | DSM-IV-TR | CBT | 249 | 249 | BDI-II |  | 34.9 | 36.7 |
| 1 | Forkmann | | 2014 | NRCT | ICD-10 | CBT | 44 | 44 | BDI |  | 36.4 | 36.4 |
| 1 | Forman | | 2007 | RCT | DSM-IV-TR | CBT | 74 | 74 | BDI-II |  | 19.8 | 27.87 |
| 1 | Frank | | 2011 | NRCT | DSM-IV | ESCITALOPRAM | 158 | 158 | HDRS |  | 28.3 | 39.2 |
| 1 | Freeman | | 1993 | NRCT | DSM-III-R | MIRTAZAPINE | 35 | 35 | HDRS |  | 46 | 40.4 |
| 1 | Fresco | | 2007 | NRCT | DSM-IV | CBT | 68 | 46 | HDRS | BDI-II | 38 | 37 |
| 1 | Fu | | 2008 | NRCT | DSM-IV | CBT | 16 | 16 | HDRS | BDI | 23.08 | 40 |
| 1 | Fujino | | 2015 | NRCT | DSM-IV | CBT | 10 | 10 | BDI-II |  | 30 | 33.5 |
| 1 | Funaki | | 2016 | RCT | DSM-IV | MIRTAZAPINE | 194 | 194 | HDRS |  | 47.94 | 39.4 |
| 1 | Gambi | | 2005 | NRCT | DSM-IV | MIRTAZAPINE | 17 | 17 | HDRS |  | 41.18 | 41.3 |
| 1 | Gasto | | 2003 | NRCT | DSM-IV | VENLAFAXINE | 34 | 34 | HDRS | MADRS | 35.29 | 71.44 |
| 1 | Gentil | | 2000 | RCT | DSM-IV | VENLAFAXINE | 57 | 57 | HDRS | MADRS | 20 | 37.9 |
| 1 | Gerra | | 2014 | RCT | DSM-IV-TR | ESCITALOPRAM | 82 | 82 | HDRS | MADRS | 26.8 | 40.2 |
| 1 | Geschwind | | 2019 | NRCT | DSM-IV | CBT | 49 | 49 | QUIDS |  | 36 | 40.8 |
| 1 | Gex-Farby | | 2004 | RCT | ICD-10 | VENLAFAXINE | 35 | 35 | MADRS |  | 40 | 44 |
| 1 | Gilaberte | | 2013 | NRCT | DSM-IV-TR | ESCITALOPRAM | 284 | 284 | HDRS |  | 30.6 | 47.4 |
| 1 | Godlewska | | 2018 | NRCT | DSM-IV | ESCITALOPRAM | 39 | 32 | HDRS | BDI | 43.75 | 28.5 |
| 1 | Godlewska | | 2015 | NRCT | DSM-IV-TR | ESCITALOPRAM | 33 | 33 | HDRS | BDI | 42.42 | 29.9 |
| 1 | Godlewska | | 2016 | NRCT | DSM-IV | ESCITALOPRAM | 35 | 35 | HDRS | BDI | 42.86 | 30.05 |
| 1 | Godlewska | | 2012 | NRCT | DSM-IV | ESCITALOPRAM | 21 | 21 | HDRS | BDI | 42.86 | 32 |
| 1 | Goldapple | | 2004 | NRCT | DSM-IV | CBT | 14 | 14 | HDRS |  | 35.29 | 41 |
| 1 | Goodkind | | 2016 | NRCT | DSM | CBT | 55 | 55 | BDI-II |  | 36 | 69.4 |
| 1 | Goodnick | | 1999 | NRCT | DSM-IV | MIRTAZAPINE | 10 | 10 | HDRS | BDI | 40 | 42.4 |
| 1 | Gorenstien | | 2002 | NRCT | ICD-10 | VENLAFAXINE | 12 | 11 | HDRS | BDI + MADRS | 28.57 | 35.8 |
| 1 | Grasmader | | 2005 | NRCT | ICD-10 | MIRTAZAPINE | 65 | 65 | HDRS |  | 43.08 | 49.2 |
| 1 | Grigoriadis | | 2003 | NRCT | DSM-IV | VENLAFAXINE | 44 | 44 | HDRS |  | 0 | 32.4 |
| 1 | Grossman | | 2004 | NRCT | DSM-IV | VENLAFAXINE | 19 | 19 | HDRS |  | 57.89 | N/A |
| 1 | Groves | | 2015 | NRCT | DSM-IV-TR | CBT | 25 | 25 | MADRS |  | 48 | 35 |
| 1 | Guelfi | | 2001 | RCT | DSM-IV | MIRTAZAPINE | 78 | 78 | HDRS | MADRS | 37.2 | 45.9 |
| 1 | Guelfi B | | 2001 | RCT | DSM-IV | VENLAFAXINE | 79 | 79 | HDRS | MADRS | 31.6 | 44.5 |
| 1 | Gunning | | 2010 | RCT | DSM-IV-TR | ESCITALOPRAM | 42 | 42 | HDRS |  | 40.7 | 70.4 |
| 1 | Gunning | | 2009 | NRCT | DSM-IV-TR | ESCITALOPRAM | 41 | 41 | HDRS |  | N/A | 70.5 |
| 1 | Guo | | 2015 | RCT | DSM-IV | MIRTAZAPINE | 31 | 31 | HDRS |  | 40.7 | 43.5 |
| 1 | Gupta | | 2016 | NRCT | DSM-IV | MIRTAZAPINE | 30 | 30 | HDRS |  | 50 | 29.6 |
| 1 | Halaris | | 2015 | NRCT | DSM-IV | ESCITALOPRAM | 30 | 30 | HDRS | BDI | 23 | 37.1 |
| 1 | Harkness | | 2012 | RCT | DSM-IV | CBT | 70 | 47 | HDRS |  | N/A | 40.99 |
| 1 | Harte | | 2016 | NRCT | DSM-IV | CBT | 60 | 60 | BDI-II |  | 41.45 | 32.05 |
| 1 | Hauksson | | 2017 | NRCT | DSM | CBT | 59 | 39 | BDI-II |  | 33.9 | 45.39 |
| 1 | He | | 2018 | RCT | DSM-IV | CBT | 44 | 38 | HDRS |  | 50 | 30.3 |
| 1 | Heller | | 2013 | NRCT | DSM-IV | VENLAFAXINE | 29 | 29 | HDRS |  | 58.33 | 29.67 |
| 1 | Hensley | | 2009 | NRCT | DSM-IV-TR | ESCITALOPRAM | 30 | 30 | HDRS | MADRS | 10 | 45.67 |
| 1 | Herrera-Guzman | | 2009 | NRCT | DSM-IV | ESCITALOPRAM | 36 | 36 | HDRS |  | 13.89 | 32.91 |
| 1 | Hewett | | 2009 | RCT | DSM-IV | VENLAFAXINE | 187 | 187 | MADRS |  | 32 | 42.7 |
| 1 | Hewett | | 2010 | RCT | DSM-IV | VENLAFAXINE | 198 | 198 | MADRS |  | 32 | 44.1 |
| 1 | Higuhi | | 2016 | RCT | DSM-IV | VENLAFAXINE | 354 | 332 | HDRS |  | N/A | 38.35 |
| 1 | Hirsch | | 2000 | NRCT | DSM-III-R | CBT | 37 | 25 | BDI |  | 36 | 36 |
| 1 | Ho | | 2015 | NRCT | DSM-IV | ESCITALOPRAM | 26 | 26 | HDRS |  | 100 | 24.7 |
| 1 | Hofmann | | 2014 | RCT | DSM-IV | CBT | 21 | 21 | BDI-II |  | 23.81 | 40.67 |
| 1 | Hopkins | | 2013 | RCT | DSM-IV | VENLAFAXINE | N/A | N/A | HDRS |  | N/A | N/A |
| 1 | Horing | | 2002 | NRCT | DSM-IV | VENLAFAXINE | 17 | 17 | HDRS |  | 70.59 | 43 |
| 1 | Hortsmann | | 2009 | RCT | DSM-IV | MIRTAZAPINE | 55 | 55 | HDRS |  | 55 | 51.9 |
| 1 | Hsiao | | 2006 | NRCT | DSM-IV | VENLAFAXINE | 34 | 34 | HDRS |  | 26.47 | 35.1 |
| 1 | Hsyoerg | | 1996 | RCT | DSM-III | MIRTAZAPINE | 56 | 56 | HDRS | MADRS | 21.43 | 70 |
| 1 | Hu | | 2016 | RCT | DSM-IV | ESCITALOPRAM | 15 | 14 | MADRS |  | 28.6 | 41 |
| 1 | Huang | | 2016 | NRCT | DSM-5 | ESCITALOPRAM | 53 | 53 | BDI-II |  | 37.74 | 50.19 |
| 1 | Huang B | | 2016 | NRCT | DSM-5 | VENLAFAXINE | 9 | 9 | BDI-II |  | 37.74 | 50.19 |
| 1 | Huang C | | 2016 | NRCT | DSM-5 | MIRTAZAPINE | 9 | 9 | BDI-II |  | 37.74 | 50.19 |
| 1 | Huezo-Diaz | | 2009 | NRCT | DSM-IV/ICD-10 | ESCITALOPRAM | 450 | 450 | HDRS | BDI + MADRS | 39 | 42.93 |
| 1 | Hung | | 2016 | NRCT | DSM-IV | ESCITALOPRAM | 11 | 11 | HDRS |  | 32.14 | 45.62 |
| 1 | Hung | | 2012 | NRCT | DSM-IV-TR | VENLAFAXINE | 135 | 72 | HDRS |  | 25.19 | 30.2 |
| 1 | Hung | | 2015 | NRCT | DSM-IV-TR | VENLAFAXINE | 155 | 85 | HDRS |  | 31.6 | 30.3 |
| 1 | Hunter | | 2009 | RCT | DSM-IV | VENLAFAXINE | 58 | 58 | HDRS |  | N/A | 40.37 |
| 1 | Hunter | | 2010 | RCT | DSM-IV | VENLAFAXINE | 24 | 24 | HDRS |  | 40.28 | 41.7 |
| 1 | Hunter | | 2012 | RCT | DSM-IV | VENLAFAXINE | 47 | 47 | HDRS |  | 38.3 | 42.4 |
| 1 | Hunter | | 2006 | RCT | DSM-IV | VENLAFAXINE | 27 | 27 | HDRS |  | N/A | 42.6 |
| 1 | Hwang | | 2015 | RCT | DSM-IV | CBT | 23 | 23 | HDRS |  | 26 | 45.7 |
| 1 | Iglesias | | 2009 | NRCT | DSM-IV | VENLAFAXINE | 45 | 43 | HDRS |  | 0 | 50.82 |
| 1 | Iina | | 2009 | NRCT | ICD-10 | VENLAFAXINE | 30 | 30 | BDI | HDRS | 23.33 | 35.3 |
| 1 | Ionescu | | 2016 | RCT | DSM-IV | ESCITALOPRAM | 177 | 177 | HDRS |  | N/A | N/A |
| 1 | Iosifescu | | 2009 | NRCT | DSM-IV | VENLAFAXINE | 82 | 82 | HDRS |  | 52.4 | 35.9 |
| 1 | Ivanets | | 2016 | NRCT | ICD-10 | VENLAFAXINE | 62 | 62 | MADRS |  | 33.87 | 39.4 |
| 1 | Jacobsen | | 2000 | NRCT | DSM-III-R | CBT | 50 | 50 | BDI | HDRS | 24 | 39.2 |
| 1 | Jacobsen | | 1993 | NRCT | DSM-III | CBT | 20 | 14 | BDI |  | 0 | N/A |
| 1 | Jaworska | | 2014 | NRCT | DSM | ESCITALOPRAM | 53 | 53 | MADRS | HDRS | 43.4 | 26.75 |
| 1 | Jeon | | 2014 | RCT | DSM-IV-TR | ESCITALOPRAM | 81 | 81 | HDRS |  | 17.3 | 57.1 |
| 1 | Jeong | | 2015 | RCT | DSM-IV-TR | ESCITALOPRAM | 158 | 100 | HDRS | MADRS | 29.1 | 48.1 |
| 1 | Jgl | | 2018 | RCT | DSM-IV | CBT | N/A | N/A | HDRS |  | N/A | N/A |
| 1 | Jiang | | 2017 | NRCT | DSM-IV-TR | ESCITALOPRAM | 285 | 191 | MADRS | HDRS | 43.9 | 40.5 |
| 1 | Joffe | | 1998 | NRCT | DSM-IV | VENLAFAXINE | 880 | 880 | CGI |  | 33.86 | 41.9 |
| 1 | Joffe | | 2001 | NRCT | DSM-IV | MIRTAZAPINE | 22 | 22 | HDRS | BDI | 0 | 51 |
| 1 | Johnson | | 2018 | RCT | DSM-IV | CBT | 38 | 38 | PHQ9 |  | 39.19 | 42 |
| 1 | Judd | | 2001 | NRCT | DSM-IV | VENLAFAXINE | 19 | 19 | BDI |  | N/A | 37.5 |
| 1 | Kaichi | | 2016 | NRCT | DSM | ESCITALOPRAM | 56 | 56 | HDRS |  | 50.94 | 42.2 |
| 1 | Kalin | | 1997 | NRCT | DSM-IV | VENLAFAXINE | 20 | 20 | HDRS | BDI | N/A | N/A |
| 1 | Kang | | 2010 | NRCT | DSM-IV | MIRTAZAPINE | 243 | 243 | HDRS |  | 25.4 | 50.39 |
| 1 | Kang | | 2009 | NRCT | DSM-IV | VENLAFAXINE | 53 | 37 | HDRS |  | 22.6 | 47.2 |
| 1 | Kang B | | 2009 | NRCT | DSM-IV | MIRTAZAPINE | 73 | 51 | HDRS |  | 23.3 | 46.5 |
| 1 | Kang B | | 2007 | NRCT | DSM-IV | MIRTAZAPINE | 101 | 101 | HDRS |  | 29 | 50.01 |
| 1 | Kaplan | | 2002 | NRCT | DSM-IV | VENLAFAXINE | 73 | 73 | HDRS |  | 26.02 | 38.6 |
| 1 | Karp | | 2008 | NRCT | DSM-IV | ESCITALOPRAM | 216 | 216 | HDRS |  | N/A | N/A |
| 1 | Kasper | | 2012 | RCT | DSM-IV-TR | ESCITALOPRAM | 140 | 140 | MADRS |  | 21.4 | 41.6 |
| 1 | Kasper | | 2005 | RCT | DSM-IV | ESCITALOPRAM | 173 | 144 | MADRS |  | 25 | 75 |
| 1 | Kato | | 2017 | RCT | DSM-IV | MIRTAZAPINE | 77 | 60 | HDRS |  | 52.2 | 48.5 |
| 1 | Keller | | 2012 | NRCT | DSM-IV | VENLAFAXINE | 781 | 781 | HDRS |  | 35 | 39.6 |
| 1 | Kennedy | | 2007 | RCT | DSM-IV-TR | CBT | 17 | 14 | HDRS |  | 35.71 | 30 |
| 1 | Kennedy | | 2000 | NRCT | DSM-IV | VENLAFAXINE | 36 | 36 | HDRS |  | 38.89 | 43.7 |
| 1 | Keri | | 2014 | NRCT | DSM-IV | CBT | 44 | 44 | HDRS |  | 34.09 | 25.6 |
| 1 | Keri B | | 2014 | NRCT | DSM-IV | CBT | 50 | 43 | HDRS |  | 38 | 22.6 |
| 1 | Kessler | | 2018 | RCT | ICD-10 | MIRTAZAPINE | 241 | 214 | BDI-II | HDRS | 30.3 | 50.4 |
| 1 | Khan | | 2007 | RCT | DSM-IV | ESCITALOPRAM | 137 | 91 | HDRS | MADRS | 40.9 | 41.8 |
| 1 | Kim | | 2014 | NRCT | DSM-IV | ESCITALOPRAM | 245 | 245 | HDRS |  | 18.25 | 50.72 |
| 1 | Kim | | 2011 | RCT | DSM-IV | MIRTAZAPINE | 29 | 29 | HDRS |  | 24.1 | 42.6 |
| 1 | King | | 2014 | RCT | ICD-10 | CBT | 129 | 129 | BDI |  | 22 | 37 |
| 1 | Kirchheiner | | 2007 | NRCT | DSM | VENLAFAXINE | 39 | 39 | HDRS |  | 38 | 46 |
| 1 | Kirchheiner B | | 2007 | NRCT | DSM | MIRTAZAPINE | 58 | 58 | HDRS |  | 34 | 45 |
| 1 | Kishi | | 2017 | RCT | DSM-IV | ESCITALOPRAM | 43 | 19 | HDRS |  | 58.1 | 38.9 |
| 1 | Kishon | | 2015 | NRCT | DSM-IV | CBT | 20 | 20 | HDRS | BDI-II | 55 | 39.15 |
| 1 | Kocer | | 2015 | NRCT | DSM-IV | ESCITALOPRAM | 27 | 27 | BDI |  | 18.52 | 33.1 |
| 1 | Kocer B | | 2015 | NRCT | DSM-IV | VENLAFAXINE | 14 | 14 | BDI |  | 28.57 | 36 |
| 1 | Koeing | | 2016 | RCT | DSM-IV | CBT | 67 | 48 | BDI |  | 35.8 | 52.9 |
| 1 | Kohler | | 2013 | NRCT | ICD-10 | CBT + MIRTAZAPINE | 138 | 105 | HDRS | BDI | 44.76 | 54.67 |
| 1 | Kok | | 2007 | RCT | DSM-IV | VENLAFAXINE | 40 | 35 | MADRS | HDRS | 32.5 | 71.6 |
| 1 | Komulainen | | 2018 | NRCT | DSM-IV | ESCITALOPRAM | 25 | 25 | MADRS | BDI | 32 | N/A |
| 1 | Konarski | | 2009 | NRCT | DSM-IV | CBT | 12 | 12 | HDRS |  | 44.3 | 29.45 |
| 1 | Konarski B | | 2009 | NRCT | DSM-IV | VENLAFAXINE | 12 | 12 | HDRS |  | 27.8 | 38.95 |
| 1 | Kontantinidis | | 2002 | NRCT | DSM-IV | MIRTAZAPINE | 27 | 27 | HDRS |  | 15 | 50.5 |
| 1 | Kopecek | | 2007 | NRCT | ICD-10 | VENLAFAXINE | 22 | 22 | BDI |  | 50 | 50.5 |
| 1 | Koutouvidis | | 1999 | NRCT | ICD-10 | MIRTAZAPINE | 11 | 11 | HDRS |  | 54.5 | 31.8 |
| 1 | Kring | | 2007 | NRCT | DSM-IV-TR | CBT | 36 | 36 | BDI |  | 35 | 35.75 |
| 1 | Kroger | | 2015 | RCT | DSM-IV | CBT | 13 | 13 | BDI |  | 46 | 41.85 |
| 1 | Kuang | | 2016 | NRCT | DSM-IV | ESCITALOPRAM | 30 | 30 | HDRS |  | N/A | N/A |
| 1 | Kuhlan | | 2018 | NRCT | DSM-V | VENLAFAXINE | 23 | 23 | MADRS |  | N/A | N/A |
| 1 | Kundermann | | 2009 | NRCT | DSM-IV | CBT | 18 | 18 | HDRS | BDI | 61.11 | 36.8 |
| 1 | Kundermann | | 2015 | NRCT | DSM-IV | CBT | 10 | 10 | HDRS | BDI | 70 | 37.4 |
| 1 | Kuyken | | 2008 | NRCT | DSM-IV | CBT | 69 | 69 | BDI-II |  | 29 | 34.84 |
| 1 | Laakmann | | 2003 | NRCT | DSM-IV | MIRTAZAPINE | 12 | 12 | HDRS |  | 33.33 | 39.92 |
| 1 | Ladd | | 2005 | NRCT | DSM-IV | VENLAFAXINE | 16 | 16 | HDRS |  | 0 | 45.9 |
| 1 | Ladea | | 2013 | NRCT | DSM-IV-TR | ESCITALOPRAM | 20 | 20 | MADRS |  | N/A | 36.6 |
| 1 | Laidlaw | | 2008 | RCT | DSM-IV | CBT | 21 | 20 | BDI-II | HDRS | 40 | 74 |
| 1 | Lam | | 2013 | RCT | DSM-IV | ESCITALOPRAM | 51 | 51 | MADRS |  | 47 | 44.2 |
| 1 | Lancon | | 2006 | NRCT | DSM-IV | ESCITALOPRAM | 67 | 67 | MADRS |  | 38.8 | 44 |
| 1 | Lanzenberger | | 2012 | NRCT | DSM-IV | ESCITALOPRAM | 22 | 22 | HDRS |  | 31.58 | 42.3 |
| 1 | Laux | | 2013 | NRCT | ICD-10 | ESCITALOPRAM | 2911 | 2718 | MADRS | HDRS | 32 | 47.3 |
| 1 | Lavergne | | 2005 | NRCT | DSM-IV | MIRTAZAPINE | 4771 | 4771 | MADRS |  | 32 | 45 |
| 1 | Learned | | 2012 | RCT | DSM-IV-TR | VENLAFAXINE | 133 | 84 | HDRS | MADRS | 61 | 43 |
| 1 | Lee | | 2015 | NRCT | DSM-IV | ESCITALOPRAM | 32 | 32 | BDI |  | 21.88 | 40.2 |
| 1 | Lee | | 2009 | NRCT | DSM-IV | MIRTAZAPINE | 314 | 314 | HDRS |  | 25 | 49.83 |
| 1 | Lee | | 2010 | NRCT | DSM-IV | VENLAFAXINE | 101 | 101 | BDI | HDRS + MADRS | 40 | 44.6 |
| 1 | Lemoine | | 2007 | RCT | DSM-IV | VENLAFAXINE | 167 | 131 | HDRS |  | 32.9 | 39.6 |
| 1 | Lenderking | | 1999 | NRCT | DSM-III-R | VENLAFAXINE | 600 | 600 | HDRS | MADRS | 32.7 | 39.05 |
| 1 | Lenze | | 2015 | RCT | DSM-IV | VENLAFAXINE | 392 | 90 | MADRS |  | 35.99 | 60.01 |
| 1 | Lepola | | 2003 | NRCT | DSM-IV | ESCITALOPRAM | 155 | 155 | MADRS |  | 25.2 | 43 |
| 1 | Leutcher | | 2009 | RCT | DSM-IV | ESCITALOPRAM | 73 | 73 | HDRS |  | 34.2 | 42.7 |
| 1 | Leutcher | | 2002 | RCT | DSM-IV | VENLAFAXINE | 27 | 27 | HDRS |  | N/A | 41.75 |
| 1 | Lewis | | 2012 | NRCT | DSM-IV | CBT | 173 | 173 | BDI |  | 33.53 | 27.94 |
| 1 | Li | | 2018 | NRCT | DSM-IV | CBT | 20 | 20 | HDRS |  | 50 | 27.3 |
| 1 | Li | | 2013 | NRCT | DSM-IV | ESCITALOPRAM | 15 | 15 | MADRS |  | 100 | 32.35 |
| 1 | Li B | | 2017 | NRCT | DSM-IV-TR | VENLAFAXINE | 120 | 120 | HDRS |  | 37.5 | 31.5 |
| 1 | Li C | | 2017 | NRCT | DSM-IV-TR | VENLAFAXINE | 64 | 64 | HDRS |  | 17.2 | 31.6 |
| 1 | Li A | | 2017 | NRCT | DSM-IV-TR | VENLAFAXINE | 8 | 8 | HDRS |  | N/A | N/A |
| 1 | Li B | | 2018 | RCT | DSM-5 | ESCITALOPRAM | 119 | 106 | HDRS |  | 0 | 49.93 |
| 1 | Li B | | 2013 | RCT | DSM-IV | VENLAFAXINE | 46 | 46 | HDRS |  | 47.83 | 42.6 |
| 1 | Li C | | 2013 | NRCT | DSM-IV-TR | VENLAFAXINE | 61 | 61 | HDRS |  | 17.55 | 32.65 |
| 1 | Liang | | 2012 | NRCT | DSM-IV | VENLAFAXINE | 45 | 45 | HDRS |  | 100 | 23.45 |
| 1 | Lin | | 2013 | NRCT | DSM-IV | ESCITALOPRAM | 114 | 114 | HDRS | MADRS | 17.50 | 42.3 |
| 1 | Lin | | 2008 | NRCT | DSM-IV | VENLAFAXINE | 122 | 122 | CGI |  | 26.2 | 44.4 |
| 1 | Lin | | 2012 | NRCT | DSM-IV-TR | VENLAFAXINE | 70 | 70 | HDRS |  | 28.57 | 32.1 |
| 1 | Lin | | 2011 | NRCT | DSM-IV-TR | ESCITALOPRAM | 100 | 100 | HDRS |  | 19 | 42 |
| 1 | Lisiecka | | 2011 | NRCT | DSM-IV | MIRTAZAPINE | 12 | 10 | HDRS |  | 70 | 37.7 |
| 1 | Lisiecka B | | 2011 | NRCT | DSM-IV | VENLAFAXINE | 14 | 13 | HDRS |  | 61.54 | 38.9 |
| 1 | Little | | 2005 | NRCT | DSM-IV | VENLAFAXINE | 20 | 20 | HDRS | BDI | 50 | 43.6 |
| 1 | Little | | 1999 | NRCT | DSM-IV | VENLAFAXINE | 9 | 9 | HDRS |  | 55.56 | 48 |
| 1 | Lobelllo | | 2010 | NRCT | DSM-IV | VENLAFAXINE | 269 | 263 | HDRS |  | 25 | 44 |
| 1 | Lopez | | 2011 | NRCT | DSM-IV | CBT | 40 | 40 | BDI |  | 10 | 46 |
| 1 | Lopez | | 2015 | NRCT | DSM-IV | CBT | 83 | 83 | QUIDS |  | 15.7 | 42.8 |
| 1 | Lopez | | 2014 | RCT | DSM-IV | CBT | 29 | 20 | BDI-II |  | 17.2 | 33.41 |
| 1 | Lopez | | 2016 | NRCT | DSM-IV | ESCITALOPRAM | 12 | 12 | HDRS |  | 41.7 | 49.6 |
| 1 | Lorenzo-Lucas | | 2017 | RCT | DSM-IV | CBT | 208 | 208 | SCL-90 |  | 41 | 36.63 |
| 1 | Lou | | 2013 | NRCT | DSM-IV | VENLAFAXINE | N/A | N/A | HDRS |  | 33.33 | 36 |
| 1 | Luty | | 2007 | RCT | DSM-IV | CBT | 86 | 76 | BDI-II | HDRS + MADRS | 31 | 35.2 |
| 1 | Lyketsos | | 2011 | NRCT | DSM-IV | ESCITALOPRAM | 405 | 405 | MADRS |  | 22.5 | 74 |
| 1 | Lyoo | | 2012 | RCT | DSM-IV | ESCITALOPRAM | 27 | 27 | HDRS | MADRS | 0 | 47.5 |
| 1 | Machado | | 2008 | NRCT | DSM-IV | VENLAFAXINE | N/A | N/A | HDRS | MADRS | N/A | N/A |
| 1 | Macrea | | 2015 | NRCT | ICD-10 | CBT | 38 | 38 | EPDS |  | 0 | 30 |
| 1 | Maity | | 2014 | RCT | DSM-IV-TR | ESCITALOPRAM | 43 | 39 | HDRS |  | 64.1 | 40 |
| 1 | Manning | | 2015 | NRCT | DSM-IV | ESCITALOPRAM | 100 | 100 | HDRS |  | 46.5 | 71.63 |
| 1 | Mao | | 2008 | RCT | DSM-IV | ESCITALOPRAM | 118 | 108 | HDRS | MADRS | 53 | 37.1 |
| 1 | Marcus | | 2008 | RCT | DSM-IV-TR | ESCITALOPRAM | N/A | N/A | MADRS |  | 32.6 | 44.4 |
| 1 | Marcus B | | 2008 | RCT | DSM-IV-TR | VENLAFAXINE | N/A | N/A | MADRS |  | 32.6 | 44.4 |
| 1 | Maron | | 2009 | NRCT | DSM-IV | ESCITALOPRAM | 126 | 126 | MADRS | HDRS + BDI | 33.05 | 31.35 |
| 1 | Marquett | | 2013 | NRCT | DSM-IV | CBT | 60 | 60 | BDI-II |  | 38 | 69.7 |
| 1 | Marshall | | 2008 | NRCT | DSM-IV-TR | CBT | 37 | 37 | HDRS | BDI-II | 31 | N/A |
| 1 | Marshe | | 2017 | NRCT | DSM-IV | VENLAFAXINE | 350 | 350 | MADRS |  | 36.3 | 68.6 |
| 1 | Martin | | 2001 | NRCT | DSM-IV | VENLAFAXINE | 15 | 15 | HDRS | BDI | 26.67 | 39.4 |
| 1 | Marttila | | 1995 | RCT | DSM-III | MIRTAZAPINE | 83 | 83 | HDRS | MADRS | 48.19 | 41.3 |
| 1 | Matrisciano | | 2009 | NRCT | DSM-IV-TR | ESCITALOPRAM | 7 | 7 | HDRS |  | 42.86 | 41.3 |
| 1 | Matrisciano B | | 2009 | NRCT | DSM-IV-TR | VENLAFAXINE | 7 | 7 | HDRS |  | 57.14 | 43.7 |
| 1 | Mazeh | | 2007 | RCT | DSM-IV | VENLAFAXINE | 15 | 15 | HDRS |  | 46.67 | 74.1 |
| 1 | McBride | | 2006 | RCT | DSM-IV | CBT | 28 | 28 | BDI-II | HDRS | 27.59 | 41 |
| 1 | McBride | | 2007 | NRCT | DSM-IV | CBT | 21 | 21 | BDI-II |  | 30.95 | 40.71 |
| 1 | McEvoy | | 2014 | NRCT | DSM-IV | CBT | 84 | 50 | BDI-II |  | 32 | 35.4 |
| 1 | McGrath | | 2013 | RCT | DSM-IV-TR | CBT | 21 | 21 | HDRS |  | 45.8 | 43.95 |
| 1 | McGrath B | | 2013 | RCT | DSM-IV-TR | ESCITALOPRAM | 18 | 18 | HDRS | BDI | 37.5 | 40.05 |
| 1 | Mehtonen | | 2000 | RCT | DSM-IV | VENLAFAXINE | 75 | 75 | HDRS | MADRS | 34.67 | 44.1 |
| 1 | Mendez | | 2012 | NRCT | DSM-IV | MIRTAZAPINE | 20 | 20 | HDRS |  | 40 | 47.55 |
| 1 | Merino | | 2000 | NRCT | ICD-10 | VENLAFAXINE | 20 | 20 | MADRS |  | 35 | 46.08 |
| 1 | Merrill | | 2003 | NRCT | DSM-III-R | CBT | 192 | 192 | BDI | HDRS | 30 | 32.7 |
| 1 | Metts | | 2018 | NRCT | DSM-IV | CBT | 31 | 31 | HDRS | BDI | 35.48 | 36.55 |
| 1 | Midi | | 2012 | NRCT | DSM-IV | ESCITALOPRAM | 56 | 56 | MADRS |  | 0 | 40.82 |
| 1 | Milgrom | | 2015 | RCT | DSM-IV | CBT | 15 | 15 | BDI-II |  | 0 | 28.5 |
| 1 | Miller | | 2013 | NRCT | DSM-IV | ESCITALOPRAM | 24 | 24 | HDRS |  | 29.75 | 34.95 |
| 1 | Miller B | | 2013 | NRCT | DSM-IV | ESCITALOPRAM | 17 | 17 | HDRS | BDI | 41.2 | 35.65 |
| 1 | Mischoulon | | 2014 | RCT | DSM-IV | ESCITALOPRAM | 65 | 30 | HDRS |  | 50 | 45 |
| 1 | Molina | | 2017 | RCT | DSM | CBT | 91 | 91 | HDRS |  | 24.18 | N/A |
| 1 | Moller | | 2007 | NRCT | ICD-10 | ESCITALOPRAM | 11969 | 11969 | CGI |  | 29.8 | 51.9 |
| 1 | Montane | | 2018 | NRCT | DSM-IV | VENLAFAXINE | 54 | 54 | HDRS |  | 33.3 | 40 |
| 1 | Montgomery | | 2006 | RCT | DSM-IV | ESCITALOPRAM | 249 | 244 | MADRS |  | 29 | 43 |
| 1 | Montgomery | | 1998 | RCT | DSM-III | MIRTAZAPINE | 74 | 74 | HDRS |  | 37.8 | 41.6 |
| 1 | Montgomery | | 2004 | NRCT | DSM-III-R | VENLAFAXINE | 109 | 109 | HDRS |  | 29 | 43.8 |
| 1 | Montgomery B | | 2004 | RCT | DSM-IV | ESCITALOPRAM | 148 | 146 | MADRS |  | 27 | 49 |
| 1 | Montgomery B | | 2006 | RCT | DSM-IV | VENLAFAXINE | 246 | 243 | MADRS |  | 41 | 37.5 |
| 1 | Montgomery C | | 2004 | RCT | DSM-IV | VENLAFAXINE | 145 | 143 | MADRS |  | 29 | 47 |
| 1 | Moore | | 2005 | RCT | DSM-IV | ESCITALOPRAM | 138 | 132 | MADRS |  | 28.3 | 44.1 |
| 1 | Moreira | | 2015 | RCT | DSM-IV | CBT | 28 | 28 | HDRS |  | 25 | 24.46 |
| 1 | Morimoto | | 2012 | NRCT | DSM-IV-TR | ESCITALOPRAM | 65 | 65 | HDRS |  | N/A | 70.25 |
| 1 | Mrazek | | 2014 | NRCT | DSM-IV | ESCITALOPRAM | N/A | N/A | QUIDS |  | N/A | 39.9 |
| 1 | Muhlbacher | | 2006 | NRCT | DSM-IV | MIRTAZAPINE | 80 | 80 | HDRS |  | 20 | 48.9 |
| 1 | Mullin | | 1996 | RCT | DSM-III | MIRTAZAPINE | 79 | 79 | HDRS | MADRS | 25.32 | 45.4 |
| 1 | Murphy | | 2003 | RCT | DSM-IV | MIRTAZAPINE | 124 | 124 | HDRS |  | 50.81 | 76.28 |
| 1 | Murphy | | 1995 | NRCT | DSM-III | CBT | 11 | 11 | BDI |  | 27.27 | 39.8 |
| 1 | Murphy | | 2007 | NRCT | DSM-IV | ESCITALOPRAM | 51 | 51 | HDRS |  | 43 | 70 |
| 1 | Murphy B | | 2003 | RCT | DSM-IV | MIRTAZAPINE | 121 | 121 | HDRS |  | 51.75 | 71.43 |
| 1 | Myung | | 2015 | RCT | DSM-IV-TR | ESCITALOPRAM | 184 | 184 | HDRS |  | N/A | N/A |
| 1 | Myung | | 2012 | NRCT | DSM-IV-TR | VENLAFAXINE | 18 | 18 | HDRS |  | 24 | 64 |
| 1 | Myung B | | 2015 | RCT | DSM-IV-TR | ESCITALOPRAM | 148 | 148 | HDRS |  | N/A | N/A |
| 1 | Myung B | | 2012 | NRCT | DSM-IV-TR | MIRTAZAPINE | 48 | 48 | HDRS |  | 24 | 64 |
| 1 | Nago | | 2013 | NRCT | DSM-IV | MIRTAZAPINE | 22 | 22 | HDRS | MADRS | 81.8 | 43.6 |
| 1 | Nakagawa | | 2017 | RCT | DSM-IV | CBT | 40 | 40 | BDI-II | HDRS | 62.5 | 39.5 |
| 1 | Nam | | 2017 | NRCT | DSM-IV | ESCITALOPRAM | 15 | 15 | BDI |  | N/A | 23.9 |
| 1 | Nehama | | 2014 | NRCT | DSM-IV-TR | ESCITALOPRAM | 40 | 40 | MADRS |  | 52.5 | 45.1 |
| 1 | Nemeroff | | 2007 | RCT | DSM-IV | VENLAFAXINE | 102 | 102 | HDRS | MADRS | 35 | 40.1 |
| 1 | Ng | | 2012 | NRCT | DSM-IV-TR | VENLAFAXINE | 44 | 44 | HDRS |  | 35 | 42.3 |
| 1 | Ng B | | 2012 | NRCT | DSM-IV-TR | ESCITALOPRAM | 62 | 62 | HDRS |  | 43 | 40.23 |
| 1 | Nordahl | | 2009 | NRCT | ICD-10 | CBT | 13 | 13 | BDI |  | 38.46 | 34.9 |
| 1 | Nothdurfter | | 2014 | NRCT | DSM-IV | ESCITALOPRAM | 20 | 20 | HDRS |  | 52.5 | 42.05 |
| 1 | Olie | | 2007 | NRCT | DSM-IV-TR | ESCITALOPRAM | 774 | 649 | MADRS |  | 30 | 44.5 |
| 1 | Olie | | 2010 | RCT | DSM-IV-TR | VENLAFAXINE | 91 | 91 | MADRS |  | 39.6 | 42.7 |
| 1 | Organista | | 1994 | NRCT | DSM-III-R | CBT | 171 | 74 | BDI |  | 25 | 49.7 |
| 1 | Osvath | | 2007 | NRCT | DSM-IV | MIRTAZAPINE | 102 | 102 | HDRS | BDI | 43.1 | 42.19 |
| 1 | Otte | | 2010 | RCT | DSM-IV | ESCITALOPRAM | 13 | 13 | HDRS | BDI | 36 | 34.5 |
| 1 | Ou | | 2011 | RCT | DSM-IV-TR | ESCITALOPRAM | 115 | 115 | HDRS |  | 41.7 | 36.7 |
| 1 | Owens | | 2008 | NRCT | DSM-IV | VENLAFAXINE | 41 | 41 | MADRS |  | 36 | N/A |
| 1 | Ozbey | | 2017 | NRCT | DSM-IV | VENLAFAXINE | 94 | 52 | HDRS |  | 17.31 | 38.1 |
| 1 | Ozdemir | | 2015 | NRCT | DSM-IV | VENLAFAXINE | 25 | 25 | HDRS | BDI | 46 | 35.76 |
| 1 | Ozsoy | | 2016 | NRCT | DSM-IV | ESCITALOPRAM | 9 | 9 | HDRS |  | 12.5 | 32.03 |
| 1 | Ozsoy B | | 2016 | NRCT | DSM-IV | VENLAFAXINE | 7 | 7 | HDRS |  | 12.5 | 32.03 |
| 1 | Panteleeva | | 2016 | NRCT | ICD-10 | VENLAFAXINE | 32 | 32 | HDRS |  | 53.13 | 31.75 |
| 1 | Papakostas | | 2010 | NRCT | DSM-IV | ESCITALOPRAM | 17 | 17 | HDRS |  | 41.18 | 42.1 |
| 1 | Papakostas | | 2014 | RCT | DSM-IV | ESCITALOPRAM | 68 | 68 | HDRS |  | 28 | 44.2 |
| 1 | Park | | 2015 | NRCT | DSM-IV | ESCITALOPRAM | 51 | 51 | HDRS | MADRS | 17.65 | 47.95 |
| 1 | Parker | | 2013 | RCT | DSM-IV | CBT | 11 | 11 | HDRS | BDI | 27.3 | 48 |
| 1 | Parker | | 2013 | NRCT | DSM-IV | CBT | 11 | 11 | HDRS |  | N/A | N/A |
| 1 | Paslakis | | 2010 | NRCT | DSM-IV | VENLAFAXINE | 33 | 33 | HDRS |  | 27.27 | 51 |
| 1 | Paslakis | | 2014 | NRCT | DSM-IV | VENLAFAXINE | 10 | 10 | HDRS |  | 35 | 47.55 |
| 1 | Paslakis B | | 2014 | NRCT | DSM-IV | MIRTAZAPINE | 10 | 10 | HDRS |  | 35 | 47.55 |
| 1 | Paslakis B | | 2010 | NRCT | DSM-IV | MIRTAZAPINE | 37 | 37 | HDRS |  | 35.14 | 52.4 |
| 1 | Pena | | 2005 | NRCT | DSM-IV | MIRTAZAPINE | 28 | 28 | HDRS |  | 25 | 41.3 |
| 1 | Perahia | | 2008 | RCT | DSM-IV | VENLAFAXINE | 337 | 337 | HDRS |  | 34.7 | 41.6 |
| 1 | Persons | | 2003 | NRCT | DSM | CBT | 58 | 58 | BDI |  | 39.66 | 36.4 |
| 1 | Persons | | 2006 | NRCT | DSM | CBT | 58 | 58 | BDI |  | 39.66 | 36.4 |
| 1 | Perugi | | 2002 | NRCT | DSM-IV | VENLAFAXINE | 21 | 21 | HDRS |  | 21.9 | 30.8 |
| 1 | Peselow | | 2015 | NRCT | DSM-IV | CBT + ESCITALOPRAM | 89 | 89 | MADRS |  | 42 | 32.4 |
| 1 | Petersen | | 2010 | RCT | DSM-III-R | CBT | 11 | 11 | HDRS | BDI | 30 | 42.9 |
| 1 | Pigott | | 2007 | RCT | DSM-IV | ESCITALOPRAM | 274 | 274 | HDRS | MADRS | 32.1 | 43.3 |
| 1 | Pinto | | 2007 | NRCT | DSM-IV | ESCITALOPRAM | 119 | 119 | MADRS |  | 50.4 | 37 |
| 1 | Pjrek | | 2007 | NRCT | DSM-IV-TR | ESCITALOPRAM | 20 | 20 | HDRS |  | 30 | 40.8 |
| 1 | Plesnicar | | 2010 | NRCT | DSM-IV | VENLAFAXINE | 161 | 161 | HDRS |  | 27 | 49.7 |
| 1 | Poirier | | 1999 | RCT | DSM-III-R | VENLAFAXINE | 61 | 61 | HDRS |  | 26.23 | 42.5 |
| 1 | Preeti | | 2018 | NRCT | ICD-10 | ESCITALOPRAM | 47 | 47 | HDRS |  | 69.92 | 36.5 |
| 1 | Preeti | | 2018 | NRCT | ICD-10 | MIRTAZAPINE | 28 | 28 | HDRS |  | 69.92 | 36.5 |
| 1 | Probst | | 2015 | NRCT | DSM-IV | MIRTAZAPINE | 60 | 60 | HDRS |  | 46.67 | 50.7 |
| 1 | Proft | | 2014 | NRCT | ICD-10 | VENLAFAXINE | 56 | 56 | CGI |  | 42.9 | 41.57 |
| 1 | Propst | | 1992 | NRCT | DSM-III | CBT | 19 | 19 | BDI | HDRS | 16.95 | 40 |
| 1 | Quartini | | 2014 | NRCT | DSM-IV-TR | ESCITALOPRAM | 27 | 27 | MADRS | BDI | 0 | N/A |
| 1 | Quera-Salva | | 2011 | RCT | DSM-IV | ESCITALOPRAM | 67 | 44 | HDRS |  | 39 | 41.4 |
| 1 | Quilty | | 2014 | NRCT | DSM-IV | CBT | 53 | 49 | BDI-II | HDRS | 47.12 | 33.61 |
| 1 | Quilty | | 2017 | NRCT | DSM-IV | CBT | 37 | 37 | BDI-II | HDRS | 25.33 | 38.73 |
| 1 | Rapaport | | 2004 | NRCT | DSM-IV | ESCITALOPRAM | 181 | 181 | MADRS | HDRS | 39.8 | 42.9 |
| 1 | Raskin | | 2012 | RCT | DSM-IV | ESCITALOPRAM | 239 | 227 | MADRS |  | 25.1 | N/A |
| 1 | Raue | | 2009 | RCT | DSM-IV | ESCITALOPRAM | N/A | N/A | HDRS |  | 22 | 51.2 |
| 1 | Renaud | | 2013 | NRCT | DSM-IV | CBT | 85 | 69 | BDI-II |  | 37.73 | 38.15 |
| 1 | Renaud | | 2014 | NRCT | DSM-IV-TR | CBT | 256 | 256 | CGI |  | 34.38 | 37.2 |
| 1 | Richards | | 2017 | RCT | DSM | ESCITALOPRAM | 244 | 244 | MADRS |  | 32.1 | 43.7 |
| 1 | Richards | | 2016 | RCT | DSM-IV | CBT | 219 | 180 | PHQ-9 |  | 32 | 43 |
| 1 | Richards B | | 2017 | RCT | DSM | VENLAFAXINE | 144 | 144 | MADRS |  | 32.1 | 43.7 |
| 1 | Ritchey | | 2011 | NRCT | DSM-IV | CBT | 22 | 22 | BDI |  | 40.1 | 36.1 |
| 1 | Rojo | | 2005 | NRCT | DSM-IV | MIRTAZAPINE | 582 | 582 | HDRS |  | 34.7 | 45.4 |
| 1 | Romeo | | 2004 | RCT | DSM-IV | MIRTAZAPINE | 93 | 93 | HDRS |  | N/A | N/A |
| 1 | Romera | | 2012 | NRCT | DSM-IV-TR | ESCITALOPRAM | 840 | 568 | HDRS |  | 30.55 | 47.85 |
| 1 | Rominger | | 2015 | NRCT | DSM-IV | ESCITALOPRAM | 27 | 19 | HDRS | BDI | 37 | 42 |
| 1 | Roose | | 2008 | NRCT | DSM-IV | MIRTAZAPINE | 119 | 119 | HDRS | BDI | 28 | 82.9 |
| 1 | Roose | | 2004 | NRCT | DSM-IV | VENLAFAXINE | 17 | 17 | HDRS |  | 23.5 | 65.6 |
| 1 | Rosenblau | | 2012 | NRCT | DSM-IV | ESCITALOPRAM | 12 | 12 | HDRS | BDI | 58.33 | 43.5 |
| 1 | Rossini | | 2005 | RCT | DSM-IV | ESCITALOPRAM | 17 | 17 | HDRS |  | 23.53 | 42.2 |
| 1 | Rossini B | | 2005 | RCT | DSM-IV | VENLAFAXINE | 16 | 16 | HDRS |  | 12.5 | 49.1 |
| 1 | Rothschild | | 2013 | NRCT | DSM-IV-TR | MIRTAZAPINE | 12 | 12 | MADRS |  | N/A | N/A |
| 1 | Rubin-Falcone | | 2018 | NRCT | DSM-IV | CBT | 33 | 23 | HDRS | BDI | 45 | 34.2 |
| 1 | Rudolph | | 1999 | RCT | DSM-IV | VENLAFAXINE | 100 | 95 | HDRS | MADRS | 27 | 40 |
| 1 | Rudolph | | 1998 | RCT | DSM-III | VENLAFAXINE | 231 | 231 | HDRS | MADRS | 61.33 | 43.5 |
| 1 | Rush | | 2011 | NRCT | DSM-IV-TR | ESCITALOPRAM | 224 | 151 | HDRS |  | 36.2 | 43.6 |
| 1 | Rush | | 2005 | NRCT | DSM-IV | ESCITALOPRAM | 5453 | 5453 | HDRS |  | 28.2 | 44.9 |
| 1 | Rzezniczek | | 2016 | NRCT | DSM-IV | ESCITALOPRAM | N/A | N/A | MADRS | HDRS | 16.7 | 46.7 |
| 1 | Rzezniczek B | | 2016 | NRCT | DSM-IV | MIRTAZAPINE | N/A | N/A | MADRS | HDRS | 16.7 | 46.7 |
| 1 | Rzezniczek C | | 2016 | NRCT | DSM-IV | VENLAFAXINE | N/A | N/A | MADRS | HDRS | 16.7 | 46.7 |
| 1 | Sachsenweger | | 2015 | NRCT | DSM-IV-TR | CBT | 28 | 28 | BDI-II |  | 36 | N/A |
| 1 | Saghafi | | 2007 | NRCT | DSM-IV | ESCITALOPRAM | 175 | 175 | HDRS |  | 59.33 | 73.17 |
| 1 | Saiz-Ruiz | | 2005 | NRCT | DSM-IV | MIRTAZAPINE | 78 | 78 | HDRS |  | 38.46 | 41.12 |
| 1 | Saiz-Ruiz | | 2002 | NRCT | DSM-IV | VENLAFAXINE | 69 | 69 | HDRS | MADRS | 29 | 48.9 |
| 1 | Saloonsson | | 2017 | RCT | ICD-10 | CBT | 80 | 72 | MADRS |  | 28 | 37.2 |
| 1 | Sambataro | | 2018 | NRCT | DSM-IV | CBT | 25 | 25 | BDI-II |  | 56 | 38.4 |
| 1 | Samson | | 2011 | NRCT | DSM-IV | MIRTAZAPINE | 21 | 21 | HDRS |  | 66.66 | 41.52 |
| 1 | Samuelian | | 1998 | RCT | DSM-III-R | VENLAFAXINE | 52 | 52 | HDRS | MADRS | 27 | 47 |
| 1 | Sanacira | | 2006 | NRCT | DSM | CBT | 15 | 8 | HDRS |  | N/A | N/A |
| 1 | Sarubin | | 2014 | NRCT | DSM-IV | ESCITALOPRAM | 28 | 28 | HDRS |  | 60.71 | 39.36 |
| 1 | Sarubin | | 2014 | RCT | DSM-IV | ESCITALOPRAM | 28 | 28 | HDRS |  | 71.7 | 40.25 |
| 1 | Sauer | | 2003 | RCT | ICD-10 | VENLAFAXINE | 79 | 60 | HDRS |  | 32.9 | 48.5 |
| 1 | Sava | | 2009 | RCT | DSM-IV | CBT | 49 | 49 | BDI | HDRS | N/A | N/A |
| 1 | Savaskan | | 2008 | NRCT | ICD-10 | ESCITALOPRAM | 18 | 18 | GDS |  | 22.22 | 76.2 |
| 1 | Schaefer | | 2006 | NRCT | DSM-IV | VENLAFAXINE | 9 | 9 | HDRS | BDI | 33.3 | 36.5 |
| 1 | Scharnholz | | 2010 | NRCT | DSM-IV | MIRTAZAPINE | 42 | 36 | HDRS |  | 35.71 | 49.3 |
| 1 | Scharnholz B | | 2010 | NRCT | DSM-IV | VENLAFAXINE | 45 | 36 | HDRS |  | 26.66 | 52.1 |
| 1 | Schatzberg | | 2006 | RCT | DSM-IV | VENLAFAXINE | 104 | 104 | HDRS | MADRS | 44 | 71 |
| 1 | Schindler | | 2011 | NRCT | DSM-IV | CBT | 338 | 338 | BDI |  | 31.7 | 37.8 |
| 1 | Schindler | | 2013 | NRCT | DSM-IV | CBT | 193 | 164 | BDI |  | 31.6 | 38.6 |
| 1 | Schittecatte | | 2002 | NRCT | DSM-IV | MIRTAZAPINE | 17 | 17 | HDRS |  | 47.18 | 43.6 |
| 1 | Schittecatte B | | 2002 | NRCT | DSM-IV | MIRTAZAPINE | 18 | 18 | HDRS |  | 20 | 39.8 |
| 1 | Schlagert | | 2017 | NRCT | DSM-IV | CBT | 639 | 639 | BDI |  | 33.8 | 38.4 |
| 1 | Schmid | | 2006 | NRCT | ICD-10 | MIRTAZAPINE | 12 | 10 | HDRS | MADRS | 25 | 39.9 |
| 1 | Schmidt | | 2015 | NRCT | DSM-IV/ICD-10 | ESCITALOPRAM | 16 | 16 | HDRS | BDI-II | 40 | 35.5 |
| 1 | Schmidt B | | 2015 | NRCT | DSM-IV/ICD-10 | MIRTAZAPINE | 10 | 10 | HDRS | BDI-II | 40 | 35.5 |
| 1 | Schramm | | 2016 | RCT | DSM | ESCITALOPRAM | 31 | 26 | MADRS |  | 47 | 42.7 |
| 1 | Schule | | 2003 | NRCT | DSM-IV | MIRTAZAPINE | 40 | 40 | HDRS |  | 42.5 | 47.6 |
| 1 | Schule | | 2009 | NRCT | DSM-IV | MIRTAZAPINE | 12 | 12 | HDRS |  | 46.49 | 48.48 |
| 1 | Schule | | 2006 | NRCT | DSM-IV | MIRTAZAPINE | 20 | 20 | HDRS |  | 50 | 48.8 |
| 1 | Schule | | 2003 | NRCT | DSM-IV | MIRTAZAPINE | 20 | 20 | HDRS |  | 70 | 49 |
| 1 | Schule | | 2007 | NRCT | DSM-IV | MIRTAZAPINE | 23 | 23 | HDRS |  | 34.78 | 51.89 |
| 1 | Schule | | 2010 | NRCT | DSM-IV | MIRTAZAPINE | 23 | 23 | HDRS |  | 17.39 | 52.26 |
| 1 | Schule B | | 2007 | NRCT | DSM-IV | MIRTAZAPINE | 20 | 20 | HDRS |  | 20 | 50.3 |
| 1 | Schweitzer | | 2001 | NRCT | DSM-IV | VENLAFAXINE | 274 | 274 | MADRS |  | 39 | 45 |
| 1 | Schweizer | | 1991 | RCT | DSM-III-R | VENLAFAXINE | 60 | 60 | HDRS | MADRS | 63 | 45.7 |
| 1 | Schweizer | | 2010 | NRCT | DSM-IV | CBT | 45 | 45 | HDRS | BDI-II | 43 | 42 |
| 1 | Sefarty | | 2009 | RCT | DSM | CBT | 70 | 59 | BDI-II |  | 15.7 | 74.4 |
| 1 | Seo | | 2009 | NRCT | DSM-IV | MIRTAZAPINE | 47 | 47 | CGI |  | 24.4 | 51 |
| 1 | Seo B | | 2009 | NRCT | DSM-IV | VENLAFAXINE | 12 | 12 | BDI |  | 24.4 | 51 |
| 1 | Seripa | | 2016 | NRCT | DSM-IV-TR | ESCITALOPRAM | 100 | 100 | HDRS |  | 28.21 | 76.38 |
| 1 | Shafran | | 2018 | NRCT | DSM-IV | CBT | 69 | 69 | PHQ-9 |  | 34.78 | 44.61 |
| 1 | Shams | | 2006 | NRCT | DSM-IV | VENLAFAXINE | 25 | 25 | CGI |  | 54 | 52 |
| 1 | Shamsaei | | 2008 | RCT | DSM-IV | CBT | 40 | 40 | BDI |  | 10 | 36 |
| 1 | Shaprio | | 1990 | NRCT | DSM-III | CBT | 24 | 24 | BDI |  | N/A | N/A |
| 1 | Sheehan | | 2009 | RCT | DSM-IV | VENLAFAXINE | 95 | 95 | HDRS | MADRS | 52 | 41.7 |
| 1 | Shelton | | 2006 | RCT | DSM-IV | VENLAFAXINE | 78 | 78 | HDRS |  | 39 | 37.2 |
| 1 | Shen | | 2011 | NRCT | DSM-IV | MIRTAZAPINE | 42 | 42 | BDI-II | HDRS | 19.05 | 43.9 |
| 1 | Shen | | 2009 | RCT | DSM-IV | MIRTAZAPINE | 28 | 28 | BDI-II |  | 14.29 | 45.9 |
| 1 | Shen | | 2006 | NRCT | DSM-IV | MIRTAZAPINE | 16 | 16 | HDRS | BDI-II | 12.5 | 47.1 |
| 1 | Shou | | 2017 | NRCT | DSM-IV-TR | CBT | 17 | 17 | MADRS |  | N/A | 31.88 |
| 1 | Shrivasta | | 1994 | RCT | DSM-III-R | VENLAFAXINE | 290 | 290 | CGI |  | 45.52 | 42 |
| 1 | Si | | 2017 | NRCT | DSM-IV | ESCITALOPRAM | 225 | 168 | MADRS | HDRS | 40.6 | 40.7 |
| 1 | Sicras | | 2010 | NRCT | ICD-9 | ESCITALOPRAM | 131 | 131 | HDRS |  | 32.1 | 49.7 |
| 1 | Sicras B | | 2010 | NRCT | ICD-9 | VENLAFAXINE | 343 | 343 | HDRS |  | 23.7 | 56.5 |
| 1 | Sidi | | 2012 | NRCT | DSM-IV | ESCITALOPRAM | 56 | 56 | MADRS | HDRS | 0 | 40.82 |
| 1 | Silverston | | 1999 | RCT | DSM-IV | VENLAFAXINE | 128 | 122 | HDRS |  | 36.07 | 41.1 |
| 1 | Simon | | 2004 | NRCT | DSM-IV | VENLAFAXINE | 490 | 490 | HDRS |  | 37 | 42 |
| 1 | Singh | | 2012 | NRCT | DSM-IV | ESCITALOPRAM | 57 | 57 | HDRS |  | 23 | 38 |
| 1 | Singh B | | 2012 | NRCT | DSM-IV | VENLAFAXINE | 41 | 41 | HDRS |  | 15 | 41 |
| 1 | Sinha | | 2017 | NRCT | ICD-10 | MIRTAZAPINE | 28 | 28 | HDRS |  | 41.1 | 36.5 |
| 1 | Sinha B | | 2017 | NRCT | ICD-10 | ESCITALOPRAM | 47 | 47 | HDRS |  | 41.1 | 36.5 |
| 1 | Sinniah | | 2016 | RCT | DSM-IV | CBT | 33 | 28 | BDI | HDRS | 30.43 | 43.13 |
| 1 | Sir | | 2005 | RCT | DSM-IV | VENLAFAXINE | 84 | 84 | HDRS |  | 33.3 | 36.8 |
| 1 | Sirot | | 2012 | NRCT | DSM-IV | MIRTAZAPINE | 44 | 44 | HDRS |  | 28.89 | 49.25 |
| 1 | Skibinska | | 2018 | NRCT | DSM-IV | VENLAFAXINE | 30 | 30 | HDRS |  | 0 | 38.06 |
| 1 | Smagula | | 2016 | NRCT | DSM-IV | VENLAFAXINE | 181 | 181 | HDRS | MADRS | N/A | N/A |
| 1 | Smith | | 2009 | NRCT | DSM-IV | MIRTAZAPINE | 15 | 15 | HDRS |  | 26.67 | 43.3 |
| 1 | Soares | | 2011 | NRCT | DSM-IV | ESCITALOPRAM | 60 | 60 | HDRS | MADRS | 0 | 55 |
| 1 | Soares | | 2010 | RCT | DSM-IV | ESCITALOPRAM | 299 | 237 | HDRS | MADRS | 0 | 56 |
| 1 | Soares | | 2006 | NRCT | DSM-IV | ESCITALOPRAM | 16 | 16 | MADRS |  | 0 | 50 |
| 1 | Soczynska | | 2014 | RCT | DSM | ESCITALOPRAM | 19 | 19 | HDRS |  | 47.4 | 41.3 |
| 1 | Song | | 2015 | NRCT | DSM-IV-TR | MIRTAZAPINE | 93 | 93 | HDRS | MADRS | 24.7 | 50.87 |
| 1 | Sorensen | | 2007 | NRCT | DSM-IV | ESCITALOPRAM | N/A | N/A | MADRS |  | N/A | N/A |
| 1 | Sorensen | | 2007 | NRCT | DSM-IV | VENLAFAXINE | N/A | N/A | MADRS |  | N/A | N/A |
| 1 | Souery | | 2015 | NRCT | DSM-IV-TR | VENLAFAXINE | 417 | 417 | MADRS | HDRS | 33.57 | 47.29 |
| 1 | Spies | | 2017 | NRCT | DSM-IV | ESCITALOPRAM | 23 | 23 | HDRS |  | 30.43 | N/A |
| 1 | Staeker | | 2014 | NRCT | ICD-10 | VENLAFAXINE | 32 | 32 | CGI |  | 33.51 | N/A |
| 1 | Staeker B | | 2014 | NRCT | ICD-10 | ESCITALOPRAM | 36 | 36 | CGI |  | 33.51 | N/A |
| 1 | Staeker C | | 2014 | NRCT | ICD-10 | MIRTAZAPINE | 82 | 82 | CGI |  | 42.68 | N/A |
| 1 | Stamm | | 2014 | NRCT | ICD-10 | VENLAFAXINE | 165 | 88 | HDRS |  | 29.55 | 45.39 |
| 1 | Stange | | 2017 | NRCT | DSM-IV | CBT | 32 | 32 | HDRS |  | 15.6 | 24.03 |
| 1 | Stewart | | 2015 | NRCT | DSM-IV-TR | ESCITALOPRAM | 84 | 69 | HDRS | MADRS | 33 | 41 |
| 1 | Stile-Sheilds | | 2015 | RCT | DSM | CBT | 162 | 162 | HDRS |  | 21.6 | 47.5 |
| 1 | Stoy | | 2012 | NRCT | DSM-IV | ESCITALOPRAM | 15 | 15 | HDRS | BDI | 66.67 | 41.9 |
| 1 | Stubbings | | 2013 | RCT | DSM-IV-TR | CBT | 12 | 10 | DASS |  | 41.66 | 30 |
| 1 | Su | | 2016 | NRCT | DSM-IV-TR | ESCITALOPRAM | 166 | 166 | MADRS | HDRS | 37 | 41 |
| 1 | Tadic | | 2010 | RCT | DSM-IV | CBT | 56 | 56 | HDRS |  | 23.2 | 51.3 |
| 1 | Tadic | | 2016 | RCT | DSM-IV | ESCITALOPRAM | 879 | 879 | HDRS |  | N/A | N/A |
| 1 | Tadic | | 2007 | RCT | DSM-IV | MIRTAZAPINE | 54 | 54 | HDRS | MADRS | 26.47 | 48.3 |
| 1 | Takamura | | 2017 | NRCT | DSM-IV | ESCITALOPRAM | 12 | 12 | HDRS | BDI-II | 50 | 38.3 |
| 1 | Taranu | | 2017 | NRCT | DSM-IV-TR | VENLAFAXINE | 206 | 206 | HDRS |  | 33.98 | 48.33 |
| 1 | Taranu | | 2017 | NRCT | DSM-IV-TR | VENLAFAXINE | 206 | 180 | HDRS |  | 33.78 | 48.54 |
| 1 | Taylor | | 2010 | NRCT | DSM-IV | CBT | 84 | 84 | HDRS |  | 27 | 42.7 |
| 1 | Teismann | | 2012 | RCT | DSM-IV | CBT | 70 | 70 | BDI |  | 34.33 | 45.9 |
| 1 | Terhardt | | 2013 | NRCT | DSM-IV | VENLAFAXINE | 20 | 20 | HDRS |  | 45 | 47.1 |
| 1 | Terhardt B | | 2013 | NRCT | DSM-IV | MIRTAZAPINE | 24 | 24 | HDRS |  | 45.83 | 47.1 |
| 1 | Thase | | 1992 | NRCT | DSM-III-R | CBT | 38 | 38 | HDRS | BDI | 27 | 37.5 |
| 1 | Thase | | 1994 | NRCT | DSM-III-R | CBT | 45 | 45 | HDRS | BDI | 100 | 38.4 |
| 1 | Thase | | 2000 | NRCT | DSM-III-R | CBT | 52 | 52 | HDRS | BDI | 100 | 38.4 |
| 1 | Thase | | 2018 | NRCT | DSM-IV | CBT | 77 | 77 | HDRS | BDI-II | 32.5 | 46 |
| 1 | Thase | | 1996 | NRCT | DSM-III-R | CBT | 29 | 29 | HDRS |  | 55.17 | 32.7 |
| 1 | Thase | | 1991 | NRCT | DSM-III-R | CBT | 16 | 16 | HDRS |  | N/A | 35.2 |
| 1 | Thase | | 1998 | NRCT | DSM-III-R | CBT | 90 | 90 | HDRS |  | 44 | 38.4 |
| 1 | Thase | | 1991 | NRCT | DSM-III-R | CBT | 59 | 59 | HDRS |  | 31 | 43.35 |
| 1 | Thase | | 2001 | NRCT | DSM-IV | MIRTAZAPINE | 410 | 410 | HDRS |  | 43.9 | 39.5 |
| 1 | Thase | | 1997 | RCT | DSM-IV | VENLAFAXINE | 95 | 91 | HDRS | MADRS | 36.84 | 40 |
| 1 | Thase | | 2006 | RCT | DSM-IV-TR | VENLAFAXINE | 174 | 164 | HDRS |  | 36 | 37.4 |
| 1 | Thase B | | 1994 | NRCT | DSM-III-R | CBT | 84 | 84 | BDI |  | 100 | 37.9 |
| 1 | Thase B | | 1992 | NRCT | DSM-III-R | CBT | 50 | 50 | HDRS | BDI | 30 | 37.3 |
| 1 | Thase B | | 1991 | NRCT | DSM-III-R | CBT | 38 | 38 | HDRS | BDI | 21 | 39.8 |
| 1 | Thase B | | 2006 | RCT | DSM-IV | VENLAFAXINE | 232 | 232 | HDRS |  | 31.58 | 38.5 |
| 1 | Tian | | 2016 | NRCT | DSM-IV | VENLAFAXINE | 34 | 34 | HDRS |  | 29.41 | 36.1 |
| 1 | Titov | | 2015 | RCT | DSM-IV | CBT | 141 | 141 | PHQ-9 |  | 29 | 44.55 |
| 1 | Trick | | 2004 | RCT | DSM-IV | VENLAFAXINE | 45 | 45 | MADRS | HDRS | 31.11 | 71.5 |
| 1 | Trivedi | | 2013 | RCT | DSM-IV-TR | ESCITALOPRAM | 246 | 239 | HDRS | MADRS | 40.6 | 37.9 |
| 1 | Tsai | | 2010 | NRCT | DSM-IV-TR | ESCITALOPRAM | 100 | 100 | HDRS |  | 18 | 42 |
| 1 | Tsutsumi | | 2016 | NRCT | DSM-IV | MIRTAZAPINE | 72 | 68 | CGI |  | 44.12 | 49.35 |
| 1 | Tzanakaki | | 2000 | RCT | DSM-IV | VENLAFAXINE | 55 | 55 | HDRS | MADRS | 25.45 | 47 |
| 1 | Tzeng | | 2009 | NRCT | DSM-IV | MIRTAZAPINE | 58 | 58 | HDRS |  | 44.83 | 47.3 |
| 1 | Udristoiu | | 2016 | RCT | DSM-IV-TR | ESCITALOPRAM | 143 | 118 | HDRS |  | 19.6 | 46.4 |
| 1 | Ueno | | 2015 | RCT | DSM-IV | MIRTAZAPINE | 194 | 194 | HDRS |  | 50.8 | 39.1 |
| 1 | Vai | | 2016 | NRCT | DSM-IV | ESCITALOPRAM | 33 | 33 | HDRS |  | 42.42 | 30.34 |
| 1 | Vanderkooy | | 2002 | NRCT | DSM-IV | VENLAFAXINE | 62 | 62 | HDRS |  | 38.71 | 40.8 |
| 1 | VanMoffaert | | 1995 | RCT | DSM-III | MIRTAZAPINE | 100 | 100 | MADRS |  | 31 | 46.1 |
| 1 | Vartianinen | | 1994 | RCT | DSM-III | MIRTAZAPINE | 59 | 59 | HDRS | MADRS | 42.37 | 45.8 |
| 1 | Ventriglia | | 2009 | NRCT | ICD-10 | ESCITALOPRAM | 25 | 25 | HDRS |  | 20 | 43.36 |
| 1 | Ventura | | 2007 | RCT | DSM-IV | ESCITALOPRAM | 104 | 104 | HDRS | MADRS | 45.2 | 40.6 |
| 1 | Vermeiden | | 2013 | RCT | DSM-IV | VENLAFAXINE | 44 | 42 | HDRS |  | 50 | 53 |
| 1 | Versiani | | 2005 | RCT | DSM-IV | MIRTAZAPINE | 145 | 145 | HDRS | MADRS | 26 | 43 |
| 1 | Victoria | | 2019 | NRCT | DSM | ESCITALOPRAM | 20 | 20 | MADRS | HDRS | 40 | 70.4 |
| 1 | Vieta | | 2018 | RCT | DSM-IV-TR | ESCITALOPRAM | 49 | 49 | MADRS |  | 28.6 | 49.7 |
| 1 | Wade | | 2002 | RCT | DSM-IV | ESCITALOPRAM | 191 | 191 | MADRS |  | 26.2 | 41 |
| 1 | Wade | | 2006 | RCT | DSM-IV | ESCITALOPRAM | 590 | 590 | MADRS |  | 25 | 42 |
| 1 | Wade | | 2007 | RCT | DSM-IV-TR | ESCITALOPRAM | 144 | 141 | MADRS |  | 25.9 | 43.3 |
| 1 | Wade | | 2011 | NRCT | DSM-IV | ESCITALOPRAM | 60 | 60 | MADRS |  | 13.3 | 43.5 |
| 1 | Wade | | 2003 | RCT | DSM-IV | MIRTAZAPINE | 93 | 93 | HDRS |  | 25 | 40 |
| 1 | Walinder | | 2006 | NRCT | DSM-IV | MIRTAZAPINE | 192 | 192 | MADRS |  | 34 | 49.8 |
| 1 | Wang | | 2017 | NRCT | DSM-IV | ESCITALOPRAM | 20 | 20 | HDRS |  | 45 | 34.6 |
| 1 | Wang | | 2018 | NRCT | DSM-IV | ESCITALOPRAM | 142 | 85 | HDRS |  | 34.1 | 36.7 |
| 1 | Wang | | 2014 | RCT | DSM-IV | ESCITALOPRAM | 157 | 69 | MADRS | HDRS | 24.3 | 40.3 |
| 1 | Wang | | 2011 | NRCT | DSM-IV | VENLAFAXINE | 115 | 115 | HDRS |  | 26 | 36.2 |
| 1 | Wang | | 2015 | RCT | DSM-IV-TR | VENLAFAXINE | 230 | 215 | MADRS |  | 38.5 | 41 |
| 1 | Wang B | | 2014 | NRCT | DSM-IV | ESCITALOPRAM | 14 | 14 | HDRS |  | 35.71 | 32.93 |
| 1 | Ward | | 2000 | RCT | ICD-10 | CBT | 63 | 63 | BDI |  | 25 | 37 |
| 1 | Watson | | 2003 | NRCT | DSM-IV | CBT | 66 | 45 | BDI |  | 33 | 41.52 |
| 1 | Watson | | 2008 | NRCT | DSM-IV | CBT | 90 | 90 | BDI-II |  | 38.89 | 34.97 |
| 1 | Weissman | | 2015 | RCT | DSM-IV | ESCITALOPRAM | 76 | 76 | HDRS | MADRS | 0 | 38.2 |
| 1 | Wheatley | | 1998 | NRCT | DSM-III-R | MIRTAZAPINE | 60 | 60 | HDRS |  | 45 | 47.2 |
| 1 | Whyte | | 2006 | NRCT | DSM-IV | VENLAFAXINE | 46 | 46 | HDRS |  | 32.3 | 74 |
| 1 | Wijkstra | | 2010 | RCT | DSM-IV-TR | VENLAFAXINE | 39 | 39 | HDRS |  | 56.4 | 49.5 |
| 1 | Wingman | | 2014 | NRCT | DSM-IV | CBT | N/A | N/A | BDI |  | 40 | 43.5 |
| 1 | Winkler | | 2007 | NRCT | ICD-10 | ESCITALOPRAM | 617 | 617 | CGI |  | 39.5 | 43.4 |
| 1 | Winkour | | 2003 | NRCT | DSM-IV | MIRTAZAPINE | 19 | 19 | HDRS |  | 66.7 | 40.9 |
| 1 | Wolkowitz | | 2011 | NRCT | DSM-IV | ESCITALOPRAM | 15 | 15 | HDRS |  | 100 | 41.4 |
| 1 | Wollburg | | 2010 | NRCT | ICD-10 | CBT | 657 | 657 | BDI |  | 31.5 | 45.2 |
| 1 | Won | | 2012 | NRCT | DSM-IV | ESCITALOPRAM | 115 | 115 | HDRS |  | 10 | 46.64 |
| 1 | Woo | | 2017 | RCT | DSM-IV | VENLAFAXINE | 151 | 63 | HDRS | MADRS | 28.5 | N/A |
| 1 | Woo B | | 2017 | RCT | DSM-IV | ESCITALOPRAM | 148 | 51 | HDRS | MADRS | 18.2 | N/A |
| 1 | Wroolie | | 2006 | NRCT | DSM-IV | ESCITALOPRAM | 17 | 15 | HDRS |  | 0 | 55.94 |
| 1 | Wu | | 2007 | NRCT | DSM-IV | VENLAFAXINE | 78 | 49 | HDRS |  | 34.6 | 39.72 |
| 1 | Yamada | | 2018 | NRCT | DSM-IV-TR | CBT | 19 | 19 | BDI-II |  | 42.11 | 38.4 |
| 1 | Yastrebov | | 2012 | NRCT | ICD-10 | VENLAFAXINE | 30 | 30 | HDRS |  | N/A | 37.6 |
| 1 | Yazicioglu | | 2006 | RCT | DSM-IV | VENLAFAXINE | 20 | 20 | HDRS |  | 15 | 42.2 |
| 1 | Yeh | | 2015 | NRCT | DSM-IV-TR | VENLAFAXINE | 294 | 161 | HDRS |  | 42.7 | 37.4 |
| 1 | Yevtushenko | | 2007 | RCT | DSM-IV | ESCITALOPRAM | 108 | 108 | MADRS |  | 38.9 | 35.19 |
| 1 | Yoo | | 2015 | RCT | DSM-IV | ESCITALOPRAM | 82 | 82 | HDRS |  | 18.29 | 57.77 |
| 1 | Yoon | | 2016 | RCT | DSM-IV | ESCITALOPRAM | 17 | 17 | HDRS |  | 0 | 45.6 |
| 1 | Yoshimura | | 2014 | NRCT | DSM-IV | CBT | 23 | 23 | BDI | HDRS | 69.57 | 37.3 |
| 1 | Yoshimura | | 2017 | NRCT | DSM-IV-TR | CBT | 29 | 29 | HDRS | BDI | 65.52 | 37.4 |
| 1 | Yu | | 2013 | RCT | DSM-IV | ESCITALOPRAM | 130 | 130 | HDRS |  | 35.38 | 37.2 |
| 1 | Yuen | | 2015 | NRCT | DSM-IV-TR | ESCITALOPRAM | 71 | 71 | HDRS |  | 45.07 | 70.8 |
| 1 | Zdanowicz | | 2017 | NRCT | DSM-IV-TR | ESCITALOPRAM | 9 | 9 | HDRS |  | 17.5 | 40.33 |
| 1 | Zhou | | 2015 | NRCT | DSM-IV | ESCITALOPRAM | 25 | 25 | HDRS |  | 48 | 45 |
| 1 | Zimmer | | 1997 | NRCT | DSM-IV | VENLAFAXINE | 34 | 34 | CGI |  | 52.94 | 55.38 |
| 1 | Zimmermann | | 2015 | NRCT | DSM-IV | CBT | 24 | 24 | BDI |  | 12.5 | 34.3 |
| 1 | Zu | | 2014 | NRCT | ICD-10 | CBT | 30 | 12 | HDRS |  | 50 | 32.7 |
| 2 | Brunoni | | 2020 | RCT | DSM-V | ESCITALOPRAM | 75 | 75 | HDRS |  | 29.7 | 41.4 |
| 2 | Carboni | | 2019 | RCT | DSM-IV-TR | VENLAFAXINE | 51 | 51 | HDRS | MADRS | 43 | 44.8 |
| 2 | Cook | | 2020 | RCT | DSM-IV | ESCITALOPRAM | 75 | 75 | HDRS |  | 40 | 37.2 |
| 2 | Davey | | 2019 | RCT | DSM-IV | CBT | 77 | 77 | MADRS |  | 42 | 19.4 |
| 2 | DeRubeis | | 2020 | RCT | DSM-IV | VENLAFAXINE | N/A | N/A | HDRS |  | 41 | 45.1 |
| 2 | DosSantos | | 2020 | RCT | DSM | CBT | 113 | 58 | BDI-II |  | 20 | 32 |
| 2 | Dreimuller | | 2019 | NRCT | DSM-IV | ESCITALOPRAM | 879 | 879 | HDRS |  | 56 | 39.15 |
| 2 | Fountoulakis | | 2019 | NRCT | DSM-IV-TR | ESCITALOPRAM | 29 | 8 | CESD |  | 31.67 | 39.72 |
| 2 | Gorka | | 2019 | RCT | DSM-V | CBT | 54 | 27 | HDRS |  | 29.63 | 29.74 |
| 2 | Jeng | | 2020 | NRCT | DSM-IV | ESCITALOPRAM | 36 | 36 | HDRS |  | 47.22 | 44.94 |
| 2 | Kraus | | 2019 | NRCT | DSM-IV | ESCITALOPRAM | N/A | N/A | HDRS | BDI | N/A | N/A |
| 2 | Langenecker | | 2019 | NRCT | DSM-IV-TR | ESCITALOPRAM | 22 | 22 | HDRS |  | 38.89 | 35.89 |
| 2 | Leuzinger | | 2019 | RCT | DSM-IV | CBT | 41 | 41 | BDI |  | 32.5 | 40.62 |
| 2 | Levada | | 2019 | NRCT | DSM-V | ESCITALOPRAM | 25 | 20 | MADRS |  | 40 | 37.2 |
| 2 | Lin | | 2019 | NRCT | DSM-IV | VENLAFAXINE | 11 | 11 | MADRS |  | N/A | N/A |
| 2 | Meyer | | 2019 | NRCT | DSM-IV | ESCITALOPRAM | 22 | 22 | MADRS | HDRS | 50 | 31.5 |
| 2 | Mishra | | 2019 | NRCT | DSM-V | VENLAFAXINE | 35 | 35 | MADRS |  | 34.29 | 36.1 |
| 2 | Navarro | | 2019 | NRCT | DSM-IV | VENLAFAXINE | 112 | 112 | HDRS |  | 33.01 | 48.29 |
| 2 | Pantazatos | | 2020 | NRCT | DSM-IV | CBT | N/A | N/A | HDRS | BDI | N/A | N/A |
| 2 | Shen | | 2019 | RCT | DSM-IV | ESCITALOPRAM | 268 | 268 | HDRS | MADRS | 35.8 | 37.6 |
| 2 | Sun | | 2019 | NRCT | DSM-V | VENLAFAXINE | 193 | 193 | HDRS |  | 52 | 36.55 |
| 2 | Sun | | 2020 | NRCT | DSM-V | ESCITALOPRAM | 78 | 78 | HDRS |  | 35.9 | 40.21 |
| 2 | Tian | | 2020 | NRCT | DSM | ESCITALOPRAM | 106 | 106 | HDRS |  | 51.89 | 32.87 |
| 2 | van der Schans | | 2019 | RCT | DSM-IV | VENLAFAXINE | 39 | 39 | MADRS |  | N/A | N/A |
| 2 | von Brachel | | 2019 | NRCT | DSM-IV | CBT | 263 | 263 | BDI |  | N/A | 52 |
| 2 | Xu | | 2019 | NRCT | DSM-IV | ESCITALOPRAM | 306 | 306 | HDRS |  | 54.25 | 39.4 |
| 2 | Yuan | | 2020 | NRCT | DSM-IV | VENLAFAXINE | 195 | 195 | HDRS |  | 24.85 | 36.73 |
| 2 | Zhang | | 2020 | NRCT | DSM-IV | VENLAFAXINE | 175 | 175 | HDRS |  | 45.6 | 37.04 |

Supplementary Materials 21. The Evidence Project Risk of Bias Assessment Tool (Kennedy et al., 2019).

| Study | Cohort | Control/ Comparison | Pre/post Data | Random assignment | Follow-up Rate ≥ 80% | Equivalent across sociodemographics | Equivalent on outcome measures (baseline) | RCT | Funder |
| --- | --- | --- | --- | --- | --- | --- | --- | --- | --- |
| Altenstien-Yamanaka et al., 2017 | Yes | Yes | Yes | Yes | Yes | Yes | Yes | Yes | NR |
| Azvedo da Silva et al., 2017 | Yes | Yes | Yes | Yes | No | Yes | Yes | Yes | NR |
| Basu et al., 2017 | Yes | No | No | NA | No | NA | NA | No | NR |
| Bernecker et al., 2016 | Yes | Yes | Yes | Yes | No | No- CBT higher age | No- CBT group higher clinician-rated depression but self-reported depression comparable | Yes | Ontario Mental Health Foundation |
| Carter et al., 2013 | Yes | Yes | Yes | Yes | No | No- CBT had less comorbid diagnoses than comparator | Yes | Yes | Health Research Council of New Zealand |
| Ciusani et al., 2014 | Yes | No | Yes | NA | Yes | NA | NA | No | Novartis and Wyeth- provided medication |
| Forman et al., 2007 | Yes | Yes | Yes | Yes | No | No- CBT had less males compared to comparator | Yes | Yes | NR |
| Groves et al., 2015 | Yes | Yes | Yes | Yes | No | Yes | Yes | Yes | New Zealand Lottery Board Health Fund, University of Otage Research Fund |
| Halaris et al., 2015 | Yes | Yes | Yes | No | No | Yes | No- matched healthy controls | No | Loyola University Stritch School of Medicine |
| Heller et al., 2013 | Yes | Yes | Yes | Yes | No | NR | NR | Yes | NIMH, Wyeth-Ayerst, Fetzer Institute, John Templeton Foundation, John W. Kluge Foundation, Impact Foundation |
| Huang et al., 2016 | Yes | Yes | No | No | NR | Yes | Yes | No | Ministry of Education, Aim for the Top University Plan, National Science Council (Taiwan), National Taiwan University Hospital, Yun Lin Branch. |
| Lenze et al., 2015 (IRL-GREY) | Yes | Yes | No | Yes | Yes | NR | Yes | Yes | NIMH, UPMC, Taylor Family Institute for Innovative Psychiatric Research, National Center for Advancing Translational Sciences , Campbell Famliy Mental Health Research Institute. |
| Lopes et al., 2014 | Yes | Yes | Yes | Yes | No | Yes | Yes | Yes | Portuguese Foundation for Science and Technology |
| Luty et al., 2007 | Yes | Yes | Yes | Yes | Yes | Yes | Yes | Yes | Health Research Council of New Zealand |
| Myung et al., 2012 | Yes | Yes | No | No | No | NR | NR | No | Korea Science and Engineering Foundation, Ministry of Health and Welfare, Centre fro Genome Research of Samsung Biomedical Research, Lundbeck |
| Nakagawa et al., 2017 | Yes | Yes | Yes | Yes | Yes | NR | Yes | Yes | Japanese Ministry of Health, Labour, and Welfare |
| Saghafi et al., 2017 | Yes | No | No | No | NR | NA | NA | No | P30 MH071944; R01 MH37869, R01 MH43832, EXPORT P60 MD-000-207, T32 MH19986, and the University of Pittsburgh Medical Center (UPMC) endowment in geriatric psychiatry |
| Sefarty et al., 2017 | Yes | Yes | Yes | Yes | Yes | Yes | Yes | Yes | The Health Foundation; and the North Central Thames Research Network |
| Sirot et al., 2012 | Yes | No | Yes | NA | No | NA | NA | No | Swiss National Research Foundation |
| Soczynska et al., 2014 | Yes | Yes | Yes | Yes | Yes | Yes | Yes | Yes | Ontario Government, Ontario Brain Institute |
| Osvath et l., 2007 | Yes | No | No | NA | Yes | NA | NA | No | NR |
| Eddington et al., 2013 | Yes | Yes | No | Yes | No | Yes | Yes | Yes | NIMH |

*Note:* NA = Not Applicable, NR = Not Reported.

Supplementary Materials 22. Results of the goldbricker algorithm analyses.

We have now run the goldbricker algorithm on all networks using the following settings, *goldbricker(data, p=0.05, method= “hittner2003”, threshold=0.25, corMin= 0.5).* The results are as follows:

Less than 25% of correlations are significantly different between the following pairs:

- BDI-II (CBT): *self dislike –sad*, *self dislike-pessimism, concentration-pleasure loss, self critical – self dislike.*
- HDRS: none for CBT or ADM’s.
- MADRS: ADM’s = *lassitude-tension;* CBT = *suicide-reported sad, pessimism-tension, lassitude-concentration*.

Supplementary Materials 23. R.Code for all reported analyses.

CompleteReviewScript.R

Aoife.Whiston

2021-04-28

#### Prep ####

#clean environment

rm(list=ls())

#set working directory

setwd("~/Desktop")
setwd("C:/Users/aoife.whiston/Desktop")
#load packages

library("psych")
library ("bnlearn")
library("qgraph")
library("bootnet")
library("ggplot2")
library("NetworkToolbox")
library("igraph")
library("NetworkComparisonTest")
library("IsingFit")
library("lavaan")
library("caTools")
library("dplyr")
library("rsample")
library("semTools")

##### HDRS-17 data ####


#load CBT AND ADM datasets from csv file


CBTHdat<- read.csv("Complete/CBTHDRS-17.csv", header= TRUE, sep=",", as.is=TRUE)
View(CBTHdat)

ADMHdat<- read.csv("Complete/ADMSHDRS-17.csv", header= TRUE, sep=",", as.is=TRUE)
View(ADMHdat)


#estimate networkS

CBTHnet<- estimateNetwork(CBTHdat,default= "EBICglasso", tuning= 0.0, corMethod = "cor", corArgs = list(method = "spearman"))
pdf(file= "CBTHnetwork.pdf", width=8, height = 11)
CBTHgraph<-plot(CBTHnet, layout="spring", vsize=10 )
dev.off()


ADMHnet<- estimateNetwork(ADMHdat, default= "EBICglasso", tuning= 0.0, corMethod = "cor", corArgs = list(method = "spearman"))
pdf(file= "ADMHnetwork.pdf", width=8, height = 11)
ADMHgraph<-plot(ADMHnet, layout="spring", vsize=10)
dev.off()

l<-averageLayout(CBTHnet, ADMHnet)
layout(t(1:2))
pdf(file= "CBTvADMhdrs.pdf", width=8, height = 11)
plot(CBTHnet, layout=l, vsize=8, title= "CBT")
plot(ADMHnet, layout=l, vsize=8, title= "ADM")
dev.off()


#Centrality Indices

#centrality plots

pdf(file= "CBTHcentrality.pdf", width=8, height = 11)
centralityPlot(CBTHnet, include = c("ExpectedInfluence", "Strength", "Betweenness", "Closeness"))
dev.off()

pdf(file= "ADMHcentrality.pdf", width=8, height = 11)
centralityPlot(ADMHnet, include = c("ExpectedInfluence", "Strength", "Betweenness", "Closeness"))
dev.off()

#centrality table

centralityTable(CBTHnet)

centralityTable(ADMHnet)

#Stability


#edge weight accuracy
#use nBoots to override the default of 1,000, tutorial paper uses 2,500.. 1,000 is often sufficient.

#CBT
CBTHboot1<- bootnet(CBTHnet, nCores = 5)
print(CBTHboot1)
plot(CBTHboot1)
summary(CBTHboot1)

pdf(file= "CBTHboot1.pdf", width=8, height = 11)
plot(CBTHboot1, labels= FALSE, order="sample")
dev.off()

#ADM
ADMHboot1<- bootnet(ADMHnet, nCores = 5)
print(ADMHboot1)
summary(ADMHboot1)

pdf(file= "ADMHboot1.pdf", width=8, height = 11)
plot(ADMHboot1, labels= FALSE, order="sample")
dev.off()


#central stability.using case dropping bootstrap
#investigating the stability of centrality indices by estimating network models based on subsets of the data.

#CBT
CBTHboot2<- bootnet(CBTHnet, type="case", nCores=5)
print(CBTHboot2)
pdf(file= "CBTHboot2.pdf", width=8, height = 11)
plot(CBTHboot2)
dev.off()

#ADM
ADMHboot2<- bootnet(ADMHnet, type="case", nCores=2)
pdf(file= "ADMHboot2.pdf", width=8, height = 11)
plot(ADMHboot2)
dev.off()


#central stability can be quantified using the CS-coefficient, which quantifies the maximum proportions of cases that can be dropped to reatin, with
#95% certainty, a correlation with the originally centrality of higher than (0.7) by default. A cuttoff of 0.5 required to consider the metric stable.

#CBT
corStability(CBTHboot2, cor=0.7, statistics = "all")

#ADM
corStability(ADMHboot2, cor=0.7, statistics = "all")


#plot the difference tests of node strength between all pairs of edge-weightsplot(boot1, "edge", plot="difference"onlyNonZero= TRUE, order="sample")

#CBT
pdf(file= "CBTHboot1b.pdf", width=11, height = 11)
plot(CBTHboot1, "edge", plot= "difference", onlyNonZero = TRUE, order = "sample")
dev.off()

#ADM
pdf(file= "ADMHboot1b.pdf", width=11, height = 11)
plot(ADMHboot1, "edge", plot= "difference", onlyNonZero = TRUE, order = "sample")
dev.off()


#comparing node strength
#bootstrapped difference test (a=.05) of node strength of the symptoms. Gray boxes indicate nodes or edges that do not signifcanlty differ, and
#black boxes represent nodes or edges that do differ signifcantly from one another. White boxes show the value of node strength.

#CBT
pdf(file= "CBTHboot1c.pdf", width=11, height = 11)
plot(CBTHboot1, "strength")
dev.off()


#ADM
pdf(file= "ADMHboot1c.pdf", width=11, height = 11)
plot(ADMHboot1, "strength")
dev.off()

#Network Comparison Test for HDRS
#compare networks and store in object called res (results) and print these results.

set.seed(123)

res<-NCT(CBTHnet, ADMHnet, gamma=0, it=5000, binary.data = FALSE, paired = FALSE, weighted = TRUE, test.edges=TRUE, edges="all", test.centrality= TRUE, progressbar = TRUE )
print(res)
capture.output(res, file="CBTvADMhdrs.txt", append = TRUE)


# plot the results of the invariance of network structure as a whole.
#when this is found to be non-significant do not do further testing on specific edges as this inflates multiple testing and type 1 error.
plot(res, what="network")

#plot results of the global stregnth invariance test.
#hypothesis states the overall connectivity is the same accross networks
plot(res, what="strength")


#### BDI-II data ####

#load CBT AND ADM datasets from csv file

CBTBdat<- read.csv("Complete/CBTBDI-II.csv", header= TRUE, sep=",", as.is=TRUE)

View(CBTBdat)

#estimate networkS EBIC glassoGGM

CBTBnet<- estimateNetwork(CBTBdat, default= "EBICglasso", tuning= 0.0, corMethod = "cor", corArgs = list(method = "spearman"))
pdf(file= "CBTBnetwork.pdf", width=8, height = 11)
plot(CBTBnet, layout="spring", vsize=10)
dev.off()


#Centrality Indices


#centrality plots

pdf(file= "CBTBcentrality.pdf", width=8, height = 11)
centralityPlot(CBTBnet, include = c("ExpectedInfluence", "Strength", "Betweenness", "Closeness"))
dev.off()

#centrality table

centralityTable(CBTBnet)

#Stability


#edge weight accuracy
#use nBoots to override the default of 1,000, tutorial paper uses 2,500.. 1,000 is often sufficient.

#CBT
CBTBboot1<- bootnet(CBTBnet, nCores = 5)
print(CBTBboot1)
summary(CBTBboot1)

pdf(file= "CBTBboot1.pdf", width=8, height = 11)
plot(CBTBboot1, labels= FALSE, order="sample")
dev.off()


#central stability.using case dropping bootstrap
#investigating the stability of centrality indices by estimating network models based on subsets of the data.

#CBT
CBTBboot2<- bootnet(CBTBnet, type="case", nCores=5)
pdf(file= "CBTBboot2.pdf", width=8, height = 11)
plot(CBTBboot2)
dev.off()


#central stability can be quantified using the CS-coefficient, which quantifies the maximum proportions of cases that can be dropped to reatin, with
#95% certainty, a correlation with the originally centrality of higher than (0.7) by default. A cuttoff of 0.5 required to consider the metric stable.

#CBT
corStability(CBTBboot2, cor=0.7, statistics = "all")


#plot the difference tests of node strength between all pairs of edge-weightsplot(boot1, "edge", plot="difference"onlyNonZero= TRUE, order="sample")

#CBT
pdf(file= "CBTBboot1b.pdf", width=11, height = 11)
plot(CBTBboot1, "edge", plot= "difference", onlyNonZero = TRUE, order = "sample")
dev.off()

#comparing node strength
#bootstrapped difference test (a=.05) of node strength of the symptoms. Gray boxes indicate nodes or edges that do not signifcanlty differ, and
#black boxes represent nodes or edges that do differ signifcantly from one another. White boxes show the value of node strength.

#CBT
pdf(file= "CBTBboot1c.pdf", width=11, height = 11)
plot(CBTBboot1, "strength")
dev.off()


#### MADRS data ####

#load CBT AND ADM datasets from csv file

CBTMdat<- read.csv("Complete/CBTMADRS.csv", header= TRUE, sep=",", as.is=TRUE)
View(CBTMdat)

ADMMdat<- read.csv("Complete/ADMSMADRS.csv", header= TRUE, sep=",", as.is=TRUE)
View(ADMMdat)

#estimate networkS EBIC glassoGGM


CBTMnet<- estimateNetwork(CBTMdat, default= "EBICglasso", tuning= 0.0, corMethod = "cor", corArgs = list(method = "spearman"))

pdf(file= "CBTMnetwork.pdf", width=8, height = 11)
plot(CBTMnet, layout="spring", vsize=10)
dev.off()


ADMMnet<- estimateNetwork(ADMMdat, default= "EBICglasso", tuning= 0.0, corMethod = "cor", corArgs = list(method = "spearman"))

pdf(file= "ADMMnetwork.pdf", width=8, height = 11)
plot(ADMMnet, layout="spring", vsize=10)
dev.off()


l<-averageLayout(CBTMnet, ADMMnet)
layout(t(1:2))
pdf(file= "CBTvADMmadrs.pdf", width=8, height = 11)
plot(CBTMnet, layout=l, vsize=8, title= "CBT MADRS",labels= c("AppSad", "ReportSad", "Tension", "Sleep", "Appetite", "Concentration", "Lassitude", "Feel", "Pessimism", "Suicide") )
plot(ADMMnet, layout=l, vsize=8, title= "ADM MADRS", labels= c("AppSad", "ReportSad", "Tension", "Sleep", "Appetite", "Concentration", "Lassitude", "Feel", "Pessimism", "Suicide"))
dev.off()

#Centrality Indices


#centrality plots

pdf(file= "CBTMcentrality.pdf", width=8, height = 11)
centralityPlot(CBTMnet, include = c("ExpectedInfluence", "Strength", "Betweenness", "Closeness"))
dev.off()

pdf(file= "ADMMcentrality.pdf", width=8, height = 11)
centralityPlot(ADMMnet, include = c("ExpectedInfluence", "Strength", "Betweenness", "Closeness"))
dev.off()

#centrality table

centralityTable(CBTMnet)

centralityTable(ADMMnet)


#Stability


#edge weight accuracy
#use nBoots to override the default of 1,000, tutorial paper uses 2,500.. 1,000 is often sufficient.

#CBT
CBTMboot1<- bootnet(CBTMnet, nCores = 5)
print(CBTMboot1)
summary(CBTMboot1)
pdf(file= "CBTMboot1.pdf", width=8, height = 11)
plot(CBTMboot1, labels= FALSE, order="sample")
dev.off()

#ADM
ADMMboot1<- bootnet(ADMMnet, nCores = 5)
print(ADMMboot1)
summary(ADMMboot1)
pdf(file= "ADMMboot1.pdf", width=8, height = 11)
plot(ADMMboot1, labels= FALSE, order="sample")
dev.off()


#central stability.using case dropping bootstrap
#investigating the stability of centrality indices by estimating network models based on subsets of the data.

#CBT
CBTMboot2<- bootnet(CBTMnet, type="case", nCores=5)
pdf(file= "CBTMboot2.pdf", width=8, height = 11)
plot(CBTMboot2)
dev.off()

#ADM
ADMMboot2<- bootnet(ADMMnet, type="case", nCores=5)
pdf(file= "ADMMboot2.pdf", width=8, height = 11)
plot(ADMMboot2)
dev.off()


#central stability can be quantified using the CS-coefficient, which quantifies the maximum proportions of cases that can be dropped to reatin, with
#95% certainty, a correlation with the originally centrality of higher than (0.7) by default. A cuttoff of 0.5 required to consider the metric stable.

#CBT
corStability(CBTMboot2, cor=0.7, statistics = "all")

#ADM
corStability(ADMMboot2, cor=0.7, statistics = "all")

#plot the difference tests of node strength between all pairs of edge-weightsplot(boot1, "edge", plot="difference"onlyNonZero= TRUE, order="sample")

#CBT
pdf(file= "CBTMboot1b.pdf", width=11, height = 11)
plot(CBTMboot1, "edge", plot= "difference", onlyNonZero = TRUE, order = "sample")
dev.off()

#ADM
pdf(file= "ADMMboot1b.pdf", width=11, height = 11)
plot(ADMMboot1, "edge", plot= "difference", onlyNonZero = TRUE, order = "sample")
dev.off()

#comparing node strength
#bootstrapped difference test (a=.05) of node strength of the symptoms. Gray boxes indicate nodes or edges that do not signifcanlty differ, and
#black boxes represent nodes or edges that do differ signifcantly from one another. White boxes show the value of node strength.

#CBT
pdf(file= "CBTMboot1c.pdf", width=11, height = 11)
plot(CBTMboot1, "strength")
dev.off()


#ADM
pdf(file= "ADMMboot1c.pdf", width=11, height = 11)
plot(ADMMboot1, "strength")
dev.off()

#Network Comparison Test for MADRS
#compare networks and store in object called res (results) and print these results.
res<-NCT(CBTMnet, ADMMnet, gamma=0, it=5000, binary.data = FALSE, paired = FALSE, weighted = TRUE, test.edges=TRUE, edges="all", progressbar = TRUE, test.centrality = TRUE)
print(res)
capture.output(res, file="CBTvESCmadrs5000.txt", append = TRUE)

# plot the results of the invariance of network structure as a whole.
#when this is found to be non-significant do not do further testing on specific edges as this inflates multiple testing and type 1 error.
plot(res, what="network")

#plot results of the global stregnth invariance test.
#hypothesis states the overall connectivity is the same accross networks
plot(res, what="strength")
